# Linking atmospheric, terrestrial and aquatic environments: Regime shifts in the Estonian climate over the past 50 years

**DOI:** 10.1371/journal.pone.0209568

**Published:** 2018-12-27

**Authors:** Jonne Kotta, Kristjan Herkül, Jaak Jaagus, Ants Kaasik, Urmas Raudsepp, Victor Alari, Timo Arula, Juta Haberman, Arvo Järvet, Külli Kangur, Are Kont, Ain Kull, Jaan Laanemets, Ilja Maljutenko, Aarne Männik, Peeter Nõges, Tiina Nõges, Henn Ojaveer, Anneliis Peterson, Alvina Reihan, Rein Rõõm, Mait Sepp, Ülo Suursaar, Ottar Tamm, Toomas Tamm, Hannes Tõnisson

**Affiliations:** 1 Estonian Marine Institute, University of Tartu, Tallinn, Estonia; 2 Institute of Ecology and Earth Sciences, University of Tartu, Tartu, Estonia; 3 Marine Systems Institute, Tallinn University of Technology, Tallinn, Estonia; 4 Institute of Agricultural and Environmental Sciences, Estonian University of Life Sciences, Tartu, Estonia; 5 Institute of Ecology, Tallinn University, Tallinn, Estonia; 6 Department of Marine Systems, Tallinn University of Technology, Tallinn, Estonia; 7 Department of Civil Engineering and Architecture, Tallinn University of Technology, Tallinn, Estonia; 8 Institute of Forestry and Rural Engineering, Estonian University of Life Sciences, Tartu, Estonia; Alfred-Wegener-Institut Helmholtz-Zentrum fur Polar- und Meeresforschung, GERMANY

## Abstract

Climate change in recent decades has been identified as a significant threat to natural environments and human wellbeing. This is because some of the contemporary changes to climate are abrupt and result in persistent changes in the state of natural systems; so called regime shifts (RS). This study aimed to detect and analyse the timing and strength of RS in Estonian climate at the half-century scale (1966−2013). We demonstrate that the extensive winter warming of the Northern Hemisphere in the late 1980s was represented in atmospheric, terrestrial, freshwater and marine systems to an extent not observed before or after the event within the studied time series. In 1989, abiotic variables displayed statistically significant regime shifts in atmospheric, river and marine systems, but not in lake and bog systems. This was followed by regime shifts in the biotic time series of bogs and marine ecosystems in 1990. However, many biotic time series lacked regime shifts, or the shifts were uncoupled from large-scale atmospheric circulation. We suggest that the latter is possibly due to complex and temporally variable interactions between abiotic and biotic elements with ecosystem properties buffering biotic responses to climate change signals, as well as being affected by concurrent anthropogenic impacts on natural environments.

## Introduction

Climate change in recent decades has been identified as a significant threat to natural environments and human wellbeing [1, 2, 3]. This is because some of the contemporary changes to climate are abrupt and result in persistent changes in the state of natural systems; so called regime shifts (RS) [4, 5]. There are several concepts of the definition of RS in time series (e.g., [6, 7, 8]). In ecology, a RS often refers to a substantial change in the structure and function of the whole ecosystem [9,10,11]. Such an approach, however, would require the underlying processes that result in the shift in a particular time series to be identified. To date, mechanistic approaches, which identify RSs through parametrizing feedback loops propelling systems toward an alternative state, are not feasible for time series data as we mostly lack consensus on long-term drivers of the physical and biological environments [11]. Instead, the concept of displacement is commonly employed in oceanography and climatology. Here, time series are split into homogeneous sequences that have statistically different mean values relative to their within regime variance [7].Such climatological RSs are being increasingly observed across a large number of physical and biological variables from a number of Earth systems, with the 1980s event representing a major change in the Earth’s biophysical systems around the world [12]. Understanding recent climate change is of vital importance as these variations may inform us about the risks and uncertainties of associated impacts (e.g., [3, 13,14, 15, 16]).

Following a growth in observation networks and advancements in prognostic modelling, the representation of weather and local details of terrestrial and aquatic systems has highly improved. This has motivated a series of studies on how climate change influences key natural and human living conditions (e.g., [17, 12]). Nevertheless, the nature of system-wide climate change phenomena remains inconclusive [18].

To date, the majority of studies have targeted only single components of climate systems (e.g. atmosphere, land, bog, lake, river or sea). Data from different sub-systems have been analysed independently (e.g., [19]) and/or the observed time series have represented different (not necessarily coupled) geographical locations [20, 12, 15]. Moreover, in many climate change assessments, abiotic and biotic variables were often pooled together and thereby those studies failed to isolate ecosystem behaviour from environmental change (but see [21,22]). Thus, there is a need to address how changes in atmospheric processes may cascade down to local weather and contribute to the variability of different climatic sub-systems including their biotic components, with the aim of identifying the nature and strength of linkages between these systems at a regional level.

Estonia represents a highly dynamic climate system of Northern Europe where climate change signals are particularly strong as suggested by several independent atmospheric, terrestrial and aquatic studies (e.g., [23, 24, 25, 26, 27]). These studies show that a large number of physical and biological variables have substantially changed in the region in the last decades and often such transformations were abrupt rather than gradual. In the current study we collated all available regional long-term time series and quantified the timing and strength of RSs in these time series. Then we assessed if and how RSs in atmospheric processes are transferred to local weather and terrestrial, freshwater, and marine systems. Such comparisons enabled us to quantify the magnitude of impacts of the recent climate change phenomena in the whole complex of regional climate, as well as to judge the connectedness of different elements of the climate system elements at a regional level.

## Material and methods

### Description of study area

**Fig 1:**
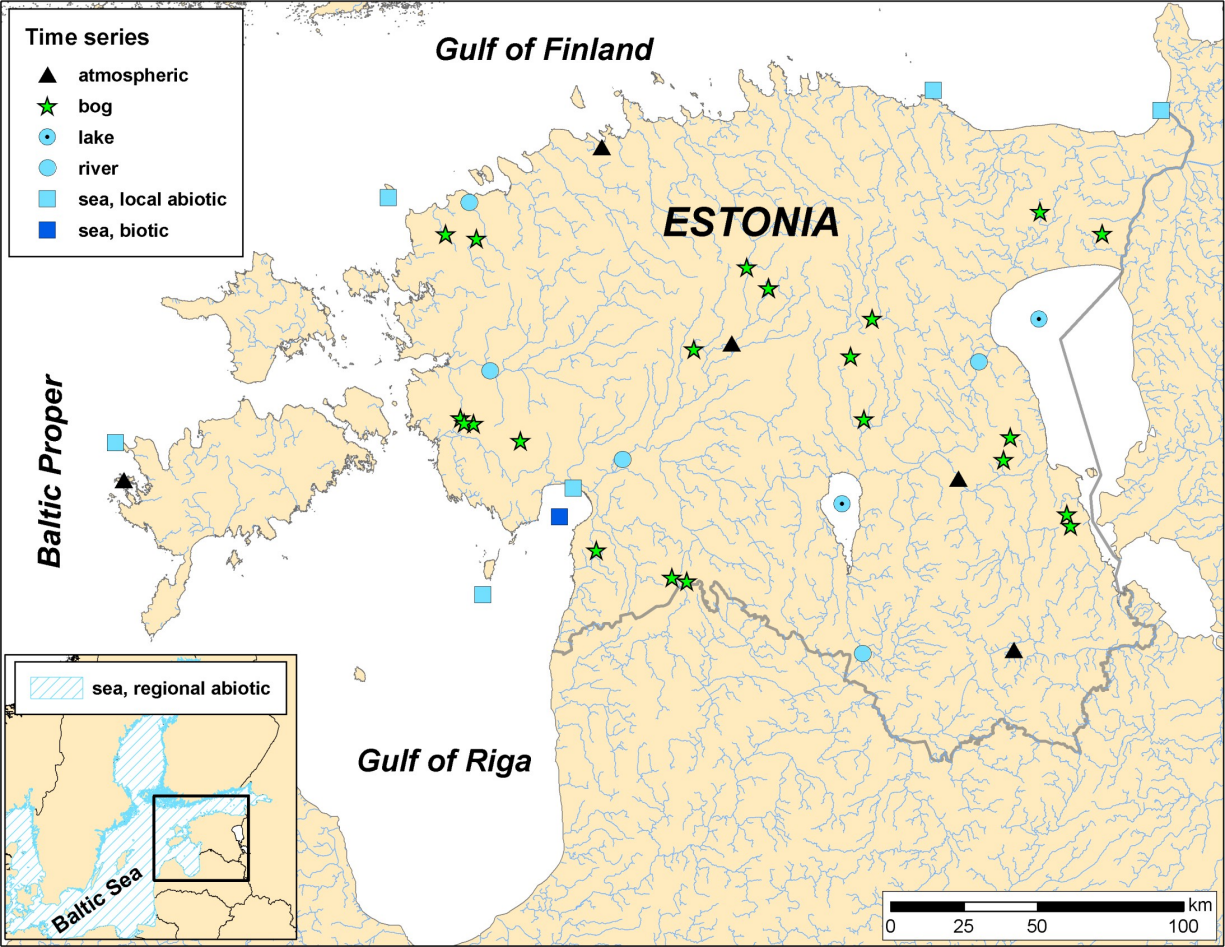
Study area and the locations of time-series by system categories.

The Estonian mainland is situated on the northeastern coast of the Baltic Sea between 57.5 and 59.5°N (Fig 1). It has a long coastline with numerous islands, peninsulas and bays. Its terrain is mostly gently sloping rising up to 300 m above sea level. Climatic conditions are spatially and seasonally very variable due to the influence of different air masses that originate from the North Atlantic, Eurasia and the Arctic. Estonia has a typical transitional climate from maritime in the islands of western Estonia to continental in its eastern parts. Over half of the land is covered by forest, mostly pine and spruce trees, and about one fifth is covered with peatlands. The country also contains a dense river network and about 1200 natural lakes [28].

The epicontinental, semi-enclosed and shallow Baltic Sea (situated between 9–30° E and 53–66° N) is one of the largest brackish water areas of the world. The hydroclimatic conditions of the sea are determined by its position in the transition area between Atlantic marine and Eurasian continental climate systems. It is characterized by steep salinity and temperature gradients, decreasing from southwest to northeast. Owing to its short evolutionary history and brackish conditions, the system is defined by low functional redundancy [29].

### Time series

The present study is based on datasets that represent atmosphere, land, bog, lake, river systems of Estonia and surrounding Baltic Sea basins and cover the period from 1966 to 2013 (Fig 1).

Large-scale atmospheric circulation is the main factor of climate variability in the whole northern Europe including Estonia. This regional circulation pattern can be described adequately by various indices [30]. In this study, we used only the North Atlantic Oscillation (NAO) index as a proxy of the most important circulation patterns in Estonia during the cold season. The NAO indices have been shown to have significant regime shifts in the last decades whereas other circulation indices such as the East Atlantic and the Scandinavia patterns, have had very few regime shifts [31].

Different monthly NAO indices based on standardized sea-level pressure differences between the Azores high and Icelandic low (data from Lisbon https://climatedataguide.ucar.edu/climate-data/hurrell-north-atlantic-oscillation-nao-index-station-based [32], or Gibraltar https://crudata.uea.ac.uk/cru/data/nao/values.htm [33] as well as on principal components (PC) of pressure fields https://climatedataguide.ucar.edu/climate-data/hurrell-north-atlantic-oscillation-nao-index-pc-based [34]) were used to describe the intensity of large-scale westerly circulation.

The local time series data represent key variables spanning from abiotic to biotic environments in these systems. The temporal resolution of the data spans from hours to years. When resolution allowed, the studied time series were decomposed into monthly, seasonal (winter corresponds to DJF, spring to MAM, summer to JJA and autumn SON) and annual means and were then used in the analyses to identify RSs (S1 Table). To reduce random noise and increase generalities of the results, all biotic time series used in statistical analyses represent aggregation of a large number of measurements in space (multiple sampling stations representative to the studied system) and/or time (seasonal or yearly means are derived from weekly or monthly data). Time series were only included into the analyses if the long-term surveys strictly followed the same protocol of sampling and sample analysis, often involving the same personnel or study group throughout the studied period of time. In the studied time series, less than 1% of the data was missing. As variables used in the analyses were monthly (or broader) averages of high-resolution climatic observation, missing data is likely an insignificant source of error in our analyses. Altogether, the 941 studied time series covered NAO (51), atmosphere (293), bog (48), lake (101), river (85), and sea (363). Abiotic time series were more numerous than biotic time series and consisted of 89% of all studied variables (see S1 Table for the list of studied variables and their plotted history).

### Data analyses

In this paper, we employed the concept of displacement [7] to identify RSs in the studied time series. In this concept, different regimes are defined as multi-year periods of time series that are statistically different, relative to their within period variance. Consequently, this approach allows all climate and ecosystem variables to be treated similarly so that linkages in the timing of RSs between all possible pairs of abiotic and biotic variables within and between different climate subsystems can be quantified.

The existing RS methods span from simple statistical comparisons to complex multivariate modelling frameworks; nevertheless, many used techniques are highly sensitive to outliers, the length of time series, and the selected values of model parameters, thereby resulting in situations where real climate variability may be overlooked for false breaks (e.g., [35, 36, 37]).

In our study, we used non-parametric multivariate change point (CP) analysis [38] to identify RSs (i.e. detect distributional changes) in the studied time series. The method is able to estimate multiple CP locations (within a multivariate time series), without a priori knowledge of the number of CPs. This allows comparison of analyses of multivariate time series with different dimension (i.e. blocks of different size) while also screening for changes in dependence (whereas analysis of univariate time series data would likely produce more false positives for larger blocks and changes in dependence could not be detected altogether).

We used the divisive algorithm in the ecp package for R [39]. The algorithm can be outlined as follows. First, the most likely CP location is estimated as the location which maximizes a particular scaled sample measure of divergence (as defined by [40]). Then, the estimated CP is tested for statistical significance using a permutation test. If the CP is statistically significant then the steps are repeated (within the previously formed time series segments) and thus multiple CPs are estimated by iteratively applying a procedure for locating a single CP. In the implementation, we used two years as the minimal possible distance between successive CPs in order to assure that even short shifts are identified. A confidence level of 95% was used to control the false positive CP rate. This is a powerful method to identify the timing of RSs and to assess their significance. It is an effective method because it requires no a priori assumptions about candidate RS years and/or the duration of system states. The method is not sensitive to data from non-normal distributions or data with outliers and has a strong theoretical basis and is easy to use. Full details of this procedure can be found in [38].

Firstly, we detected CPs separately for abiotic and biotic elements of atmospheric, terrestrial, bog, lake, river and marine systems. Then we quantified relatedness of these systems in terms of commonalities in the timing and strength of CPs of each time series (by averaging similarities of all possible pairs of time series of respective systems). Here we used the Agresti’s Adjusted Rand index [41], which is a measure of similarity tailored to compare two sets of estimated CPs (taking into account that the number of CPs in each set need not be equal). CP results were also used for ordination of the individual series and visualizing the intensity patterns of RS in the studied systems. Leave-one-out (LOO) was used for analysing the robustness of the obtained results: from a block with n different series 1 series was dropped and analysis repeated allowing for n different analyses that could then be summarized. R software [42] was used throughout the analysis.

## Results

**Fig 2:**
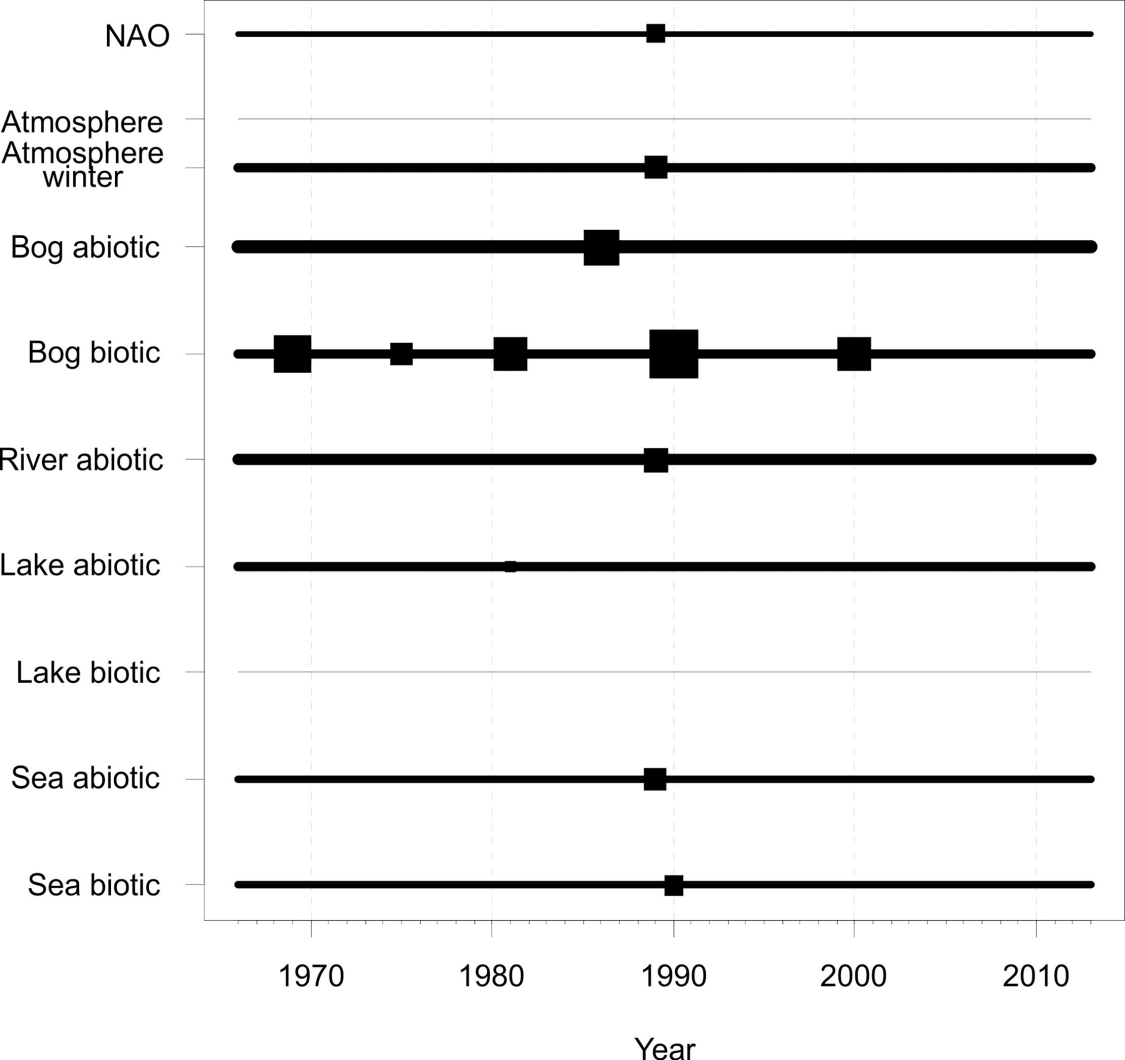
Results of multivariate RS analysis by the studied systems. A timing of statistically significant RSs is shown by filled squares with the size of square indicating the relative strength of RS (i.e. the relative number of individual series within a block that exhibited RS around particular year). Here the RS year indicates the first year of new regime. The thickness of line depicts the relatedness of time series within each system (i.e. the average similarity between the clustered profiles of individual time series and the respective block) and thicker line indicates higher similarities among the studied time series. See Data analyses subsection of Material and methods for further details. For the list of used time series, their original temporal resolution and spatial extent see S1 Table. A script on how to execute the analysis under the R environment are given in the S2 Table.

Following the extensive winter warming of the Northern Hemisphere in 1988−1989, as demonstrated by a significant shift in the NAO indices, many abiotic elements of the Estonian region collectively responded to this climatic RS. The multivariate CP analysis detected statistically significant RSs in winter weather and abiotic variables of river and marine systems but not in bog and lake systems. The abiotic time series of bogs displayed a significant shift in 1986 and lakes in 1981 (Fig 2).

**Fig 3:**
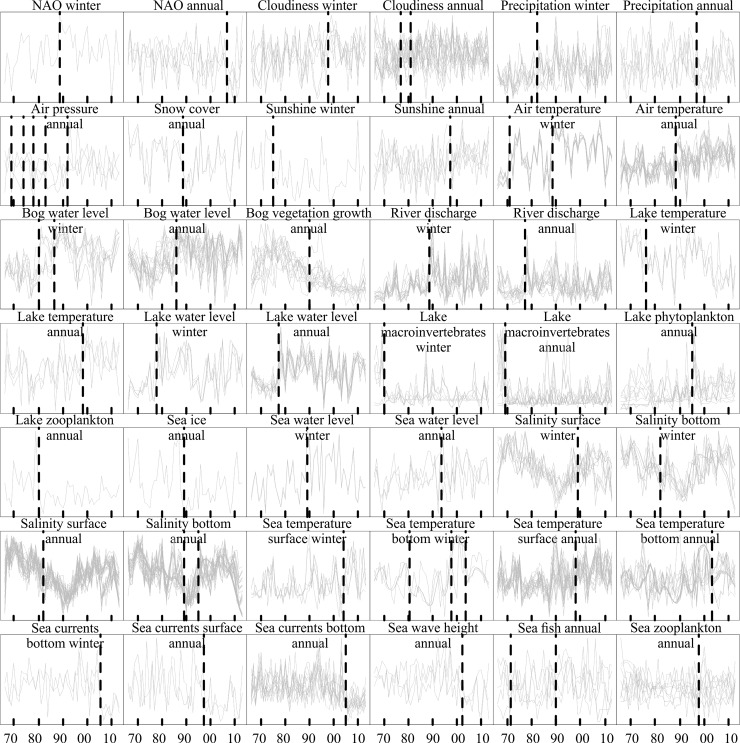
Potted history for each time series that exhibited RSs in the CP analysis. **N**ormalized time series are displayed separately for abiotic and biotic elements of atmospheric, terrestrial, bog, lake, river and marine systems. A timing of typical RSs within different subsystems is shown by broken lines. See Data analyses subsection of Material and methods for further details. More detailed descriptions and timing of RSs of each studied time series are shown in S1 Table.

In 1988−1999, the annual mean surface air temperature significantly increased at all studied weather stations. This was mostly due to a prominent shift from December through April. Consistent with this shift, precipitation raised abruptly at some weather stations in March and April and cloudiness in the winter season or at the yearly scale. Moreover, the yearly duration of snow cover decreased at all studied weather stations (Fig 3, S1 Table).

These atmospheric phenomena were followed by a shift in the peak of river runoff to earlier in the spring, and elevated discharge in all rivers from February to March and reduced discharge in April. In the marine environment, since 1989 ice around coasts nearly disappeared and near-bottom salinity significantly dropped in all studied basins with the strongest effects observed from April to June. Surface water temperature was notably above the long-term average from February through April and resulted in a significant temperature shift in the spring season. Sea level raised significantly at all stations in February coupled with elevated wave height in most west-facing coasts at the yearly scale. In 2004, with a relaxation of westerly circulation, most of the physical variables did not show an abrupt change (i.e. reversal of the RS) but a smooth return to the original state (Fig 3, S1 Table).

In bogs, water levels significantly rose throughout the whole year in 1985−1987 and the reverse RS occurred mostly in winter months in1992−1993. In lakes, water levels abruptly increased in 1977−1978 at monthly, seasonal and yearly scales and gradually returned to the original state in the beginning of 1990s. Moreover, lake water temperature had two distinct phases at the annual scale with higher than average values recorded in 1966−1975 and 1999−2013, and lower than average values in 1976−1998. On the seasonal scale, the water temperature shifted to higher values in autumn 1989 (Fig 3, S1 Table).

In contrast to abiotic time series, the majority of biotic variables did not have RSs in 1988–1989 and there was a lack of any other common pattern in terms of the timing of RSs. In the marine environment, only the first annual appearance of herring larvae shifted earlier in time in 1989, followed by the peaking density of 1-year-old individuals and herring spawning stock biomass in 1990. Moreover, a potential food of the larval herring, the cladoceran *Bosmina* spp., reduced its abundance in the same year when herring stock peaked. The RSs of other time biotic series in the marine environment were independent of the 1988−1989 RS. In 1998 the abundance of goby larvae significantly dropped and this was accompanied with a notable increase in the abundance of their prey copepods (Fig 3, S1 Table).

The timing of RSs varied largely among different bogs; however, there were some commonalities. Except for one bog, the radial growth increment of Scots pines had a high growth phase in the 1970s and partly in the 1980s. There was a significant reduction in growth from the 1980s to nowadays with strongest reduction taking place in 1990 (Fig 3, S1 Table).

In contrast to other climate subsystems, the multivariate CP analysis did not identify any significant RS in biotic time series from lakes. Nevertheless, individual time series showed some resemblance but there were large differences between benthic (bottom sediments) and pelagic (water) time series. The abundance and biomass of benthic invertebrates, mostly molluscs and insect larvae, significantly declined in 1970. In addition, the invertebrate biomasses peaked in cold water seasons (from autumn through spring) in 1987. Concurrent with the decrease in the biomass of benthic invertebrates in 1970, pelagic cyanobacteria, mostly *Snowella* and *Planktolyngbya* increased their abundances. The abundance of cyanobacteria peaked further in 1995 due to the elevated abundances of *Aphanizomenon*, *Microcystis* and *Limnothrix*. Moreover, the abundance of pelagic copepod grazers declined in 1980, accompanied by an increase in water chlorophyll *a* (i.e. a proxy for phytoplankton biomass) in 1983. Then the rotifer grazers significantly declined in 2004, the water chlorophyll *a* peaked further in 2006, and the abundance of the cyanobacteria *Snowella* increased in 2007 (Fig 3, S1 Table).

**Fig 4:**
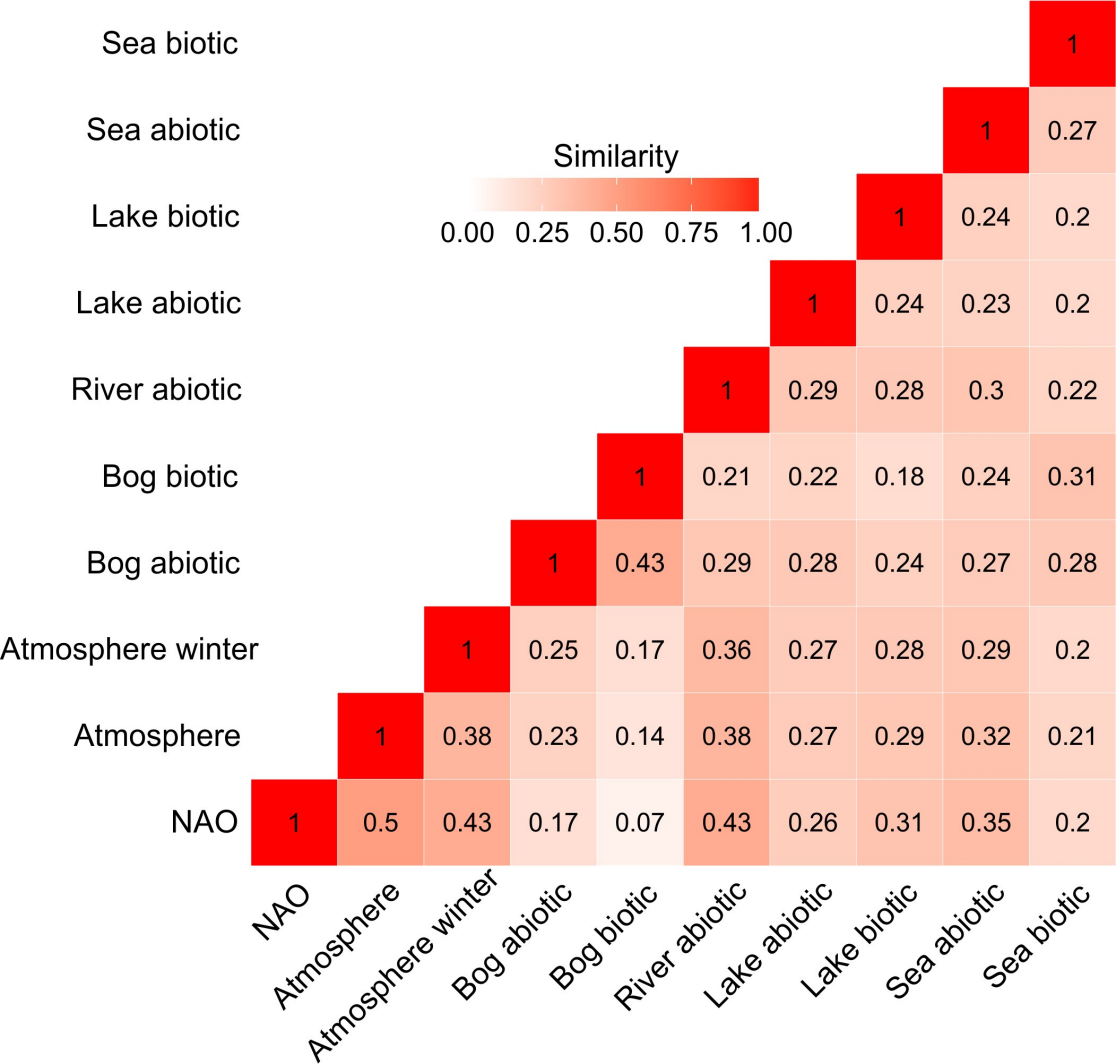
Relatedness of different components of the regional climate system in terms of the timing and strength of RSs during 1966−2013. Each value represents an average similarity of all possible pairs of time series between the respective studied systems with higher values indicating higher similarity as measured by the Agresti’s Adjusted Rand index. NAO indices represent global drivers of change, and a wide range of abiotic and biotic time series of atmospheric, terrestrial, bog, lake, river and marine systems are regional responders of such global change. More detailed descriptions of each studied time series within different studied systems are shown in S1 Table.

**Fig 5:**
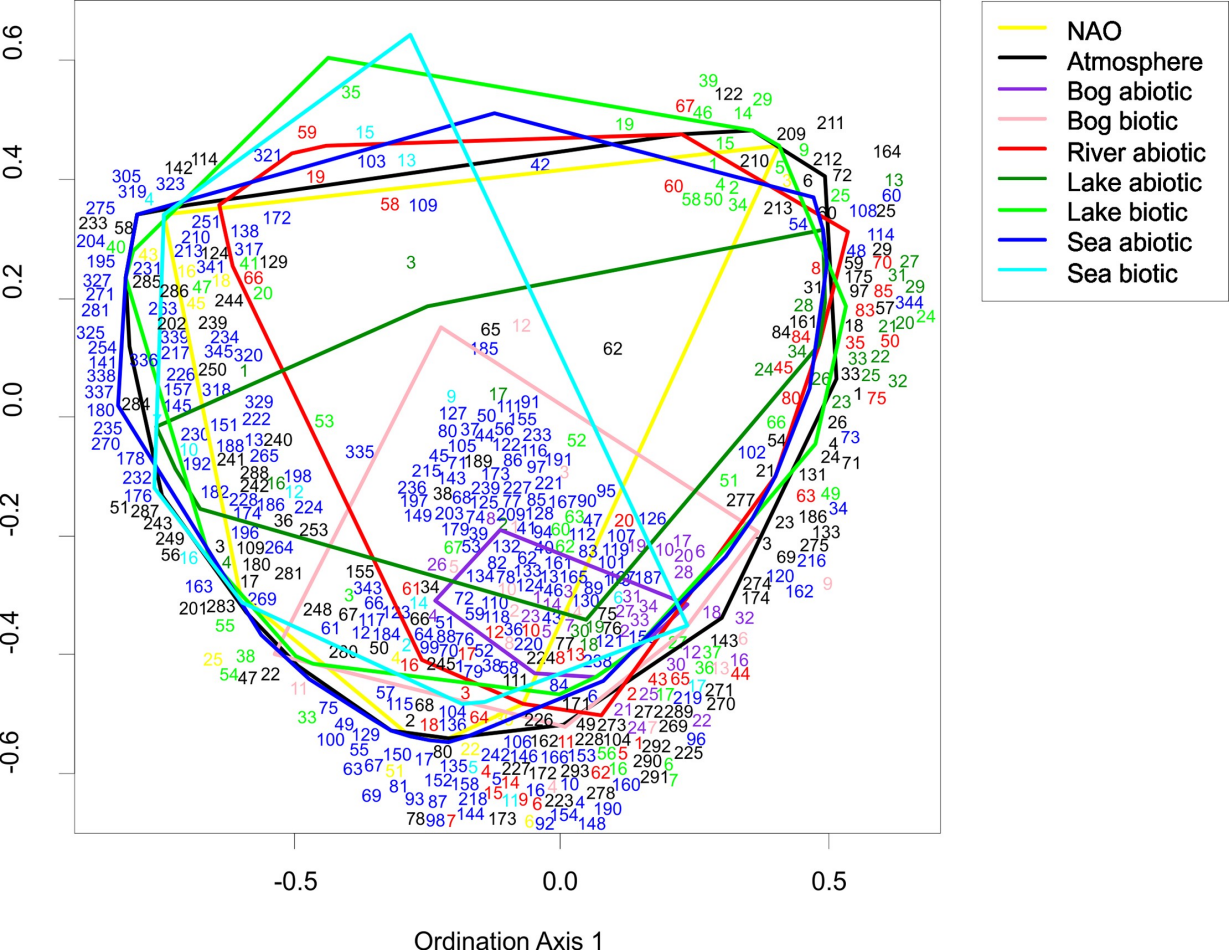
Ordination of the individual time series of different components of the regional climate system in terms of the timing and strength of RSs during 1966−2013. Here the closer distance between time series shows closer resemblances. NAO indices represent the atmospheric circulation driver of change, and a wide range of abiotic and biotic time series of atmospheric, terrestrial, bog, lake, river and marine systems are regional responders of such large-scale change. The coloured polygons depict the range of variability of time series in different studied systems. The codes of time-series are explained in S1 Table.

A multivariate comparison of the studied systems suggested that the behaviour of regional atmospheric processes, local weather and river hydrology are highly interlinked. On the other hand, the signal of the regional climate processes transfers only partially into lake and marine hydrophysical fields and very poorly into the bog abiotic environment. In general, regional atmospheric processes and biotic components of the studied systems were only weakly connected. If regional atmospheric processes and lake biological time series were moderately coupled then the biotic time series of bog and marine systems did not show such a linkage (Fig 4). As shown by an ordination of the individual time series, the behaviour of biotic time-series often showed a high degree of independence and were linked to different forcing variables, not necessarily related to the regional atmospheric processes (Fig 5). Here the closer distance between time series shows closer resemblances in terms of the timing and strength of RSs. As seen from the figure, most studied systems are similar in terms of the range of variability of time series except for bogs (both abiotic and biotic) and the biotic components of marine system. Within each system, time-series show a large variability with different time-series showing differential affinity to different forcing variables.

The multivariate CP analysis was shown to be robust in a sense that any single series was not overly influential. When a single time series was randomly dropped and full analysis based on the reduced block was carried out (leave-one-out or LOO) the repeatability of RSs for each system was close to 100% (Table 1).

**Table 1:** Robustness analysis of RS detection. Within each system category a single time series was dropped and full CP analysis based on the reduced block was carried out (leave-one-out or LOO). This was repeated for all single time series. Thus e.g. for the NAO category 51 different multivariate datasets were used, each consisting of 50 time-series. LOO RS repeatability shows the percent of LOO repetitions where a RS was detected within +/-2 years of the RS detected on the full dataset (displayed on Fig 2). LOO relatedness deviation shows the standard deviation of the mean Agresti’s Adjusted Rand index.

System category	LOO RS repeatability	LOO relatedness deviation
NAO	100%	0.04
Atmosphere	-	0
Atmosphere winter	100%	0.06
Bog abiotic	100%	0.02
Bog biotic	100%/100%/100%/100%/100%	0.03
River abiotic	100%	0.01
Lake abiotic	97%	0.02
Lake biotic	-	0
Sea abiotic	100%	0
Sea biotic	94%	0.10

## Discussion

### Abiotic patterns

In this study we embraced a whole-system approach to assess how RSs in atmospheric processes are represented in terrestrial, freshwater, coastal and marine environments in Estonia. The aim was to identify potential linkages within and between these climate subsystems during 1966−2013. We demonstrated that a RS in 1989 represented the major change in Estonian climate and this RS cascaded down to local weather as well as abiotic time-series of river and marine systems. We also showed that following a relaxation of westerly circulation, most of the physical variables returned smoothly (i.e. without reverse RSs) to their original state. This suggests that abiotic elements of individual systems of the Estonian region are well coupled with the large-scale atmospheric circulation.

The 1989 RS was due to an intensification of westerly circulation over the North Atlantic and Europe in February and March [43,5,44] that brought much warmer and moister maritime air from the ocean to the continent and caused milder weather conditions [45]. Following the shift in atmospheric conditions, mean air temperature increased by 2–3 degrees and milder winters with unstable snow cover became more frequent. Due to more frequent melting events, river discharges notably increased from January to March and decreased in April. The latter was largely due to a reduced accumulation of precipitation as snow cover. The observed winter warming also produced an elevated water temperature in the Baltic Sea.

Following the RS in atmospheric circulation, the average airflow speed notably increased in January to March [45,46]. Developments in wind conditions, in turn, affected wave conditions and sea level [47]. The effects were strongest at coastal locations exposed to the west because in such areas the directions of the longest effective fetches and the predominant storm winds coincide. Similarly, RSs were evident in the sea level data, especially in bays where water can pile up as a result of strong and prolonged westerly winds.

RSs in wave patterns, rising sea-levels and the reduced duration of protective ice-cover in the late 1980s intensified the dynamics of shore processes four times. Major changes were due to a rapid increase in areas of erosion and redeposition of the eroded sediments [48]. Those shores that face north quickly reached a new equilibrium state whereas others facing west are still undergoing pronounced change [49]. The latter is likely due to elevated wave velocities in the westerly exposed coastal areas[50].

RS in atmospheric circulation in 1988−1989 resulted in an abrupt reduction of salinity in coastal environments. This was due to a shifting balance in the amounts and frequencies of saline water inflows from the North Sea through the Danish Straits, and precipitation-fed riverine (freshwater) inflow [51]. However, such a linkage is not necessarily straightforward as none of the RSs in atmospheric circulation before 1988−1989 produced any detectable RS in salinity conditions. Salinity conditions are formed by intermittent pulses of salty North Sea water pushed into the Baltic Sea that are caused by daily variability in high- and low-pressure systems over the larger North Atlantic region [52]. These sporadic pulses are more related to extreme weather events rather than persistent RSs in the atmospheric circulation. Locally, however, modified wind conditions may completely change water circulation patterns and facilitate the transport of salty and nutrient-rich deep water to the surface productive layer [53]; thus, trends in the opposite directions may also occur.

The importance of the 1989 RS has been previously shown for specific elements of the regional climate system including the detection of shifts in atmospheric circulation [43], meteorological regime [45], severity of winters [54], precipitation [45], seasonal river runoff [55, 56], cumulative wind stress [57], sea level and maximum ice cover [54]. These studies did not, however, inform us of the magnitude of RSs in the climate system and the degree to which the processes are reversible. In fact, some studies even concluded that evidence of a climate change signal is not decisive (e.g., [58]). Similarly, our study also pointed out that the coupling between atmospheric processes and regional climate has some uncertainty caused by high variability of responses within each system. Although the multivariate CP analysis detected the presence of significant RSs in the studied systems in 1989, individual time-series within each system often showed a large deviation from this pattern.

### Biotic patterns

**Fig 6:**
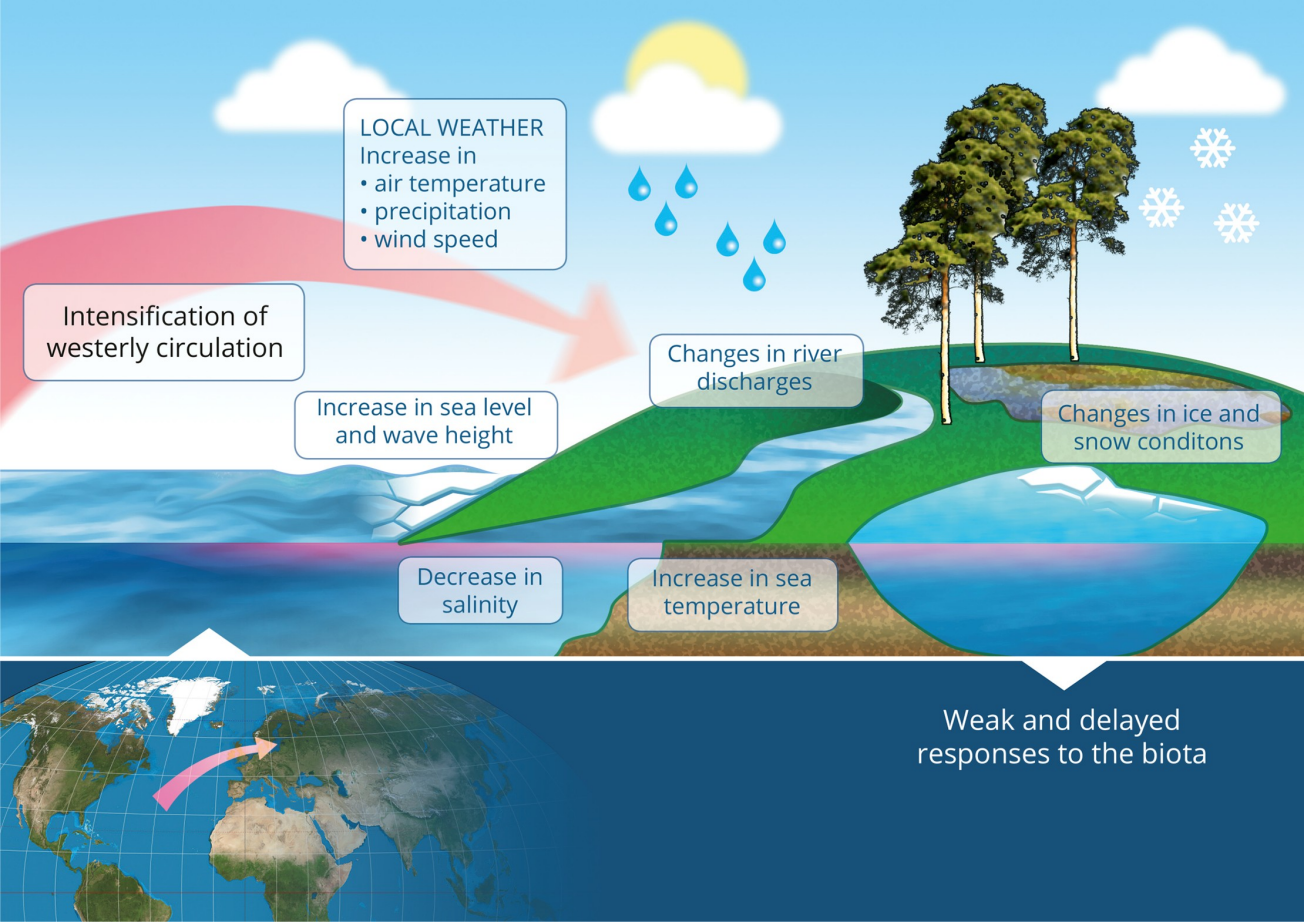
Schematic diagram of the impact of the 1989 RS in atmospheric circulation on abiotic and biotic components of different systems of the Estonian regional climate.

Our analyses showed that the signal of large-scale atmospheric circulation in 1989 is cascading only weakly to biotic time series (Fig 6). Weak linkages between abiotic and biotic components of the ecosystems may arise from multiple causes. Firstly, it is plausible that recent changes in mean temperature are ecologically unimportant as large seasonal variations counteract the potential effects of recent global warming. Secondly, ecosystems respond to multiple drivers controlled by external forcing that does not exhibit shifting behaviour, and therefore the observed time series may not show such behaviour (e.g. the effect of solar activity on limnetic systems, [59]. Thirdly, the ecosystems themselves have properties to buffer responses of biota to a climate change signal [11,60].

In limnetic systems, the most prominent climate induced changes are often due to shifts in surface water temperature and an emergence of extreme temperature events, especially in shallow basins [26]. However, the morphometric variability and local conditions add large uncertainty to the response to climatic signals (e.g. [61,62]. In the studied lakes, water level is acknowledged to define the trajectory of ecosystem development through modifying sediment resuspension, internal loading of nutrients, and underwater light conditions and spawning conditions for fish [63]. Thus, shifts between phases of high and low water levels are behind many of the observed RSs in biotic time series in the early 1980s and the mid 1990s. Moreover, the measures of fisheries management that target the composition of fish communities, and the balance between predatory and non-predatory fish species, cascade down through the food web [64]. Such reorganization of fisheries management of the lake food webs took place in the 1970s. After the banning of small-meshed fishing gear in the 1970s, pikeperch biomass increased, rotifers become smaller, copepods become larger, the biomass of phytoplankton decreased and ultimately the biomass of benthic invertebrates declined. Nonetheless, the food web interactions of eutrophic lakes are very complex, all relationships are blurred by the multiple interfering factors in the system, and thereby reasons of some of the observed RSs are still not yet explained.

There are multiple examples from marine environments of heatwaves causing blooms of algae [65], the replacement of keystone species [66]and other major shifts in community structure [67, 68]. Some of these ecosystems have moderate annual temperature fluctuations where already a limited temperature increase for a prolonged time period likely pushes species beyond their optimal temperature range and thereby leads to RSs with multiple potential alternate states involved [69]. In the Baltic Sea, however, large seasonal variations counteract the effects of recent warming and a seasonal increase of a few degrees in seawater temperature does not transfer to many observable RSs in biota. An appearance of herring larvae two weeks earlier in 1989 is an example of a significant response of sea water warming to the biota, and this RS is explained by the earlier break-up of ice [70]. Then, with a lag of one year, the density of 1-year-old individuals and herring spawning stock biomass peaked. As herring larvae are food limited in the study area [71] a potential food of the larval herring, the cladoceran *Bosmina* spp., reduced its abundance in the same year when herring stock peaked and the copepods showed very low abundances throughout the 1990s. Similar RSs have been shown in marine pelagic systems in the central Baltic Sea [72] but also at the global scale [73]. However, our study also showed that most of biotic responses were largely uncoupled from the regional atmospheric circulation as many of the pelagic time series and all benthic time series lacked a clear response to the 1989 RS.

RSs in atmospheric circulation did not cascade down to the radial increment of pine trees in bogs. Here, pine trees are adapted to large natural variations in water level and high nutrient content, which are not defined by large-scale atmospheric circulation but rather directly affected by human induced drainage [74]. Intensive drainage is expected to result in lowered water level, improved aeration of peat water, and increased pH and nutrient availability due to peat mineralisation. Ultimately, the growth of trees is enhanced. Large-scale drainage of mires took place in Estonia between the 1950s and the 1980s, and according to a recent inventory, the drained area has halved since then [75]. The growth of pine trees clearly reflects the shifting intensity in the draining of bogs with higher growth phase observed in the 1970s and partly in the 1980s, and lower growth phase since the 1990s.

The atmospheric RSs also involved significant changes in precipitation and river discharge patterns. The latter, in turn, significantly affects nutrient loads to lakes and coastal environments and thereby regional atmospheric processes, in interaction with the developments of human society, control nutrient pools and primary productivity in aquatic ecosystems (e.g., [76, 77]. However, coincident trends between climate and human activities prevent their clear quantitative separation [78]. Moreover, the studied period from 1966 to 2013 encompasses profound alterations in patterns of human population, agricultural and industrial practices and eutrophication. Such an overarching change in anthropogenic impacts on the natural environment is expected to confound linkages between large-scale atmospheric forcing and biota. This calls for future RS studies to incorporate time series of fundamental anthropogenic factors (e.g. land use, type and intensity of agriculture, maritime transport, municipal wastewater pollution) in order to gain a more complete understanding of changes in natural systems [79].

Biotic components themselves may determine how a system responds to changes in the non-living environment. The actual consequences of external and internal forcings are not straightforward as biologically-mediated couplings (e.g. primary production, grazing and predation) respond in multiple ways to changed forcing variables, and thereby could sustain, increase, or reduce current coupling as well as potentially even introduce new coupling pathways [80, 81]. Overall, biological time series have inherently greater unpredictability in their behaviour than non-living components of the regional climatic sub-systems.

The stability of food webs forms the backbone of most natural ecosystems and specific structural features are responsible for such stability. Large complex networks are less likely to be stable compared to simpler networks [82]. However, if food web links are weak and/or predators are not very specialized, the stability of an ecosystem is strongly increased [83, 11]. This is the case in the Baltic Sea where many species tolerate broad ranges of environmental conditions and are generalist feeders (e.g., [84, 85]. Consequently, they are capable of compensating for shifting dominances by switching grazing/predation to new species and thereby keeping the ecosystem in a given regime. Similarly, in lake ecosystems, food-web interactions are behind their strong resilience [86]. However, if climate change causes a mismatch in the development of consumers and their resources, a RS may cascade through food-webs [87, 88]. Warming may also strengthen trophic cascades from fish to primary producers, shifting the control of primary production toward stronger top-down and weaker bottom-up effects, and thus stabilize some ecosystem processes [89]. Seemingly, in our biotic time series, the magnitude of disturbance that was absorbed into ecosystems due to the recent abrupt warming of the Northern Hemisphere did not exceed a critical threshold where the whole system collapses and shifts to a different regime.

In the current paper we only used the NAO indices as a large-scale atmospheric circulation forcing. This mode is the most important circulation pattern in Estonia during the cold season. During the warm season, however, the influence of the atmospheric teleconnection patterns on weather conditions is quite low in Estonia [24]. We have recently studied trends and regime shifts in several atmospheric circulation patterns (Arctic Oscillation, East Atlantic, East Atlantic/West Russia, Scandinavia, Polar/Eurasia patterns) and their relationships with climate variability in Estonia [31]. The latter study also showed a very few trends and regime shifts during the warm season mostly represented by a clear signal of the East Atlantic pattern. But its upward shifts in 1998 (July) and 2009 (August) were largely uncoupled from shifts in environmental variables presented in the current paper.

## Conclusions

Recent studies point out that RSs are natural phenomena in various ocean, freshwater, and terrestrial systems (e.g., [90,12] with evidence for a major RS in the late 1980s across the whole climate system of northern Europe (e.g., [91, 92, 12]). The natural environment of Estonia has been extensively studied and documented since the mid 20^th^ century. A critical re-evaluation of all time series collected from the Estonian region demonstrated that many abiotic components of terrestrial and aquatic systems respond to RSs in atmospheric processes. However, due to many factors and complex interactions involved, only a few of the biological time series examined had RSs in the late 1980s and therefore the reported RSs cannot be considered system-wide (in sensu [93]). This suggests a presence of strong publication bias towards positive findings whilst ignoring a large number of regional biotic time series with no RS [94, 95]. Publication bias matters in climate change and other studies because literature reviews regarding support for a hypothesis and ultimately the policy advice that is based on such a synthesis can be biased if the original literature is contaminated by results that only show a significant finding.

Nevertheless, a systematic lack of RS in biotic time series does not imply the absence of impacts of contemporary climate change on the biota. In order to quantify the associated impacts on the studied ecosystems a priori biological knowledge, existing time series and experimental evidence should be combined into a single modelling framework capable of resolving different types of responses (not just RS). In such a model, experiments provide us reliable cause-effect relationships between key ecosystem elements under current environmental conditions and future climate change. Time-series, in turn, allow us to extract information on heterogeneous distributions and associated uncertainties. Ultimately, these models will inform us on all known and unknown aspects of how contemporary climate change impacts the biota and provide credible guidance on how uncertainties related to the climate change effects can be most effectively reduced.

## Supporting information

S1 Table List of used time series, their original temporal resolution and spatial extent. Below potted history for each variable with identified regime shifts as broken lines. Number at the broken line reflects the statistical significance of the particular regime shift.
(PDF)

S2 Table A R script on how to depict a timing and relative strength of statistically significant RSs and the relatedness of time series within each system category (i.e. abiotic and biotic elements of atmospheric, terrestrial, bog, lake, river and marine systems) in terms of the timing of RSs. (PDF)
