# Supplementary material for: Linking atmospheric, terrestrial and aquatic environments: Regime shifts in the Estonian climate over the past 50 years
Source: PLoS One. 2018 Dec 27;13(12):e0209568. doi: 10.1371/journal.pone.0209568 (PMC6307728; doi:10.1371/journal.pone.0209568)
Supplement: S1 Table — Below potted history for each variable with identified regime shifts as broken lines. Number at the broken line reflects the statistical significance of the particular regime shift. (PDF) [file pone.0209568.s001.pdf]

S1 Table. List of used time series, their original temporal resultion and spatial extent. Below potted history for each variable with identified regime shifts as broken lines. Number at the broken line reflects the statistical significance of the particular regime shift.

Block name	Variable number in the block	Variable name	Unit	Original temporal resolution	Spatial extent	Reference
NAO	1	Gibraltar	index	daily	Atlantic Ocean	Jones et al. 1997
NAO	2	NAO January average Lissabon	index	daily	Atlantic Ocean	Hurrell, 1995
NAO	3	NAO January average PC based	index	daily	Atlantic Ocean	Hurrell, 1995
NAO	4	NAO February average Gibraltar	index	daily	Atlantic Ocean	Hurrell 2013
NAO	5	NAO February average Lissabon	index	daily	Atlantic Ocean	Jones et al. 1997
NAO	6	NAO February average PC based	index	daily	Atlantic Ocean	Hurrell, 1995
NAO	7	NAO March average Gibraltar	index	daily	Atlantic Ocean	Hurrell, 1995
NAO	8	NAO March average Lissabon	index	daily	Atlantic Ocean	Jones et al. 1997
NAO	9	NAO March average PC based	index	daily	Atlantic Ocean	Hurrell, 1995
NAO	10	NAO April average Gibraltar	index	daily	Atlantic Ocean	Hurrell, 2013
NAO	11	NAO April average Lissabon	index	daily	Atlantic Ocean	Jones et al. 1997
NAO	12	NAO April average PC based	index	daily	Atlantic Ocean	Hurrell, 1995
NAO	13	NAO May average Gibraltar	index	daily	Atlantic Ocean	Hurrell, 2013
NAO	14	NAO May average Lissabon	index	daily	Atlantic Ocean	Jones et al. 1997
NAO	15	NAO May average PC based	index	daily	Atlantic Ocean	Hurrell, 1995
NAO	16	NAO June average Gibraltar	index	daily	Atlantic Ocean	Hurrell, 2013
NAO	17	NAO June average Lissabon	index	daily	Atlantic Ocean	Jones et al. 1997
NAO	18	NAO June average PC based	index	daily	Atlantic Ocean	Hurrell, 1995
NAO	19	NAO July average Gibraltar	index	daily	Atlantic Ocean	Hurrell, 2013
NAO	20	NAO July average Lissabon	index	daily	Atlantic Ocean	Jones et al. 1997
NAO	21	NAO July average PC based	index	daily	Atlantic Ocean	Hurrell, 1995
NAO	22	Gibraltar	index	daily	Atlantic Ocean	Hurrell, 2013

NAO	23	Lissabon	index	daily	Atlantic Ocean	Jones et al. 1997
NAO	24	NAO August average PC based	index	daily	Atlantic Ocean	Hurrell, 1995
NAO	25	NAO September average Gibraltar	index	daily	Atlantic Ocean	Hurrell, 2013
NAO	26	NAO September average Lissabon	index	daily	Atlantic Ocean	Jones et al. 1997
NAO	27	NAO September average PC based	index	daily	Atlantic Ocean	Hurrell, 1995
NAO	28	NAO October average Gibraltar	index	daily	Atlantic Ocean	Hurrell, 2013
NAO	29	NAO October average Lissabon	index	daily	Atlantic Ocean	Jones et al. 1997
NAO	30	NAO October average PC based	index	daily	Atlantic Ocean	Hurrell, 1995
NAO	31	NAO November average Gibraltar	index	daily	Atlantic Ocean	Hurrell, 2013
NAO	32	NAO November average Lissabon	index	daily	Atlantic Ocean	Jones et al. 1997
NAO	33	NAO November average PC based	index	daily	Atlantic Ocean	Hurrell, 1995
NAO	34	NAO December average Gibraltar	index	daily	Atlantic Ocean	Hurrell, 2013
NAO	35	NAO December average Lissabon	index	daily	Atlantic Ocean	Jones et al. 1997
NAO	36	NAO December average PC based	index	daily	Atlantic Ocean	Hurrell, 1995
NAO	37	NAO winter average Gibraltar	index	daily	Atlantic Ocean	Hurrell, 2013
NAO	38	NAO winter average Lissabon	index	daily	Atlantic Ocean	Jones et al. 1997
NAO	39	NAO winter average PC based	index	daily	Atlantic Ocean	Hurrell, 1995
NAO	40	NAO spring average Gibraltar	index	daily	Atlantic Ocean	Hurrell, 2013

NAO	41	NAO spring average Lissabon	index	daily	Atlantic Ocean	Jones et al. 1997
NAO	42	NAO spring average PC based	index	daily	Atlantic Ocean	Hurrell, 1995
NAO	43	NAO summer average Gibraltar	index	daily	Atlantic Ocean	Hurrell, 2013
NAO	44	NAO summer average Lissabon	index	daily	Atlantic Ocean	Jones et al. 1997
NAO	45	NAO summer average PC based	index	daily	Atlantic Ocean	Hurrell, 1995
NAO	46	NAO autumn average Gibraltar	index	daily	Atlantic Ocean	Hurrell, 2013
NAO	47	NAO autumn average Lissabon	index	daily	Atlantic Ocean	Jones et al. 1997
NAO	48	NAO autumn average PC based	index	daily	Atlantic Ocean	Hurrell, 1995
NAO	49	NAO yearly average Gibraltar	index	daily	Atlantic Ocean	Hurrell, 2013
NAO	50	NAO yearly average Lissabon	index	daily	Atlantic Ocean	Jones et al. 1997
NAO	51	NAO yearly average PC based	index	daily	Atlantic Ocean	Hurrell, 1995
Atmosphere	1	Cloud cover January average Võru	fraction in tenths	daily	station	Estonian Weather Service, 2016a; Russak and Kallis, 2003
Atmosphere	2	Cloud cover January average Vilsandi	fraction in tenths	daily	station	Estonian Weather Service, 2016a; Russak and Kallis, 2003
Atmosphere	3	Cloud cover January average Tallinn	fraction in tenths	daily	station	Estonian Weather Service, 2016a; Russak and Kallis, 2003
Atmosphere	4	Cloud cover January average Tartu	fraction in tenths	daily	station	Estonian Weather Service, 2016a; Russak and Kallis, 2003
Atmosphere	5	Cloud cover February average Võru	fraction in tenths	daily	station	Estonian Weather Service, 2016a; Russak and Kallis, 2003
Atmosphere	6	Cloud cover February average Vilsandi	fraction in tenths	daily	station	Estonian Weather Service, 2016a; Russak and Kallis, 2003
Atmosphere	7	Cloud cover February average Tallinn	fraction in tenths	daily	station	Estonian Weather Service, 2016a; Russak and Kallis, 2003

Atmosphere	8	Cloud cover February average Tartu	fraction in tenths	daily	station	Estonian Weather Service, 2016a; Russak and Kallis, 2003
Atmosphere	9	Cloud cover March average Võru	fraction in tenths	daily	station	Estonian Weather Service, 2016a; Russak and Kallis, 2003
Atmosphere	10	Cloud cover March average Vilsandi	fraction in tenths	daily	station	Estonian Weather Service, 2016a; Russak and Kallis, 2003
Atmosphere	11	Cloud cover March average Tallinn	fraction in tenths	daily	station	Estonian Weather Service, 2016a; Russak and Kallis, 2003
Atmosphere	12	Cloud cover March average Tartu	fraction in tenths	daily	station	Estonian Weather Service, 2016a; Russak and Kallis, 2003
Atmosphere	13	Cloud cover April average Võru	fraction in tenths	daily	station	Estonian Weather Service, 2016a; Russak and Kallis, 2003
Atmosphere	14	Cloud cover April average Vilsandi	fraction in tenths	daily	station	Estonian Weather Service, 2016a; Russak and Kallis, 2003
Atmosphere	15	Cloud cover April average Tallinn	fraction in tenths	daily	station	Estonian Weather Service, 2016a; Russak and Kallis, 2003
Atmosphere	16	Cloud cover April average Tartu	fraction in tenths	daily	station	Estonian Weather Service, 2016a; Russak and Kallis, 2003
Atmosphere	17	Cloud cover May average Võru	fraction in tenths	daily	station	Estonian Weather Service, 2016a; Russak and Kallis, 2003
Atmosphere	18	Cloud cover May average Vilsandi	fraction in tenths	daily	station	Estonian Weather Service, 2016a; Russak and Kallis, 2003
Atmosphere	19	Cloud cover May average Tallinn	fraction in tenths	daily	station	Estonian Weather Service, 2016a; Russak and Kallis, 2003
Atmosphere	20	Cloud cover May average Tartu	fraction in tenths	daily	station	Estonian Weather Service, 2016a; Russak and Kallis, 2003
Atmosphere	21	Cloud cover June average Võru	fraction in tenths	daily	station	Estonian Weather Service, 2016a; Russak and Kallis, 2003
Atmosphere	22	Cloud cover June average Vilsandi	fraction in tenths	daily	station	Estonian Weather Service, 2016a; Russak and Kallis, 2003

Atmosphere	23	Cloud cover June average Tallinn	fraction in tenths	daily	station	Estonian Weather Service, 2016a; Russak and Kallis, 2003
Atmosphere	24	Cloud cover June average Tartu	fraction in tenths	daily	station	Estonian Weather Service, 2016a; Russak and Kallis, 2003
Atmosphere	25	Cloud cover July average Võru	fraction in tenths	daily	station	Estonian Weather Service, 2016a; Russak and Kallis, 2003
Atmosphere	26	Cloud cover July average Vilsandi	fraction in tenths	daily	station	Estonian Weather Service, 2016a; Russak and Kallis, 2003
Atmosphere	27	Cloud cover July average Tallinn	fraction in tenths	daily	station	Estonian Weather Service, 2016a; Russak and Kallis, 2003
Atmosphere	28	Cloud cover July average Tartu	fraction in tenths	daily	station	Estonian Weather Service, 2016a; Russak and Kallis, 2003
Atmosphere	29	Cloud cover August average Võru	fraction in tenths	daily	station	Estonian Weather Service, 2016a; Russak and Kallis, 2003
Atmosphere	30	Cloud cover August average Vilsandi	fraction in tenths	daily	station	Estonian Weather Service, 2016a; Russak and Kallis, 2003
Atmosphere	31	Cloud cover August average Tallinn	fraction in tenths	daily	station	Estonian Weather Service, 2016a; Russak and Kallis, 2003
Atmosphere	32	Cloud cover August average Tartu	fraction in tenths	daily	station	Estonian Weather Service, 2016a; Russak and Kallis, 2003
Atmosphere	33	Cloud cover September average Võru	fraction in tenths	daily	station	Estonian Weather Service, 2016a; Russak and Kallis, 2003
Atmosphere	34	Cloud cover September average Vilsandi	fraction in tenths	daily	station	Estonian Weather Service, 2016a; Russak and Kallis, 2003
Atmosphere	35	Cloud cover September average Tallinn	fraction in tenths	daily	station	Estonian Weather Service, 2016a; Russak and Kallis, 2003
Atmosphere	36	Cloud cover September average Tartu	fraction in tenths	daily	station	Estonian Weather Service, 2016a; Russak and Kallis, 2003
Atmosphere	37	Cloud cover October average Võru	fraction in tenths	daily	station	Estonian Weather Service, 2016a; Russak and Kallis, 2003

Atmosphere	38	Cloud cover October average Vilsandi	fraction in tenths	daily	station	Estonian Weather Service, 2016a; Russak and Kallis, 2003
Atmosphere	39	Cloud cover October average Tallinn	fraction in tenths	daily	station	Estonian Weather Service, 2016a; Russak and Kallis, 2003
Atmosphere	40	Cloud cover October average Tartu	fraction in tenths	daily	station	Estonian Weather Service, 2016a; Russak and Kallis, 2003
Atmosphere	41	Cloud cover November average Võru	fraction in tenths	daily	station	Estonian Weather Service, 2016a; Russak and Kallis, 2003
Atmosphere	42	Cloud cover November average Vilsandi	fraction in tenths	daily	station	Estonian Weather Service, 2016a; Russak and Kallis, 2003
Atmosphere	43	Cloud cover November average Tallinn	fraction in tenths	daily	station	Estonian Weather Service, 2016a; Russak and Kallis, 2003
Atmosphere	44	Cloud cover November average Tartu	fraction in tenths	daily	station	Estonian Weather Service, 2016a; Russak and Kallis, 2003
Atmosphere	45	Cloud cover December average Võru	fraction in tenths	daily	station	Estonian Weather Service, 2016a; Russak and Kallis, 2003
Atmosphere	46	Cloud cover December average Vilsandi	fraction in tenths	daily	station	Estonian Weather Service, 2016a; Russak and Kallis, 2003
Atmosphere	47	Cloud cover December average Tallinn	fraction in tenths	daily	station	Estonian Weather Service, 2016a; Russak and Kallis, 2003
Atmosphere	48	Cloud cover December average Tartu	fraction in tenths	daily	station	Estonian Weather Service, 2016a; Russak and Kallis, 2003
Atmosphere	49	Cloud cover winter average Võru	fraction in tenths	daily	station	Estonian Weather Service, 2016a; Russak and Kallis, 2003
Atmosphere	50	Cloud cover winter average Vilsandi	fraction in tenths	daily	station	Estonian Weather Service, 2016a; Russak and Kallis, 2003
Atmosphere	51	Cloud cover winter average Tallinn	fraction in tenths	daily	station	Estonian Weather Service, 2016a; Russak and Kallis, 2003
Atmosphere	52	Cloud cover winter average Tartu	fraction in tenths	daily	station	Estonian Weather Service, 2016a; Russak and Kallis, 2003

Atmosphere	53	Cloud cover spring average Võru	fraction in tenths	daily	station	Estonian Weather Service, 2016a; Russak and Kallis, 2003
Atmosphere	54	Cloud cover spring average Vilsandi	fraction in tenths	daily	station	Estonian Weather Service, 2016a; Russak and Kallis, 2003
Atmosphere	55	Cloud cover spring average Tallinn	fraction in tenths	daily	station	Estonian Weather Service, 2016a; Russak and Kallis, 2003
Atmosphere	56	Cloud cover spring average Tartu	fraction in tenths	daily	station	Estonian Weather Service, 2016a; Russak and Kallis, 2003
Atmosphere	57	Cloud cover summer average Võru	fraction in tenths	daily	station	Estonian Weather Service, 2016a; Russak and Kallis, 2003
Atmosphere	58	Cloud cover summer average Vilsandi	fraction in tenths	daily	station	Estonian Weather Service, 2016a; Russak and Kallis, 2003
Atmosphere	59	Cloud cover summer average Tallinn	fraction in tenths	daily	station	Estonian Weather Service, 2016a; Russak and Kallis, 2003
Atmosphere	60	Cloud cover summer average Tartu	fraction in tenths	daily	station	Estonian Weather Service, 2016a; Russak and Kallis, 2003
Atmosphere	61	Cloud cover autumn average Võru	fraction in tenths	daily	station	Estonian Weather Service, 2016a; Russak and Kallis, 2003
Atmosphere	62	Cloud cover autumn average Vilsandi	fraction in tenths	daily	station	Estonian Weather Service, 2016a; Russak and Kallis, 2003
Atmosphere	63	Cloud cover autumn average Tallinn	fraction in tenths	daily	station	Estonian Weather Service, 2016a; Russak and Kallis, 2003
Atmosphere	64	Cloud cover autumn average Tartu	fraction in tenths	daily	station	Estonian Weather Service, 2016a; Russak and Kallis, 2003
Atmosphere	65	Cloud cover yearly average Võru	fraction in tenths	daily	station	Estonian Weather Service, 2016a; Russak and Kallis, 2003
Atmosphere	66	Cloud cover yearly average Vilsandi	fraction in tenths	daily	station	Estonian Weather Service, 2016a; Russak and Kallis, 2003
Atmosphere	67	Cloud cover yearly average Tallinn	fraction in tenths	daily	station	Estonian Weather Service, 2016a; Russak and Kallis, 2003

Atmosphere	68	Cloud cover yearly average Tartu	fraction in tenths	daily	station	Estonian Weather Service, 2016a; Russak and Kallis, 2003
Atmosphere	69	Precipitation January average Tartu	mm	daily	station	Männik et al. 2015; Estonian Weather Service, 2016a; Jaagus et al. 2017
Atmosphere	70	Precipitation January average Tallinn	mm	daily	station	Männik et al. 2015; Estonian Weather Service, 2016a; Jaagus et al. 2017
Atmosphere	71	Precipitation January average Türi	mm	daily	station	Männik et al. 2015; Estonian Weather Service, 2016a; Jaagus et al. 2017
Atmosphere	72	Precipitation January average Vilsandi	mm	daily	station	Männik et al. 2015; Estonian Weather Service, 2016a; Jaagus et al. 2017
Atmosphere	73	Precipitation January average Võru	mm	daily	station	Männik et al. 2015; Estonian Weather Service, 2016a; Jaagus et al. 2017
Atmosphere	74	Precipitation February average Tartu	mm	daily	station	Männik et al. 2015; Estonian Weather Service, 2016a; Jaagus et al. 2017
Atmosphere	75	Precipitation February average Tallinn	mm	daily	station	Männik et al. 2015; Estonian Weather Service, 2016a; Jaagus et al. 2017
Atmosphere	76	Precipitation February average Türi	mm	daily	station	Männik et al. 2015; Estonian Weather Service, 2016a; Jaagus et al. 2017
Atmosphere	77	Precipitation February average Vilsandi	mm	daily	station	Männik et al. 2015; Estonian Weather Service, 2016a; Jaagus et al. 2017
Atmosphere	78	Precipitation February average Võru	mm	daily	station	Männik et al. 2015; Estonian Weather Service, 2016a; Jaagus et al. 2017
Atmosphere	79	Precipitation March average Tartu	mm	daily	station	Männik et al. 2015; Estonian Weather Service, 2016a; Jaagus et al. 2017
Atmosphere	80	Precipitation March average Tallinn	mm	daily	station	Männik et al. 2015; Estonian Weather Service, 2016a; Jaagus et al. 2017
Atmosphere	81	Precipitation March average Türi	mm	daily	station	Männik et al. 2015; Estonian Weather Service, 2016a; Jaagus et al. 2017
Atmosphere	82	Precipitation March average Vilsandi	mm	daily	station	Männik et al. 2015; Estonian Weather Service, 2016a; Jaagus et al. 2017

Atmosphere	83	Precipitation March average Võru	mm	daily	station	Männik et al. 2015; Estonian Weather Service, 2016a; Jaagus et al. 2017
Atmosphere	84	Precipitation April average Tartu	mm	daily	station	Männik et al. 2015; Estonian Weather Service, 2016a; Jaagus et al. 2017
Atmosphere	85	Precipitation April average Tallinn	mm	daily	station	Männik et al. 2015; Estonian Weather Service, 2016a; Jaagus et al. 2017
Atmosphere	86	Precipitation April average Türi	mm	daily	station	Männik et al. 2015; Estonian Weather Service, 2016a; Jaagus et al. 2017
Atmosphere	87	Precipitation April average Vilsandi	mm	daily	station	Männik et al. 2015; Estonian Weather Service, 2016a; Jaagus et al. 2017
Atmosphere	88	Precipitation April average Võru	mm	daily	station	Männik et al. 2015; Estonian Weather Service, 2016a; Jaagus et al. 2017
Atmosphere	89	Precipitation May average Tartu	mm	daily	station	Männik et al. 2015; Estonian Weather Service, 2016a; Jaagus et al. 2017
Atmosphere	90	Precipitation May average Tallinn	mm	daily	station	Männik et al. 2015; Estonian Weather Service, 2016a; Jaagus et al. 2017
Atmosphere	91	Precipitation May average Türi	mm	daily	station	Männik et al. 2015; Estonian Weather Service, 2016a; Jaagus et al. 2017
Atmosphere	92	Precipitation May average Vilsandi	mm	daily	station	Männik et al. 2015; Estonian Weather Service, 2016a; Jaagus et al. 2017
Atmosphere	93	Precipitation May average Võru	mm	daily	station	Männik et al. 2015; Estonian Weather Service, 2016a; Jaagus et al. 2017
Atmosphere	94	Precipitation June average Tartu	mm	daily	station	Männik et al. 2015; Estonian Weather Service, 2016a; Jaagus et al. 2017
Atmosphere	95	Precipitation June average Tallinn	mm	daily	station	Männik et al. 2015; Estonian Weather Service, 2016a; Jaagus et al. 2017
Atmosphere	96	Precipitation June average Türi	mm	daily	station	Männik et al. 2015; Estonian Weather Service, 2016a; Jaagus et al. 2017
Atmosphere	97	Precipitation June average Vilsandi	mm	daily	station	Männik et al. 2015; Estonian Weather Service, 2016a; Jaagus et al. 2017

Atmosphere	98	Precipitation June average Võru	mm	daily	station	Männik et al. 2015; Estonian Weather Service, 2016a; Jaagus et al. 2017
Atmosphere	99	Precipitation July average Tartu	mm	daily	station	Männik et al. 2015; Estonian Weather Service, 2016a; Jaagus et al. 2017
Atmosphere	100	Precipitation July average Tallinn	mm	daily	station	Männik et al. 2015; Estonian Weather Service, 2016a; Jaagus et al. 2017
Atmosphere	101	Precipitation July average Türi	mm	daily	station	Männik et al. 2015; Estonian Weather Service, 2016a; Jaagus et al. 2017
Atmosphere	102	Precipitation July average Vilsandi	mm	daily	station	Männik et al. 2015; Estonian Weather Service, 2016a; Jaagus et al. 2017
Atmosphere	103	Precipitation July average Võru	mm	daily	station	Männik et al. 2015; Estonian Weather Service, 2016a; Jaagus et al. 2017
Atmosphere	104	Precipitation August average Tartu	mm	daily	station	Männik et al. 2015; Estonian Weather Service, 2016a; Jaagus et al. 2017
Atmosphere	105	Precipitation August average Tallinn	mm	daily	station	Männik et al. 2015; Estonian Weather Service, 2016a; Jaagus et al. 2017
Atmosphere	106	Precipitation August average Türi	mm	daily	station	Männik et al. 2015; Estonian Weather Service, 2016a; Jaagus et al. 2017
Atmosphere	107	Precipitation August average Vilsandi	mm	daily	station	Männik et al. 2015; Estonian Weather Service, 2016a; Jaagus et al. 2017
Atmosphere	108	Precipitation August average Võru	mm	daily	station	Männik et al. 2015; Estonian Weather Service, 2016a; Jaagus et al. 2017
Atmosphere	109	Precipitation September average Tartu	mm	daily	station	Männik et al. 2015; Estonian Weather Service, 2016a; Jaagus et al. 2017
Atmosphere	110	Precipitation September average Tallinn	mm	daily	station	Männik et al. 2015; Estonian Weather Service, 2016a; Jaagus et al. 2017
Atmosphere	111	Precipitation September average Türi	mm	daily	station	Männik et al. 2015; Estonian Weather Service, 2016a; Jaagus et al. 2017
Atmosphere	112	Precipitation September average Vilsandi	mm	daily	station	Männik et al. 2015; Estonian Weather Service, 2016a; Jaagus et al. 2017

Atmosphere	113	Precipitation September average Võru	mm	daily	station	Männik et al. 2015; Estonian Weather Service, 2016a; Jaagus et al. 2017
Atmosphere	114	Precipitation October average Tartu	mm	daily	station	Männik et al. 2015; Estonian Weather Service, 2016a; Jaagus et al. 2017
Atmosphere	115	Precipitation October average Tallinn	mm	daily	station	Männik et al. 2015; Estonian Weather Service, 2016a; Jaagus et al. 2017
Atmosphere	116	Precipitation October average Türi	mm	daily	station	Männik et al. 2015; Estonian Weather Service, 2016a; Jaagus et al. 2017
Atmosphere	117	Precipitation October average Vilsandi	mm	daily	station	Männik et al. 2015; Estonian Weather Service, 2016a; Jaagus et al. 2017
Atmosphere	118	Precipitation October average Võru	mm	daily	station	Männik et al. 2015; Estonian Weather Service, 2016a; Jaagus et al. 2017
Atmosphere	119	Precipitation November average Tartu	mm	daily	station	Männik et al. 2015; Estonian Weather Service, 2016a; Jaagus et al. 2017
Atmosphere	120	Precipitation November average Tallinn	mm	daily	station	Männik et al. 2015; Estonian Weather Service, 2016a; Jaagus et al. 2017
Atmosphere	121	Precipitation November average Türi	mm	daily	station	Männik et al. 2015; Estonian Weather Service, 2016a; Jaagus et al. 2017
Atmosphere	122	Precipitation November average Vilsandi	mm	daily	station	Männik et al. 2015; Estonian Weather Service, 2016a; Jaagus et al. 2017
Atmosphere	123	Precipitation November average Võru	mm	daily	station	Männik et al. 2015; Estonian Weather Service, 2016a; Jaagus et al. 2017
Atmosphere	124	Precipitation December average Tartu	mm	daily	station	Männik et al. 2015; Estonian Weather Service, 2016a; Jaagus et al. 2017
Atmosphere	125	Precipitation December average Tallinn	mm	daily	station	Männik et al. 2015; Estonian Weather Service, 2016a; Jaagus et al. 2017
Atmosphere	126	Precipitation December average Türi	mm	daily	station	Männik et al. 2015; Estonian Weather Service, 2016a; Jaagus et al. 2017
Atmosphere	127	Precipitation December average Vilsandi	mm	daily	station	Männik et al. 2015; Estonian Weather Service, 2016a; Jaagus et al. 2017

Atmosphere	128	Precipitation December average Võru	mm	daily	station	Männik et al. 2015; Estonian Weather Service, 2016a; Jaagus et al. 2017
Atmosphere	129	Precipitation winter average Tartu	mm	daily	station	Männik et al. 2015; Estonian Weather Service, 2016a; Jaagus et al. 2017
Atmosphere	130	Precipitation winter average Tallinn	mm	daily	station	Männik et al. 2015; Estonian Weather Service, 2016a; Jaagus et al. 2017
Atmosphere	131	Precipitation winter average Türi	mm	daily	station	Männik et al. 2015; Estonian Weather Service, 2016a; Jaagus et al. 2017
Atmosphere	132	Precipitation winter average Vilsandi	mm	daily	station	Männik et al. 2015; Estonian Weather Service, 2016a; Jaagus et al. 2017
Atmosphere	133	Precipitation winter average Võru	mm	daily	station	Männik et al. 2015; Estonian Weather Service, 2016a; Jaagus et al. 2017
Atmosphere	134	Precipitation spring average Tartu	mm	daily	station	Männik et al. 2015; Estonian Weather Service, 2016a; Jaagus et al. 2017
Atmosphere	135	Precipitation spring average Tallinn	mm	daily	station	Männik et al. 2015; Estonian Weather Service, 2016a; Jaagus et al. 2017
Atmosphere	136	Precipitation spring average Türi	mm	daily	station	Männik et al. 2015; Estonian Weather Service, 2016a; Jaagus et al. 2017
Atmosphere	137	Precipitation spring average Vilsandi	mm	daily	station	Männik et al. 2015; Estonian Weather Service, 2016a; Jaagus et al. 2017
Atmosphere	138	Precipitation spring average Võru	mm	daily	station	Männik et al. 2015; Estonian Weather Service, 2016a; Jaagus et al. 2017
Atmosphere	139	Precipitation summer average Tartu	mm	daily	station	Männik et al. 2015; Estonian Weather Service, 2016a; Jaagus et al. 2017
Atmosphere	140	Precipitation summer average Tallinn	mm	daily	station	Männik et al. 2015; Estonian Weather Service, 2016a; Jaagus et al. 2017
Atmosphere	141	Precipitation summer average Türi	mm	daily	station	Männik et al. 2015; Estonian Weather Service, 2016a; Jaagus et al. 2017
Atmosphere	142	Precipitation summer average Vilsandi	mm	daily	station	Männik et al. 2015; Estonian Weather Service, 2016a; Jaagus et al. 2017

Atmosphere	143	Precipitation summer average Võru	mm	daily	station	Männik et al. 2015; Estonian Weather Service, 2016a; Jaagus et al. 2017
Atmosphere	144	Precipitation autumn average Tartu	mm	daily	station	Männik et al. 2015; Estonian Weather Service, 2016a; Jaagus et al. 2017
Atmosphere	145	Precipitation autumn average Tallinn	mm	daily	station	Männik et al. 2015; Estonian Weather Service, 2016a; Jaagus et al. 2017
Atmosphere	146	Precipitation autumn average Türi	mm	daily	station	Männik et al. 2015; Estonian Weather Service, 2016a; Jaagus et al. 2017
Atmosphere	147	Precipitation autumn average Vilsandi	mm	daily	station	Männik et al. 2015; Estonian Weather Service, 2016a; Jaagus et al. 2017
Atmosphere	148	Precipitation autumn average Võru	mm	daily	station	Männik et al. 2015; Estonian Weather Service, 2016a; Jaagus et al. 2017
Atmosphere	149	Precipitation yearly average Tartu	mm	daily	station	Männik et al. 2015; Estonian Weather Service, 2016a; Jaagus et al. 2017
Atmosphere	150	Precipitation yearly average Tallinn	mm	daily	station	Männik et al. 2015; Estonian Weather Service, 2016a; Jaagus et al. 2017
Atmosphere	151	Precipitation yearly average Türi	mm	daily	station	Männik et al. 2015; Estonian Weather Service, 2016a; Jaagus et al. 2017
Atmosphere	152	Precipitation yearly average Vilsandi	mm	daily	station	Männik et al. 2015; Estonian Weather Service, 2016a; Jaagus et al. 2017
Atmosphere	153	Precipitation yearly average Võru	mm	daily	station	Männik et al. 2015; Estonian Weather Service, 2016a; Jaagus et al. 2017
Atmosphere	154	Air pressure January average Tartu	hPa	daily	station	Estonian Weather Service, 2016a
Atmosphere	155	Air pressure February average Tartu	hPa	daily	station	Estonian Weather Service, 2016a
Atmosphere	156	Air pressure March average Tartu	hPa	daily	station	Estonian Weather Service, 2016a
Atmosphere	157	Air pressure April average Tartu	hPa	daily	station	Estonian Weather Service, 2016a
Atmosphere	158	Tartu	hPa	daily	station	Estonian Weather Service, 2016a

Atmosphere	159	Tartu	hPa	daily	station	Estonian Weather Service, 2016a
Atmosphere	160	Tartu	hPa	daily	station	Estonian Weather Service, 2016a
Atmosphere	161	Air pressure August average Tartu	hPa	daily	station	Estonian Weather Service, 2016a
Atmosphere	162	Air pressure September average Tartu	hPa	daily	station	Estonian Weather Service, 2016a
Atmosphere	163	Air pressure October average Tartu	hPa	daily	station	Estonian Weather Service, 2016a
Atmosphere	164	Air pressure November average Tartu	hPa	daily	station	Estonian Weather Service, 2016a
Atmosphere	165	Air pressure December average Tartu	hPa	daily	station	Estonian Weather Service, 2016a
Atmosphere	166	Air pressure winter average Tartu	hPa	daily	station	Estonian Weather Service, 2016a
Atmosphere	167	Air pressure spring average Tartu	hPa	daily	station	Estonian Weather Service, 2016a
Atmosphere	168	Air pressure summer average Tartu	hPa	daily	station	Estonian Weather Service, 2016a
Atmosphere	169	Air pressure autumn average Tartu	hPa	daily	station	Estonian Weather Service, 2016a
Atmosphere	170	Air pressure yearly average Tartu	hPa	daily	station	Estonian Weather Service, 2016a
Atmosphere	171	Duration of snow cover yearly average Tartu	days per year	daily	station	Estonian Weather Service, 2016a; Jaagus et al. 2017
Atmosphere	172	Duration of snow cover yearly average Võru	days per year	daily	station	Estonian Weather Service, 2016a; Jaagus et al. 2017
Atmosphere	173	Duration of snow cover yearly average Tallinn	days per year	daily	station	Estonian Weather Service, 2016a; Jaagus et al. 2017
Atmosphere	174	Duration of snow cover yearly average Vilsandi	days per year	daily	station	Estonian Weather Service, 2016a; Jaagus et al. 2017

Atmosphere	175	Cumulative sunshine duration January average Tartu	h	hourly	station	Estonian Weather Service, 2016a; Russak and Kallis, 2003
Atmosphere	176	Cumulative sunshine duration January average Vilsandi	h	hourly	station	Estonian Weather Service, 2016a; Russak and Kallis, 2003
Atmosphere	177	Cumulative sunshine duration February average Tartu	h	hourly	station	Estonian Weather Service, 2016a; Russak and Kallis, 2003
Atmosphere	178	Cumulative sunshine duration February average Vilsandi	h	hourly	station	Estonian Weather Service, 2016a; Russak and Kallis, 2003
Atmosphere	179	Cumulative sunshine duration March average Tartu	h	hourly	station	Estonian Weather Service, 2016a; Russak and Kallis, 2003
Atmosphere	180	Cumulative sunshine duration March average Vilsandi	h	hourly	station	Estonian Weather Service, 2016a; Russak and Kallis, 2003
Atmosphere	181	Cumulative sunshine duration April average Tartu	h	hourly	station	Estonian Weather Service, 2016a; Russak and Kallis, 2003
Atmosphere	182	Cumulative sunshine duration April average Vilsandi	h	hourly	station	Estonian Weather Service, 2016a; Russak and Kallis, 2003
Atmosphere	183	Cumulative sunshine duration May average Tartu	h	hourly	station	Estonian Weather Service, 2016a; Russak and Kallis, 2003
Atmosphere	184	Cumulative sunshine duration May average Vilsandi	h	hourly	station	Estonian Weather Service, 2016a; Russak and Kallis, 2003
Atmosphere	185	Cumulative sunshine duration June average Tartu	h	hourly	station	Estonian Weather Service, 2016a; Russak and Kallis, 2003
Atmosphere	186	Cumulative sunshine duration June average Vilsandi	h	hourly	station	Estonian Weather Service, 2016a; Russak and Kallis, 2003
Atmosphere	187	Cumulative sunshine duration July average Tartu	h	hourly	station	Estonian Weather Service, 2016a; Russak and Kallis, 2003
Atmosphere	188	Cumulative sunshine duration July average Vilsandi	h	hourly	station	Estonian Weather Service, 2016a; Russak and Kallis, 2003
Atmosphere	189	Cumulative sunshine duration August average Tartu	h	hourly	station	Estonian Weather Service, 2016a; Russak and Kallis, 2003

Atmosphere	190	Cumulative sunshine duration August average Vilsandi	h	hourly	station	Estonian Weather Service, 2016a; Russak and Kallis, 2003
Atmosphere	191	Cumulative sunshine duration September average Tartu	h	hourly	station	Estonian Weather Service, 2016a; Russak and Kallis, 2003
Atmosphere	192	Cumulative sunshine duration September average Vilsandi	h	hourly	station	Estonian Weather Service, 2016a; Russak and Kallis, 2003
Atmosphere	193	Cumulative sunshine duration October average Tartu	h	hourly	station	Estonian Weather Service, 2016a; Russak and Kallis, 2003
Atmosphere	194	Cumulative sunshine duration October average Vilsandi	h	hourly	station	Estonian Weather Service, 2016a; Russak and Kallis, 2003
Atmosphere	195	Cumulative sunshine duration November average Tartu	h	hourly	station	Estonian Weather Service, 2016a; Russak and Kallis, 2003
Atmosphere	196	Cumulative sunshine duration November average Vilsandi	h	hourly	station	Estonian Weather Service, 2016a; Russak and Kallis, 2003
Atmosphere	197	Cumulative sunshine duration December average Tartu	h	hourly	station	Estonian Weather Service, 2016a; Russak and Kallis, 2003
Atmosphere	198	Cumulative sunshine duration December average Vilsandi	h	hourly	station	Estonian Weather Service, 2016a; Russak and Kallis, 2003
Atmosphere	199	Cumulative sunshine duration winter average Tartu	h	hourly	station	Estonian Weather Service, 2016a; Russak and Kallis, 2003
Atmosphere	200	Cumulative sunshine duration winter average Vilsandi	h	hourly	station	Estonian Weather Service, 2016a; Russak and Kallis, 2003
Atmosphere	201	Cumulative sunshine duration spring average Tartu	h	hourly	station	Estonian Weather Service, 2016a; Russak and Kallis, 2003
Atmosphere	202	Cumulative sunshine duration spring average Vilsandi	h	hourly	station	Estonian Weather Service, 2016a; Russak and Kallis, 2003
Atmosphere	203	Cumulative sunshine duration summer average Tartu	h	hourly	station	Estonian Weather Service, 2016a; Russak and Kallis, 2003
Atmosphere	204	Cumulative sunshine duration summer average Vilsandi	h	hourly	station	Estonian Weather Service, 2016a; Russak and Kallis, 2003

Atmosphere	205	Cumulative sunshine duration autumn average Tartu	h	hourly	station	Estonian Weather Service, 2016a; Russak and Kallis, 2003
Atmosphere	206	Cumulative sunshine duration autumn average Vilsandi	h	hourly	station	Estonian Weather Service, 2016a; Russak and Kallis, 2003
Atmosphere	207	Cumulative sunshine duration yearly average Tartu	h	hourly	station	Estonian Weather Service, 2016a; Russak and Kallis, 2003
Atmosphere	208	Cumulative sunshine duration yearly average Vilsandi	h	hourly	station	Estonian Weather Service, 2016a; Russak and Kallis, 2003
Atmosphere	209	Air temperature January average Tallinn	°C	daily	station	Männik et al. 2015; Estonian Weather Service, 2016a; Jaagus et al. 2017
Atmosphere	210	Air temperature January average Tartu	°C	daily	station	Männik et al. 2015; Estonian Weather Service, 2016a; Jaagus et al. 2017
Atmosphere	211	Air temperature January average Võru	°C	daily	station	Männik et al. 2015; Estonian Weather Service, 2016a; Jaagus et al. 2017
Atmosphere	212	Air temperature January average Türi	°C	daily	station	Männik et al. 2015; Estonian Weather Service, 2016a; Jaagus et al. 2017
Atmosphere	213	Air temperature January average Vilsandi	°C	daily	station	Männik et al. 2015; Estonian Weather Service, 2016a; Jaagus et al. 2017
Atmosphere	214	Air temperature February average Tallinn	°C	daily	station	Männik et al. 2015; Estonian Weather Service, 2016a; Jaagus et al. 2017
Atmosphere	215	Air temperature February average Tartu	°C	daily	station	Männik et al. 2015; Estonian Weather Service, 2016a; Jaagus et al. 2017
Atmosphere	216	Air temperature February average Võru	°C	daily	station	Männik et al. 2015; Estonian Weather Service, 2016a; Jaagus et al. 2017
Atmosphere	217	Air temperature February average Türi	°C	daily	station	Männik et al. 2015; Estonian Weather Service, 2016a; Jaagus et al. 2017
Atmosphere	218	Air temperature February average Vilsandi	°C	daily	station	Männik et al. 2015; Estonian Weather Service, 2016a; Jaagus et al. 2017
Atmosphere	219	Air temperature March average Tallinn	°C	daily	station	Männik et al. 2015; Estonian Weather Service, 2016a; Jaagus et al. 2017

Atmosphere	220	Air temperature March average Tartu	°C	daily	station	Männik et al. 2015; Estonian Weather Service, 2016a; Jaagus et al. 2017
Atmosphere	221	Air temperature March average Võru	°C	daily	station	Männik et al. 2015; Estonian Weather Service, 2016a; Jaagus et al. 2017
Atmosphere	222	Air temperature March average Türi	°C	daily	station	Männik et al. 2015; Estonian Weather Service, 2016a; Jaagus et al. 2017
Atmosphere	223	Air temperature March average Vilsandi	°C	daily	station	Männik et al. 2015; Estonian Weather Service, 2016a; Jaagus et al. 2017
Atmosphere	224	Air temperature April average Tallinn	°C	daily	station	Männik et al. 2015; Estonian Weather Service, 2016a; Jaagus et al. 2017
Atmosphere	225	Air temperature April average Tartu	°C	daily	station	Männik et al. 2015; Estonian Weather Service, 2016a; Jaagus et al. 2017
Atmosphere	226	Air temperature April average Võru	°C	daily	station	Männik et al. 2015; Estonian Weather Service, 2016a; Jaagus et al. 2017
Atmosphere	227	Air temperature April average Türi	°C	daily	station	Männik et al. 2015; Estonian Weather Service, 2016a; Jaagus et al. 2017
Atmosphere	228	Air temperature April average Vilsandi	°C	daily	station	Männik et al. 2015; Estonian Weather Service, 2016a; Jaagus et al. 2017
Atmosphere	229	Air temperature May average Tallinn	°C	daily	station	Männik et al. 2015; Estonian Weather Service, 2016a; Jaagus et al. 2017
Atmosphere	230	Air temperature May average Tartu	°C	daily	station	Männik et al. 2015; Estonian Weather Service, 2016a; Jaagus et al. 2017
Atmosphere	231	Air temperature May average Võru	°C	daily	station	Männik et al. 2015; Estonian Weather Service, 2016a; Jaagus et al. 2017
Atmosphere	232	Air temperature May average Türi	°C	daily	station	Männik et al. 2015; Estonian Weather Service, 2016a; Jaagus et al. 2017
Atmosphere	233	Air temperature May average Vilsandi	°C	daily	station	Männik et al. 2015; Estonian Weather Service, 2016a; Jaagus et al. 2017
Atmosphere	234	Air temperature June average Tallinn	°C	daily	station	Männik et al. 2015; Estonian Weather Service, 2016a; Jaagus et al. 2017

Atmosphere	235	Air temperature June average Tartu	°C	daily	station	Männik et al. 2015; Estonian Weather Service, 2016a; Jaagus et al. 2017
Atmosphere	236	Air temperature June average Võru	°C	daily	station	Männik et al. 2015; Estonian Weather Service, 2016a; Jaagus et al. 2017
Atmosphere	237	Air temperature June average Türi	°C	daily	station	Männik et al. 2015; Estonian Weather Service, 2016a; Jaagus et al. 2017
Atmosphere	238	Air temperature June average Vilsandi	°C	daily	station	Männik et al. 2015; Estonian Weather Service, 2016a; Jaagus et al. 2017
Atmosphere	239	Air temperature July average Tallinn	°C	daily	station	Männik et al. 2015; Estonian Weather Service, 2016a; Jaagus et al. 2017
Atmosphere	240	Air temperature July average Tartu	°C	daily	station	Männik et al. 2015; Estonian Weather Service, 2016a; Jaagus et al. 2017
Atmosphere	241	Air temperature July average Võru	°C	daily	station	Männik et al. 2015; Estonian Weather Service, 2016a; Jaagus et al. 2017
Atmosphere	242	Air temperature July average Türi	°C	daily	station	Männik et al. 2015; Estonian Weather Service, 2016a; Jaagus et al. 2017
Atmosphere	243	Air temperature July average Vilsandi	°C	daily	station	Männik et al. 2015; Estonian Weather Service, 2016a; Jaagus et al. 2017
Atmosphere	244	Air temperature August average Tallinn	°C	daily	station	Männik et al. 2015; Estonian Weather Service, 2016a; Jaagus et al. 2017
Atmosphere	245	Air temperature August average Tartu	°C	daily	station	Männik et al. 2015; Estonian Weather Service, 2016a; Jaagus et al. 2017
Atmosphere	246	Air temperature August average Võru	°C	daily	station	Männik et al. 2015; Estonian Weather Service, 2016a; Jaagus et al. 2017
Atmosphere	247	Air temperature August average Türi	°C	daily	station	Männik et al. 2015; Estonian Weather Service, 2016a; Jaagus et al. 2017
Atmosphere	248	Air temperature August average Vilsandi	°C	daily	station	Männik et al. 2015; Estonian Weather Service, 2016a; Jaagus et al. 2017
Atmosphere	249	Air temperature September average Tallinn	°C	daily	station	Männik et al. 2015; Estonian Weather Service, 2016a; Jaagus et al. 2017

Atmosphere	250	Air temperature September average Tartu	°C	daily	station	Männik et al. 2015; Estonian Weather Service, 2016a; Jaagus et al. 2017
Atmosphere	251	Air temperature September average Võru	°C	daily	station	Männik et al. 2015; Estonian Weather Service, 2016a; Jaagus et al. 2017
Atmosphere	252	Air temperature September average Türi	°C	daily	station	Männik et al. 2015; Estonian Weather Service, 2016a; Jaagus et al. 2017
Atmosphere	253	Air temperature September average Vilsandi	°C	daily	station	Männik et al. 2015; Estonian Weather Service, 2016a; Jaagus et al. 2017
Atmosphere	254	Air temperature October average Tallinn	°C	daily	station	Männik et al. 2015; Estonian Weather Service, 2016a; Jaagus et al. 2017
Atmosphere	255	Air temperature October average Tartu	°C	daily	station	Männik et al. 2015; Estonian Weather Service, 2016a; Jaagus et al. 2017
Atmosphere	256	Air temperature October average Võru	°C	daily	station	Männik et al. 2015; Estonian Weather Service, 2016a; Jaagus et al. 2017
Atmosphere	257	Air temperature October average Türi	°C	daily	station	Männik et al. 2015; Estonian Weather Service, 2016a; Jaagus et al. 2017
Atmosphere	258	Air temperature October average Vilsandi	°C	daily	station	Männik et al. 2015; Estonian Weather Service, 2016a; Jaagus et al. 2017
Atmosphere	259	Air temperature November average Tallinn	°C	daily	station	Männik et al. 2015; Estonian Weather Service, 2016a; Jaagus et al. 2017
Atmosphere	260	Air temperature November average Tartu	°C	daily	station	Männik et al. 2015; Estonian Weather Service, 2016a; Jaagus et al. 2017
Atmosphere	261	Air temperature November average Võru	°C	daily	station	Männik et al. 2015; Estonian Weather Service, 2016a; Jaagus et al. 2017
Atmosphere	262	Air temperature November average Türi	°C	daily	station	Männik et al. 2015; Estonian Weather Service, 2016a; Jaagus et al. 2017
Atmosphere	263	Air temperature November average Vilsandi	°C	daily	station	Männik et al. 2015; Estonian Weather Service, 2016a; Jaagus et al. 2017
Atmosphere	264	Air temperature December average Tallinn	°C	daily	station	Männik et al. 2015; Estonian Weather Service, 2016a; Jaagus et al. 2017

Atmosphere	265	Air temperature December average Tartu	°C	daily	station	Männik et al. 2015; Estonian Weather Service, 2016a; Jaagus et al. 2017
Atmosphere	266	Air temperature December average Võru	°C	daily	station	Männik et al. 2015; Estonian Weather Service, 2016a; Jaagus et al. 2017
Atmosphere	267	Air temperature December average Türi	°C	daily	station	Männik et al. 2015; Estonian Weather Service, 2016a; Jaagus et al. 2017
Atmosphere	268	Air temperature December average Vilsandi	°C	daily	station	Männik et al. 2015; Estonian Weather Service, 2016a; Jaagus et al. 2017
Atmosphere	269	Air temperature winter average Tallinn	°C	daily	station	Männik et al. 2015; Estonian Weather Service, 2016a; Jaagus et al. 2017
Atmosphere	270	Air temperature winter average Tartu	°C	daily	station	Männik et al. 2015; Estonian Weather Service, 2016a; Jaagus et al. 2017
Atmosphere	271	Air temperature winter average Võru	°C	daily	station	Männik et al. 2015; Estonian Weather Service, 2016a; Jaagus et al. 2017
Atmosphere	272	Air temperature winter average Türi	°C	daily	station	Männik et al. 2015; Estonian Weather Service, 2016a; Jaagus et al. 2017
Atmosphere	273	Air temperature winter average Vilsandi	°C	daily	station	Männik et al. 2015; Estonian Weather Service, 2016a; Jaagus et al. 2017
Atmosphere	274	Air temperature spring average Tallinn	°C	daily	station	Männik et al. 2015; Estonian Weather Service, 2016a; Jaagus et al. 2017
Atmosphere	275	Air temperature spring average Tartu	°C	daily	station	Männik et al. 2015; Estonian Weather Service, 2016a; Jaagus et al. 2017
Atmosphere	276	Air temperature spring average Võru	°C	daily	station	Männik et al. 2015; Estonian Weather Service, 2016a; Jaagus et al. 2017
Atmosphere	277	Air temperature spring average Türi	°C	daily	station	Männik et al. 2015; Estonian Weather Service, 2016a; Jaagus et al. 2017
Atmosphere	278	Air temperature spring average Vilsandi	°C	daily	station	Männik et al. 2015; Estonian Weather Service, 2016a; Jaagus et al. 2017
Atmosphere	279	Air temperature summer average Tallinn	°C	daily	station	Männik et al. 2015; Estonian Weather Service, 2016a; Jaagus et al. 2017

Atmosphere	280	Air temperature summer average Tartu	°C	daily	station	Männik et al. 2015; Estonian Weather Service, 2016a; Jaagus et al. 2017
Atmosphere	281	Air temperature summer average Võru	°C	daily	station	Männik et al. 2015; Estonian Weather Service, 2016a; Jaagus et al. 2017
Atmosphere	282	Air temperature summer average Türi	°C	daily	station	Männik et al. 2015; Estonian Weather Service, 2016a; Jaagus et al. 2017
Atmosphere	283	Air temperature summer average Vilsandi	°C	daily	station	Männik et al. 2015; Estonian Weather Service, 2016a; Jaagus et al. 2017
Atmosphere	284	Air temperature autumn average Tallinn	°C	daily	station	Männik et al. 2015; Estonian Weather Service, 2016a; Jaagus et al. 2017
Atmosphere	285	Air temperature autumn average Tartu	°C	daily	station	Männik et al. 2015; Estonian Weather Service, 2016a; Jaagus et al. 2017
Atmosphere	286	Air temperature autumn average Võru	°C	daily	station	Männik et al. 2015; Estonian Weather Service, 2016a; Jaagus et al. 2017
Atmosphere	287	Air temperature autumn average Türi	°C	daily	station	Männik et al. 2015; Estonian Weather Service, 2016a; Jaagus et al. 2017
Atmosphere	288	Air temperature autumn average Vilsandi	°C	daily	station	Männik et al. 2015; Estonian Weather Service, 2016a; Jaagus et al. 2017
Atmosphere	289	Air temperature yearly average Tallinn	°C	daily	station	Männik et al. 2015; Estonian Weather Service, 2016a; Jaagus et al. 2017
Atmosphere	290	Air temperature yearly average Tartu	°C	daily	station	Männik et al. 2015; Estonian Weather Service, 2016a; Jaagus et al. 2017
Atmosphere	291	Air temperature yearly average Võru	°C	daily	station	Männik et al. 2015; Estonian Weather Service, 2016a; Jaagus et al. 2017
Atmosphere	292	Air temperature yearly average Türi	°C	daily	station	Männik et al. 2015; Estonian Weather Service, 2016a; Jaagus et al. 2017
Atmosphere	293	Air temperature yearly average Vilsandi	°C	daily	station	Männik et al. 2015; Estonian Weather Service, 2016a; Jaagus et al. 2017
Bog abiotic	1	Water level January average Männikjärve well 322	cm from ground	daily	station	Kont et al. 2007; Estonian Weather Service, 2016a

Bog abiotic	2	Water level January average Männikjärve well 213	cm from ground	daily	station	Kont et al. 2007; Estonian Weather Service, 2016a
Bog abiotic	3	Water level February average Männikjärve well 322	cm from ground	daily	station	Kont et al. 2007; Estonian Weather Service, 2016a
Bog abiotic	4	Water level February average Männikjärve well 213	cm from ground	daily	station	Kont et al. 2007; Estonian Weather Service, 2016a
Bog abiotic	5	Water level March average Männikjärve well 322	cm from ground	daily	station	Kont et al. 2007; Estonian Weather Service, 2016a
Bog abiotic	6	Water level March average Männikjärve well 213	cm from ground	daily	station	Kont et al. 2007; Estonian Weather Service, 2016a
Bog abiotic	7	Water level April average Männikjärve well 322	cm from ground	daily	station	Kont et al. 2007; Estonian Weather Service, 2016a
Bog abiotic	8	Water level April average Männikjärve well 213	cm from ground	daily	station	Kont et al. 2007; Estonian Weather Service, 2016a
Bog abiotic	9	Water level May average Männikjärve well 322	cm from ground	daily	station	Kont et al. 2007; Estonian Weather Service, 2016a
Bog abiotic	10	Water level May average Männikjärve well 213	cm from ground	daily	station	Kont et al. 2007; Estonian Weather Service, 2016a
Bog abiotic	11	Water level June average Männikjärve well 322	cm from ground	daily	station	Kont et al. 2007; Estonian Weather Service, 2016a
Bog abiotic	12	Water level June average Männikjärve well 213	cm from ground	daily	station	Kont et al. 2007; Estonian Weather Service, 2016a
Bog abiotic	13	Water level July average Männikjärve well 322	cm from ground	daily	station	Kont et al. 2007; Estonian Weather Service, 2016a
Bog abiotic	14	Water level July average Männikjärve well 213	cm from ground	daily	station	Kont et al. 2007; Estonian Weather Service, 2016a
Bog abiotic	15	Water level August average Männikjärve well 322	cm from ground	daily	station	Kont et al. 2007; Estonian Weather Service, 2016a
Bog abiotic	16	Water level August average Männikjärve well 213	cm from ground	daily	station	Kont et al. 2007; Estonian Weather Service, 2016a

Bog abiotic	17	Water level September average Männikjärve well 322	cm from ground	daily	station	Kont et al. 2007; Estonian Weather Service, 2016a
Bog abiotic	18	Water level September average Männikjärve well 213	cm from ground	daily	station	Kont et al. 2007; Estonian Weather Service, 2016a
Bog abiotic	19	Water level October average Männikjärve well 322	cm from ground	daily	station	Kont et al. 2007; Estonian Weather Service, 2016a
Bog abiotic	20	Water level October average Männikjärve well 213	cm from ground	daily	station	Kont et al. 2007; Estonian Weather Service, 2016a
Bog abiotic	21	Water level November average Männikjärve well 322	cm from ground	daily	station	Kont et al. 2007; Estonian Weather Service, 2016a
Bog abiotic	22	Water level November average Männikjärve well 213	cm from ground	daily	station	Kont et al. 2007; Estonian Weather Service, 2016a
Bog abiotic	23	Water level December average Männikjärve well 322	cm from ground	daily	station	Kont et al. 2007; Estonian Weather Service, 2016a
Bog abiotic	24	Water level December average Männikjärve well 213	cm from ground	daily	station	Kont et al. 2007; Estonian Weather Service, 2016a
Bog abiotic	25	Water level winter average Männikjärve well 322	cm from ground	daily	station	Kont et al. 2007; Estonian Weather Service, 2016a
Bog abiotic	26	Water level winter average Männikjärve well 213	cm from ground	daily	station	Kont et al. 2007; Estonian Weather Service, 2016a
Bog abiotic	27	Water level spring average Männikjärve well 322	cm from ground	daily	station	Kont et al. 2007; Estonian Weather Service, 2016a
Bog abiotic	28	Water level spring average Männikjärve well 213	cm from ground	daily	station	Kont et al. 2007; Estonian Weather Service, 2016a
Bog abiotic	29	Water level summer average Männikjärve well 322	cm from ground	daily	station	Kont et al. 2007; Estonian Weather Service, 2016a
Bog abiotic	30	Water level summer average Männikjärve well 213	cm from ground	daily	station	Kont et al. 2007; Estonian Weather Service, 2016a
Bog abiotic	31	Water level autumn average Männikjärve well 322	cm from ground	daily	station	Kont et al. 2007; Estonian Weather Service, 2016a

Bog abiotic	32	Water level autumn average Männikjärve well 213	cm from ground	daily	station	Kont et al. 2007; Estonian Weather Service, 2016a
Bog abiotic	33	Water level yearly average Männikjärve well 322	cm from ground	daily	station	Kont et al. 2007; Estonian Weather Service, 2016a
Bog abiotic	34	Water level yearly average Männikjärve well 213	cm from ground	daily	station	Kont et al. 2007; Estonian Weather Service, 2016a
Bog biotic	1	Radial growth increment of Scots pines Musa Bog yearly average	mm	yearly	whole bog	Paal et al. 2016
Bog biotic	2	Radial growth increment of Scots pines Esäkeste Bog yearly average	mm	yearly	whole bog	Paal et al. 2016
Bog biotic	3	Radial growth increment of Scots pines Allipa Bog yearly average	mm	yearly	whole bog	Paal et al. 2016
Bog biotic	4	Radial growth increment of Scots pines Koordi Bog yearly average	mm	yearly	whole bog	Paal et al. 2016
Bog biotic	5	Radial growth increment of Scots pines Hindaste Bog yearly average	mm	yearly	whole bog	Paal et al. 2016
Bog biotic	6	Radial growth increment of Scots pines Vedelsoo Bog yearly average	mm	yearly	whole bog	Paal et al. 2016
Bog biotic	7	Radial growth increment of Scots pines Laukasoo Bog yearly average	mm	yearly	whole bog	Paal et al. 2016
Bog biotic	8	Radial growth increment of Scots pines Umbusi Bog yearly average	mm	yearly	whole bog	Paal et al. 2016

Bog biotic	9	Radial growth increment of Scots pines Tellissaare Bog yearly average	mm	yearly	whole bog	Paal et al. 2016
Bog biotic	10	Radial growth increment of Scots pines Ullika Bog yearly average	mm	yearly	whole bog	Paal et al. 2016
Bog biotic	11	Radial growth increment of Scots pines Tuhu Bog yearly average	mm	yearly	whole bog	Paal et al. 2016
Bog biotic	12	Radial growth increment of Scots pines Maarjapeakse Bog yearly average	mm	yearly	whole bog	Paal et al. 2016
Bog biotic	13	Radial growth increment of Scots pines Keressaare Bog yearly average	mm	yearly	whole bog	Paal et al. 2016
Bog biotic	14	Radial growth increment of Scots pines Kauru Bog yearly average	mm	yearly	whole bog	Paal et al. 2016
River abiotic	1	Kasari River discharge January average	m^3 per s	daily	station	Estonian Weather Service, 2016b; Jaagus et al. 2017
River abiotic	2	Vihterpalu River discharge January average	m^3 per s	daily	station	Estonian Weather Service, 2016b; Jaagus et al. 2017
River abiotic	3	Oore River discharge January average	m^3 per s	daily	station	Estonian Weather Service, 2016b; Jaagus et al. 2018
River abiotic	4	Tõlliste River discharge January average	m^3 per s	daily	station	Estonian Weather Service, 2016b; Jaagus et al. 2019
River abiotic	5	Kääpa River discharge January average	m^3 per s	daily	station	Estonian Weather Service, 2016b; Jaagus et al. 2020
River abiotic	6	Kasari River discharge February average	m^3 per s	daily	station	Estonian Weather Service, 2016b

River abiotic	7	Vihterpalu River discharge February average	m³ per s	daily	station	Estonian Weather Service, 2016; Jaagus et al. 2017
River abiotic	8	Oore River discharge February average	m³ per s	daily	station	Estonian Weather Service, 2016b; Jaagus et al. 2017
River abiotic	9	Tõlliste River discharge February average	m³ per s	daily	station	Estonian Weather Service, 2016b; Jaagus et al. 2018
River abiotic	10	Kääpa River discharge February average	m³ per s	daily	station	Estonian Weather Service, 2016b; Jaagus et al. 2019
River abiotic	11	Kasari River discharge March average	m³ per s	daily	station	Estonian Weather Service, 2016b
River abiotic	12	Vihterpalu River discharge March average	m³ per s	daily	station	Estonian Weather Service, 2016b; Jaagus et al. 2017
River abiotic	13	Oore River discharge March average	m³ per s	daily	station	Estonian Weather Service, 2016b; Jaagus et al. 2018
River abiotic	14	Tõlliste River discharge March average	m³ per s	daily	station	Estonian Weather Service, 2016b; Jaagus et al. 2019
River abiotic	15	Kääpa River discharge March average	m³ per s	daily	station	Estonian Weather Service, 2016b; Jaagus et al. 2020
River abiotic	16	Kasari River discharge April average	m³ per s	daily	station	Estonian Weather Service, 2016b
River abiotic	17	Vihterpalu River discharge April average	m³ per s	daily	station	Estonian Weather Service, 2016b; Jaagus et al. 2017
River abiotic	18	Oore River discharge April average	m³ per s	daily	station	Estonian Weather Service, 2016b; Jaagus et al. 2018
River abiotic	19	Tõlliste River discharge April average	m³ per s	daily	station	Estonian Weather Service, 2016b; Jaagus et al. 2019
River abiotic	20	Kääpa River discharge April average	m³ per s	daily	station	Estonian Weather Service, 2016b; Jaagus et al. 2020
River abiotic	21	Kasari River discharge May average	m³ per s	daily	station	Estonian Weather Service, 2016b

River abiotic	22	Vihterpalu River discharge May average	m³ per s	daily	station	Estonian Weather Service, 2016b; Jaagus et al. 2017
River abiotic	23	Oore River discharge May average	m³ per s	daily	station	Estonian Weather Service, 2016b; Jaagus et al. 2018
River abiotic	24	Tõlliste River discharge May average	m³ per s	daily	station	Estonian Weather Service, 2016b; Jaagus et al. 2019
River abiotic	25	Kääpa River discharge May average	m³ per s	daily	station	Estonian Weather Service, 2016b; Jaagus et al. 2020
River abiotic	26	Kasari River discharge June average	m³ per s	daily	station	Estonian Weather Service, 2016b
River abiotic	27	Vihterpalu River discharge June average	m³ per s	daily	station	Estonian Weather Service, 2016b; Jaagus et al. 2017
River abiotic	28	Oore River discharge June average	m³ per s	daily	station	Estonian Weather Service, 2016b; Jaagus et al. 2018
River abiotic	29	Tõlliste River discharge June average	m³ per s	daily	station	Estonian Weather Service, 2016b; Jaagus et al. 2019
River abiotic	30	Kääpa River discharge June average	m³ per s	daily	station	Estonian Weather Service, 2016b; Jaagus et al. 2020
River abiotic	31	Kasari River discharge July average	m³ per s	daily	station	Estonian Weather Service, 2016b
River abiotic	32	Vihterpalu River discharge July average	m³ per s	daily	station	Estonian Weather Service, 2016b; Jaagus et al. 2017
River abiotic	33	Oore River discharge July average	m³ per s	daily	station	Estonian Weather Service, 2016b; Jaagus et al. 2018
River abiotic	34	Tõlliste River discharge July average	m³ per s	daily	station	Estonian Weather Service, 2016b; Jaagus et al. 2019
River abiotic	35	Kääpa River discharge July average	m³ per s	daily	station	Estonian Weather Service, 2016b; Jaagus et al. 2020
River abiotic	36	Kasari River discharge August average	m³ per s	daily	station	Estonian Weather Service, 2016b

River abiotic	37	Vihterpalu River discharge August average	m³ per s	daily	station	Estonian Weather Service, 2016b; Jaagus et al. 2017
River abiotic	38	Oore River discharge August average	m³ per s	daily	station	Estonian Weather Service, 2016b; Jaagus et al. 2018
River abiotic	39	Tõlliste River discharge August average	m³ per s	daily	station	Estonian Weather Service, 2016b; Jaagus et al. 2019
River abiotic	40	Kääpa River discharge August average	m³ per s	daily	station	Estonian Weather Service, 2016b; Jaagus et al. 2020
River abiotic	41	Kasari River discharge September average	m³ per s	daily	station	Estonian Weather Service, 2016b
River abiotic	42	Vihterpalu River discharge September average	m³ per s	daily	station	Estonian Weather Service, 2016b; Jaagus et al. 2017
River abiotic	43	Oore River discharge September average	m³ per s	daily	station	Estonian Weather Service, 2016b; Jaagus et al. 2018
River abiotic	44	Tõlliste River discharge September average	m³ per s	daily	station	Estonian Weather Service, 2016b; Jaagus et al. 2019
River abiotic	45	Kääpa River discharge September average	m³ per s	daily	station	Estonian Weather Service, 2016b; Jaagus et al. 2020
River abiotic	46	Kasari River discharge October average	m³ per s	daily	station	Estonian Weather Service, 2016b
River abiotic	47	Vihterpalu River discharge October average	m³ per s	daily	station	Estonian Weather Service, 2016b; Jaagus et al. 2017
River abiotic	48	Oore River discharge October average	m³ per s	daily	station	Estonian Weather Service, 2016b; Jaagus et al. 2018
River abiotic	49	Tõlliste River discharge October average	m³ per s	daily	station	Estonian Weather Service, 2016b; Jaagus et al. 2019
River abiotic	50	Kääpa River discharge October average	m³ per s	daily	station	Estonian Weather Service, 2016b; Jaagus et al. 2020
River abiotic	51	Kasari River discharge November average	m³ per s	daily	station	Estonian Weather Service, 2016b

River abiotic	52	Vihterpalu River discharge November average	m³ per s	daily	station	Estonian Weather Service, 2016b; Jaagus et al. 2017
River abiotic	53	Oore River discharge November average	m³ per s	daily	station	Estonian Weather Service, 2016b; Jaagus et al. 2018
River abiotic	54	Tõlliste River discharge November average	m³ per s	daily	station	Estonian Weather Service, 2016b; Jaagus et al. 2019
River abiotic	55	Kääpa River discharge November average	m³ per s	daily	station	Estonian Weather Service, 2016b; Jaagus et al. 2020
River abiotic	56	Kasari River discharge December average	m³ per s	daily	station	Estonian Weather Service, 2016b
River abiotic	57	Vihterpalu River discharge December average	m³ per s	daily	station	Estonian Weather Service, 2016b; Jaagus et al. 2017
River abiotic	58	Oore River discharge December average	m³ per s	daily	station	Estonian Weather Service, 2016b; Jaagus et al. 2017
River abiotic	59	Tõlliste River discharge December average	m³ per s	daily	station	Estonian Weather Service, 2016b; Jaagus et al. 2018
River abiotic	60	Kääpa River discharge December average	m³ per s	daily	station	Estonian Weather Service, 2016b; Jaagus et al. 2019
River abiotic	61	Kasari River discharge winter average	m³ per s	daily	station	Estonian Weather Service, 2016b
River abiotic	62	Vihterpalu River discharge winter average	m³ per s	daily	station	Estonian Weather Service, 2016b; Jaagus et al. 2017
River abiotic	63	Oore River discharge winter average	m³ per s	daily	station	Estonian Weather Service, 2016b; Jaagus et al. 2018
River abiotic	64	Tõlliste River discharge winter average	m³ per s	daily	station	Estonian Weather Service, 2016b; Jaagus et al. 2019
River abiotic	65	Kääpa River discharge winter average	m³ per s	daily	station	Estonian Weather Service, 2016b; Jaagus et al. 2020
River abiotic	66	Kasari River discharge spring average	m³ per s	daily	station	Estonian Weather Service, 2016b

River abiotic	67	Vihterpalu River discharge spring average	m³ per s	daily	station	Estonian Weather Service, 2016b; Jaagus et al. 2017
River abiotic	68	Oore River discharge spring average	m³ per s	daily	station	Estonian Weather Service, 2016b; Jaagus et al. 2018
River abiotic	69	Tõlliste River discharge spring average	m³ per s	daily	station	Estonian Weather Service, 2016b; Jaagus et al. 2019
River abiotic	70	Kääpa River discharge spring average	m³ per s	daily	station	Estonian Weather Service, 2016b; Jaagus et al. 2020
River abiotic	71	Kasari River discharge summer average	m³ per s	daily	station	Estonian Weather Service, 2016b
River abiotic	72	Vihterpalu River discharge summer average	m³ per s	daily	station	Estonian Weather Service, 2016b; Jaagus et al. 2017
River abiotic	73	Oore River discharge summer average	m³ per s	daily	station	Estonian Weather Service, 2016b; Jaagus et al. 2018
River abiotic	74	Tõlliste River discharge summer average	m³ per s	daily	station	Estonian Weather Service, 2016b; Jaagus et al. 2019
River abiotic	75	Kääpa River discharge summer average	m³ per s	daily	station	Estonian Weather Service, 2016b; Jaagus et al. 2020
River abiotic	76	Kasari River discharge autumn average	m³ per s	daily	station	Estonian Weather Service, 2016b
River abiotic	77	Vihterpalu River discharge autumn average	m³ per s	daily	station	Estonian Weather Service, 2016b; Jaagus et al. 2017
River abiotic	78	Oore River discharge autumn average	m³ per s	daily	station	Estonian Weather Service, 2016; Jaagus et al. 2017
River abiotic	79	Tõlliste River discharge autumn average	m³ per s	daily	station	Estonian Weather Service, 2016; Jaagus et al. 2018
River abiotic	80	Kääpa River discharge autumn average	m³ per s	daily	station	Estonian Weather Service, 2016; Jaagus et al. 2019
River abiotic	81	Kasari River discharge yearly average	m³ per s	daily	station	Estonian Weather Service, 2016b

River abiotic	82	Vihterpalu River discharge yearly average	m^3 per s	daily	station	Estonian Weather Service, 2016b; Jaagus et al. 2017
River abiotic	83	Oore River discharge yearly average	m^3 per s	daily	station	Estonian Weather Service, 2016b; Jaagus et al. 2018
River abiotic	84	Tõlliste River discharge yearly average	m^3 per s	daily	station	Estonian Weather Service, 2016b; Jaagus et al. 2019
River abiotic	85	Kääpa River discharge yearly average	m^3 per s	daily	station	Estonian Weather Service, 2016b; Jaagus et al. 2020
Lake abiotic	1	Water temperature January average Võrtsjärv Lake	°C	daily	station	original data; Nõges and Järvet, 2005; Nõges and Nõges, 2014
Lake abiotic	2	Water temperature February average Võrtsjärv Lake	°C	daily	station	original data; Nõges and Järvet, 2005; Nõges and Nõges, 2014
Lake abiotic	3	Water temperature March average Võrtsjärv Lake	°C	daily	station	original data; Nõges and Järvet, 2005; Nõges and Nõges, 2014
Lake abiotic	4	Water temperature April average Võrtsjärv Lake	°C	daily	station	original data; Nõges and Järvet, 2005; Nõges and Nõges, 2014
Lake abiotic	5	Water temperature May average Võrtsjärv Lake	°C	daily	station	original data; Nõges and Järvet, 2005; Nõges and Nõges, 2014
Lake abiotic	6	Water temperature June average Võrtsjärv Lake	°C	daily	station	original data; Nõges and Järvet, 2005; Nõges and Nõges, 2014
Lake abiotic	7	Water temperature July average Võrtsjärv Lake	°C	daily	station	original data; Nõges and Järvet, 2005; Nõges and Nõges, 2014
Lake abiotic	8	Water temperature August average Võrtsjärv Lake	°C	daily	station	original data; Nõges and Järvet, 2005; Nõges and Nõges, 2014
Lake abiotic	9	Water temperature September average Võrtsjärv Lake	°C	daily	station	original data; Nõges and Järvet, 2005; Nõges and Nõges, 2014
Lake abiotic	10	Water temperature October average Võrtsjärv Lake	°C	daily	station	original data; Nõges and Järvet, 2005; Nõges and Nõges, 2014
Lake abiotic	11	Water temperature November average Võrtsjärv Lake	°C	daily	station	original data; Nõges and Järvet, 2005; Nõges and Nõges, 2014

Lake abiotic	12	Water temperature December average Võrtsjärv Lake	°C	daily	station	original data; Nõges and Järvet, 2005; Nõges and Nõges, 2014
Lake abiotic	13	Water temperature winter average Võrtsjärv Lake	°C	daily	station	original data; Nõges and Järvet, 2005; Nõges and Nõges, 2014
Lake abiotic	14	Water temperature spring average Võrtsjärv Lake	°C	daily	station	original data; Nõges and Järvet, 2005; Nõges and Nõges, 2014
Lake abiotic	15	Water temperature summer average Võrtsjärv Lake	°C	daily	station	original data; Nõges and Järvet, 2005; Nõges and Nõges, 2014
Lake abiotic	16	Water temperature autumn average Võrtsjärv Lake	°C	daily	station	original data; Nõges and Järvet, 2005; Nõges and Nõges, 2014
Lake abiotic	17	Water temperature yearly average Võrtsjärv Lake	°C	daily	station	original data; Nõges and Järvet, 2005; Nõges and Nõges, 2014
Lake abiotic	18	Water level January average Võrtsjärv Lake	m above the sea	daily	station	Estonian Weather Service, 2016a; Järvet, 2004; Heinsalu et al. 2007
Lake abiotic	19	Water level February average Võrtsjärv Lake	m above the sea	daily	station	Estonian Weather Service, 2016a; Järvet, 2004; Heinsalu et al. 2007
Lake abiotic	20	Water level March average Võrtsjärv Lake	m above the sea	daily	station	Estonian Weather Service, 2016a; Järvet, 2004; Heinsalu et al. 2007
Lake abiotic	21	Water level April average Võrtsjärv Lake	m above the sea	daily	station	Estonian Weather Service, 2016a; Järvet, 2004; Heinsalu et al. 2007
Lake abiotic	22	Water level May average Võrtsjärv Lake	m above the sea	daily	station	Estonian Weather Service, 2016a; Järvet, 2004; Heinsalu et al. 2007
Lake abiotic	23	Water level June average Võrtsjärv Lake	m above the sea	daily	station	Estonian Weather Service, 2016a; Järvet, 2004; Heinsalu et al. 2007
Lake abiotic	24	Water level July average Võrtsjärv Lake	m above the sea	daily	station	Estonian Weather Service, 2016a; Järvet, 2004; Heinsalu et al. 2007
Lake abiotic	25	Water level August average Võrtsjärv Lake	m above the sea	daily	station	Estonian Weather Service, 2016a; Järvet, 2004; Heinsalu et al. 2007
Lake abiotic	26	Water level September average Võrtsjärv Lake	m above the sea	daily	station	Estonian Weather Service, 2016a; Järvet, 2004; Heinsalu et al. 2007

Lake abiotic	27	Water level October average Võrtsjärv Lake	m above the sea	daily	station	Estonian Weather Service, 2016a; Järvet, 2004; Heinsalu et al. 2007
Lake abiotic	28	Water level November average Võrtsjärv Lake	m above the sea	daily	station	Estonian Weather Service, 2016a; Järvet, 2004; Heinsalu et al. 2007
Lake abiotic	29	Water level December average Võrtsjärv Lake	m above the sea	daily	station	Estonian Weather Service, 2016a; Järvet, 2004; Heinsalu et al. 2007
Lake abiotic	30	Water level winter average Võrtsjärv Lake	m above the sea	daily	station	Estonian Weather Service, 2016a; Järvet, 2004; Heinsalu et al. 2007
Lake abiotic	31	Water level spring average Võrtsjärv Lake	m above the sea	daily	station	Estonian Weather Service, 2016a; Järvet, 2004; Heinsalu et al. 2007
Lake abiotic	32	Water level summer average Võrtsjärv Lake	m above the sea	daily	station	Estonian Weather Service, 2016a; Järvet, 2004; Heinsalu et al. 2007
Lake abiotic	33	Water level autumn average Võrtsjärv Lake	m above the sea	daily	station	Estonian Weather Service, 2016a; Järvet, 2004; Heinsalu et al. 2007
Lake abiotic	34	Water level yearly average Võrtsjärv Lake	m above the sea	daily	station	Estonian Weather Service, 2016a; Järvet, 2004; Heinsalu et al. 2007
Lake biotic	1	Total invertebrate abundance winter average Võrtsjärv Lake	ind. per m^2	monthly	station	original data; Kangur et al. 2004; Kumari et al. 2007
Lake biotic	2	Chironomidae abundance winter average Võrtsjärv Lake	ind. per m^2	monthly	station	original data; Kangur et al. 2004; Kumari et al. 2007
Lake biotic	3	Oligochaeta abundance winter average Võrtsjärv Lake	ind. per m^2	monthly	station	original data; Kangur et al. 2004; Kumari et al. 2007
Lake biotic	4	Mollusca abundance winter average Võrtsjärv Lake	ind. per m^2	monthly	station	original data; Kangur et al. 2004; Kumari et al. 2007
Lake biotic	5	Other invertebrate abundance winter average Võrtsjärv Lake	ind. per m^2	monthly	station	original data; Kangur et al. 2004; Kumari et al. 2007
Lake biotic	6	Total invertebrate biomass winter average Võrtsjärv Lake	g per m^2	monthly	station	original data; Kangur et al. 2004; Kumari et al. 2007
Lake biotic	7	Chironomidae biomass winter average Võrtsjärv Lake	g per m^2	monthly	station	original data; Kangur et al. 2004; Kumari et al. 2007

Lake biotic	8	Oligochaeta biomass winter average Võrtsjärv Lake	g per m²	monthly	station	original data; Kangur et al. 2004; Kumari et al. 2007
Lake biotic	9	Mollusca biomass winter average Võrtsjärv Lake	g per m²	monthly	station	original data; Kangur et al. 2004; Kumari et al. 2007
Lake biotic	10	Other invertebrate biomass winter average Võrtsjärv Lake	g per m²	monthly	station	original data; Kangur et al. 2004; Kumari et al. 2007
Lake biotic	11	Total invertebrate abundance spring average Võrtsjärv Lake	ind. per m²	monthly	station	original data; Kangur et al. 2004; Kumari et al. 2007
Lake biotic	12	Chironomidae abundance spring average Võrtsjärv Lake	ind. per m²	monthly	station	original data; Kangur et al. 2004; Kumari et al. 2007
Lake biotic	13	Oligochaeta abundance spring average Võrtsjärv Lake	ind. per m²	monthly	station	original data; Kangur et al. 2004; Kumari et al. 2007
Lake biotic	14	Mollusca abundance spring average Võrtsjärv Lake	ind. per m²	monthly	station	original data; Kangur et al. 2004; Kumari et al. 2007
Lake biotic	15	Other invertebrate abundance spring average Võrtsjärv Lake	ind. per m²	monthly	station	original data; Kangur et al. 2004; Kumari et al. 2007
Lake biotic	16	Total invertebrate biomass spring average Võrtsjärv Lake	g per m²	monthly	station	original data; Kangur et al. 2004; Kumari et al. 2007
Lake biotic	17	Chironomidae biomass spring average Võrtsjärv Lake	g per m²	monthly	station	original data; Kangur et al. 2004; Kumari et al. 2007
Lake biotic	18	Oligochaeta biomass spring average Võrtsjärv Lake	g per m²	monthly	station	original data; Kangur et al. 2004; Kumari et al. 2007
Lake biotic	19	Mollusca biomass spring average Võrtsjärv Lake	g per m²	monthly	station	original data; Kangur et al. 2004; Kumari et al. 2007
Lake biotic	20	Other invertebrate biomass spring average Võrtsjärv Lake	g per m²	monthly	station	original data; Kangur et al. 2004; Kumari et al. 2007
Lake biotic	21	Total invertebrate abundance summer average Võrtsjärv Lake	ind. per m²	monthly	station	original data; Kangur et al. 2004; Kumari et al. 2007

Lake biotic	22	Chironomidae abundance summer average Võrtsjärv Lake	ind. per m²	monthly	station	original data; Kangur et al. 2004; Kumari et al. 2007
Lake biotic	23	summer average Võrtsjärv Lake	ind. per m²	monthly	station	original data; Kangur et al. 2004; Kumari et al. 2007
Lake biotic	24	Mollusca abundance summer average Võrtsjärv Lake	ind. per m²	monthly	station	original data; Kangur et al. 2004; Kumari et al. 2007
Lake biotic	25	Other invertebrate abundance summer average Võrtsjärv Lake	ind. per m²	monthly	station	original data; Kangur et al. 2004; Kumari et al. 2007
Lake biotic	26	Total invertebrate biomass summer average Võrtsjärv Lake	g per m²	monthly	station	original data; Kangur et al. 2004; Kumari et al. 2007
Lake biotic	27	summer average Võrtsjärv Lake	g per m²	monthly	station	original data; Kangur et al. 2004; Kumari et al. 2007
Lake biotic	28	Oligochaeta biomass summer average Võrtsjärv Lake	g per m²	monthly	station	original data; Kangur et al. 2004; Kumari et al. 2007
Lake biotic	29	Mollusca biomass summer average Võrtsjärv Lake	g per m²	monthly	station	original data; Kangur et al. 2004; Kumari et al. 2007
Lake biotic	30	Other invertebrate biomass summer average Võrtsjärv Lake	g per m²	monthly	station	original data; Kangur et al. 2004; Kumari et al. 2007
Lake biotic	31	autumn average Võrtsjärv Lake	ind. per m²	monthly	station	original data; Kangur et al. 2004; Kumari et al. 2007
Lake biotic	32	autumn average Võrtsjärv Lake	ind. per m²	monthly	station	original data; Kangur et al. 2004; Kumari et al. 2007
Lake biotic	33	autumn average Võrtsjärv Lake	ind. per m²	monthly	station	original data; Kangur et al. 2004; Kumari et al. 2007
Lake biotic	34	Mollusca abundance autumn average Võrtsjärv Lake	ind. per m²	monthly	station	original data; Kangur et al. 2004; Kumari et al. 2007

Lake biotic	35	autumn average Võrtsjärv Lake	ind. per m²	monthly	station	original data; Kangur et al. 2004; Kumari et al. 2007
Lake biotic	36	autumn average Võrtsjärv Lake	g per m²	monthly	station	original data; Kangur et al. 2004; Kumari et al. 2007
Lake biotic	37	autumn average Võrtsjärv Lake	g per m²	monthly	station	original data; Kangur et al. 2004; Kumari et al. 2007
Lake biotic	38	Oligochaeta biomass autumn average Võrtsjärv Lake	g per m²	monthly	station	original data; Kangur et al. 2004; Kumari et al. 2007
Lake biotic	39	Mollusca biomass autumn average Võrtsjärv Lake	g per m²	monthly	station	original data; Kangur et al. 2004; Kumari et al. 2007
Lake biotic	40	autumn average Võrtsjärv Lake	g per m²	monthly	station	original data; Kangur et al. 2004; Kumari et al. 2007
Lake biotic	41	Chironomidae abundance yearly average Peipsi Lake	ind. per m²	yearly	station	original data; Timm et al. 2001; Kumari et al. 2007
Lake biotic	42	Oligochaeta abundance yearly average Peipsi Lake	ind. per m²	yearly	station	original data; Timm et al. 2001; Kumari et al. 2007
Lake biotic	43	Mollusca abundance yearly average Peipsi Lake	ind. per m²	yearly	station	original data; Timm et al. 2001; Kumari et al. 2007
Lake biotic	44	Other invertebrate abundance yearly average Peipsi Lake	ind. per m²	yearly	station	original data; Timm et al. 2001; Kumari et al. 2007
Lake biotic	45	Total invertebrate abundance yearly average Peipsi Lake	ind. per m²	yearly	station	original data; Timm et al. 2001; Kumari et al. 2007
Lake biotic	46	Chironomidae biomass yearly average Peipsi Lake	g per m²	yearly	station	original data; Timm et al. 2001; Kumari et al. 2007
Lake biotic	47	Oligochaeta biomass yearly average Peipsi Lake	g per m²	yearly	station	original data; Timm et al. 2001; Kumari et al. 2007
Lake biotic	48	average Peipsi Lake	g per m²	yearly	station	et al. 2007
Lake biotic	49	Other invertebrate biomass yearly average Peipsi Lake	g per m²	yearly	station	original data; Timm et al. 2001; Kumari et al. 2007
Lake biotic	50	Total invertebrate biomass yearly average Peipsi Lake	g per m²	yearly	station	original data; Timm et al. 2001; Kumari et al. 2007

Lake biotic	51	Chlorophyll concentration summer peak Võrtsjärv Lake	µg per L	monthly	station	original data; Nõges et al. 2010
Lake biotic	52	Chlorophyll concentration summer average Võrtsjärv Lake	mm³ per L	monthly	station	original data; Nõges et al. 2010
Lake biotic	53	Cyanobacteria abundance summer peak Võrtsjärv Lake	µg per L	monthly	station	original data; Nõges et al. 2010
Lake biotic	54	Cyanobacteria abundance summer average Võrtsjärv Lake	mm³ per L	monthly	station	original data; Nõges et al. 2010
Lake biotic	55	Anabeana abundance summer average Võrtsjärv Lake	mm³ per L	monthly	station	original data; Nõges et al. 2010
Lake biotic	56	Aphanizomenon abundance summer average Võrtsjärv Lake	mm³ per L	monthly	station	original data; Nõges et al. 2010
Lake biotic	57	Merismopaedia abundance summer average Võrtsjärv Lake	mm³ per L	monthly	station	original data; Nõges et al. 2010
Lake biotic	58	Snowella abundance summer average Võrtsjärv Lake	mm³ per L	monthly	station	original data; Nõges et al. 2010
Lake biotic	59	Woronichinia abundance summer average Võrtsjärv Lake	mm³ per L	monthly	station	original data; Nõges et al. 2010
Lake biotic	60	summer average Võrtsjärv Lake	mm³ per L	monthly	station	original data; Nõges et al. 2010
Lake biotic	61	summer average Võrtsjärv Lake	mm³ per L	monthly	station	original data; Nõges et al. 2010
Lake biotic	62	summer average Võrtsjärv Lake	mm³ per L	monthly	station	original data; Nõges et al. 2010

Lake biotic	63	Planktolyngbya abundance summer average Võrtsjärv Lake	mm^3 per L	monthly	station	original data; Nõges et al. 2010
Lake biotic	64	Zooplankton biomass May-October average Võrtsjärv Lake	mg per L	monthly	station	original data; Agasild et al. 2007; Zingel and Haberman, 2007
Lake biotic	65	Cladocera biomass May-October average Võrtsjärv Lake	mg per L	monthly	station	original data; Agasild et al. 2007; Zingel and Haberman, 2007
Lake biotic	66	Copepoda biomass May-October average Võrtsjärv Lake	mg per L	monthly	station	original data; Agasild et al. 2007; Zingel and Haberman, 2007
Lake biotic	67	Rotatoria biomass May-October average Võrtsjärv Lake	mg per L	monthly	station	original data; Agasild et al. 2007; Zingel and Haberman, 2007
Sea abiotic	1	Coastal sea ice surface area yearly average	km^2	daily	Baltic Sea	Finnish Meteorological Institute
Sea abiotic	2	Water level January average Narva Jõesuu	cm	daily	station	Suursaar et al. 2015
Sea abiotic	3	Water level January average Pärnu Bay	cm	daily	station	Suursaar et al. 2015
Sea abiotic	4	Water level February average Narva Jõesuu	cm	daily	station	Suursaar et al. 2015
Sea abiotic	5	Water level February average Pärnu Bay	cm	daily	station	Suursaar et al. 2015
Sea abiotic	6	Water level March average Narva Jõesuu	cm	daily	station	Suursaar et al. 2015
Sea abiotic	7	Water level March average Pärnu Bay	cm	daily	station	Suursaar et al. 2015
Sea abiotic	8	Water level April average Narva Jõesuu	cm	daily	station	Suursaar et al. 2015

Sea abiotic	9	Water level April average Pärnu Bay	cm	daily	station	Suursaar et al. 2015
Sea abiotic	10	Water level May average Narva Jõesuu	cm	daily	station	Suursaar et al. 2015
Sea abiotic	11	Water level May average Pärnu Bay	cm	daily	station	Suursaar et al. 2015
Sea abiotic	12	Water level June average Narva Jõesuu	cm	daily	station	Suursaar et al. 2015
Sea abiotic	13	Water level June average Pärnu Bay	cm	daily	station	Suursaar et al. 2015
Sea abiotic	14	Water level July average Narva Jõesuu	cm	daily	station	Suursaar et al. 2015
Sea abiotic	15	Water level July average Pärnu Bay	cm	daily	station	Suursaar et al. 2015
Sea abiotic	16	Water level August average Narva Jõesuu	cm	daily	station	Suursaar et al. 2015
Sea abiotic	17	Water level August average Pärnu Bay	cm	daily	station	Suursaar et al. 2015
Sea abiotic	18	average Narva Jõesuu	cm	daily	station	Suursaar et al. 2015
Sea abiotic	19	Water level September average Pärnu Bay	cm	daily	station	Suursaar et al. 2015
Sea abiotic	20	Water level October average Narva Jõesuu	cm	daily	station	Suursaar et al. 2015
Sea abiotic	21	Water level October average Pärnu Bay	cm	daily	station	Suursaar et al. 2015
Sea abiotic	22	average Narva Jõesuu	cm	daily	station	Suursaar et al. 2015
Sea abiotic	23	Water level November average Pärnu Bay	cm	daily	station	Suursaar et al. 2015
Sea abiotic	24	Narva Jõesuu	cm	daily	station	Suursaar et al. 2015
Sea abiotic	25	Water level December average Pärnu Bay	cm	daily	station	Suursaar et al. 2015

Sea abiotic	26	Water level winter average Narva Jõesuu	cm	daily	station	Suursaar et al. 2015
Sea abiotic	27	Water level winter average Pärnu Bay	cm	daily	station	Suursaar et al. 2015
Sea abiotic	28	Water level spring average Narva Jõesuu	cm	daily	station	Suursaar et al. 2015
Sea abiotic	29	Water level spring average Pärnu Bay	cm	daily	station	Suursaar et al. 2015
Sea abiotic	30	Water level summer average Narva Jõesuu	cm	daily	station	Suursaar et al. 2015
Sea abiotic	31	Water level summer average Pärnu Bay	cm	daily	station	Suursaar et al. 2015
Sea abiotic	32	Water level autumn average Narva Jõesuu	cm	daily	station	Suursaar et al. 2015
Sea abiotic	33	Water level autumn average Pärnu Bay	cm	daily	station	Suursaar et al. 2015
Sea abiotic	34	Water level yearly average Narva Jõesuu	cm	daily	station	Suursaar et al. 2015
Sea abiotic	35	Water level yearly average Pärnu Bay	cm	daily	station	Suursaar et al. 2015
Sea abiotic	36	Salinity surface layer January average Gulf of Finland	unitless dimension	hourly	Gulf of Finland	original data; Maljutenko and Raudsepp, 2014
Sea abiotic	37	Salinity bottom layer January average Gulf of Finland	unitless dimension	hourly	Gulf of Finland	original data; Maljutenko and Raudsepp, 2014
Sea abiotic	38	Salinity surface layer January average Gulf of Riga	unitless dimension	hourly	Gulf of Riga	original data; Maljutenko and Raudsepp, 2014
Sea abiotic	39	Salinity bottom layer January average Gulf of Riga	unitless dimension	hourly	Gulf of Riga	original data; Maljutenko and Raudsepp, 2014
Sea abiotic	40	Salinity surface layer January average Baltic Proper	unitless dimension	hourly	Baltic Proper	original data; Maljutenko and Raudsepp, 2014

Sea abiotic	41	Salinity bottom layer January average Baltic Proper	unitless dimension	hourly	Baltic Proper	original data; Maljutenko and Raudsepp, 2014
Sea abiotic	42	Salinity surface layer February average Gulf of Finland	unitless dimension	hourly	Gulf of Finland	original data; Maljutenko and Raudsepp, 2014
Sea abiotic	43	Salinity bottom layer February average Gulf of Finland	unitless dimension	hourly	Gulf of Finland	original data; Maljutenko and Raudsepp, 2014
Sea abiotic	44	Salinity surface layer February average Gulf of Riga	unitless dimension	hourly	Gulf of Riga	original data; Maljutenko and Raudsepp, 2014
Sea abiotic	45	Salinity bottom layer February average Gulf of Riga	unitless dimension	hourly	Gulf of Riga	original data; Maljutenko and Raudsepp, 2014
Sea abiotic	46	Salinity surface layer February average Baltic Proper	unitless dimension	hourly	Baltic Proper	original data; Maljutenko and Raudsepp, 2014
Sea abiotic	47	Salinity bottom layer February average Baltic Proper	unitless dimension	hourly	Baltic Proper	original data; Maljutenko and Raudsepp, 2014
Sea abiotic	48	Salinity surface layer March average Gulf of Finland	unitless dimension	hourly	Gulf of Finland	original data; Maljutenko and Raudsepp, 2014
Sea abiotic	49	Salinity bottom layer March average Gulf of Finland	unitless dimension	hourly	Gulf of Finland	original data; Maljutenko and Raudsepp, 2014
Sea abiotic	50	Salinity surface layer March average Gulf of Riga	unitless dimension	hourly	Gulf of Riga	original data; Maljutenko and Raudsepp, 2014
Sea abiotic	51	Salinity bottom layer March average Gulf of Riga	unitless dimension	hourly	Gulf of Riga	original data; Maljutenko and Raudsepp, 2014
Sea abiotic	52	Salinity surface layer March average Baltic Proper	unitless dimension	hourly	Baltic Proper	original data; Maljutenko and Raudsepp, 2014
Sea abiotic	53	Salinity bottom layer March average Baltic Proper	unitless dimension	hourly	Baltic Proper	original data; Maljutenko and Raudsepp, 2014
Sea abiotic	54	Salinity surface layer April average Gulf of Finland	unitless dimension	hourly	Gulf of Finland	original data; Maljutenko and Raudsepp, 2014
Sea abiotic	55	Salinity bottom layer April average Gulf of Finland	unitless dimension	hourly	Gulf of Finland	original data; Maljutenko and Raudsepp, 2014

Sea abiotic	56	Salinity surface layer April average Gulf of Riga	unitless dimension	hourly	Gulf of Riga	original data; Maljutenko and Raudsepp, 2014
Sea abiotic	57	Salinity bottom layer April average Gulf of Riga	unitless dimension	hourly	Gulf of Riga	original data; Maljutenko and Raudsepp, 2014
Sea abiotic	58	Salinity surface layer April average Baltic Proper	unitless dimension	hourly	Baltic Proper	original data; Maljutenko and Raudsepp, 2014
Sea abiotic	59	Salinity bottom layer April average Baltic Proper	unitless dimension	hourly	Baltic Proper	original data; Maljutenko and Raudsepp, 2014
Sea abiotic	60	Salinity surface layer May average Gulf of Finland	unitless dimension	hourly	Gulf of Finland	original data; Maljutenko and Raudsepp, 2014
Sea abiotic	61	Salinity bottom layer May average Gulf of Finland	unitless dimension	hourly	Gulf of Finland	original data; Maljutenko and Raudsepp, 2014
Sea abiotic	62	Salinity surface layer May average Gulf of Riga	unitless dimension	hourly	Gulf of Riga	original data; Maljutenko and Raudsepp, 2014
Sea abiotic	63	Salinity bottom layer May average Gulf of Riga	unitless dimension	hourly	Gulf of Riga	original data; Maljutenko and Raudsepp, 2014
Sea abiotic	64	Salinity surface layer May average Baltic Proper	unitless dimension	hourly	Baltic Proper	original data; Maljutenko and Raudsepp, 2014
Sea abiotic	65	Salinity bottom layer May average Baltic Proper	unitless dimension	hourly	Baltic Proper	original data; Maljutenko and Raudsepp, 2014
Sea abiotic	66	Salinity surface layer June average Gulf of Finland	unitless dimension	hourly	Gulf of Finland	original data; Maljutenko and Raudsepp, 2014
Sea abiotic	67	Salinity bottom layer June average Gulf of Finland	unitless dimension	hourly	Gulf of Finland	original data; Maljutenko and Raudsepp, 2014
Sea abiotic	68	Salinity surface layer June average Gulf of Riga	unitless dimension	hourly	Gulf of Riga	original data; Maljutenko and Raudsepp, 2014
Sea abiotic	69	Salinity bottom layer June average Gulf of Riga	unitless dimension	hourly	Gulf of Riga	original data; Maljutenko and Raudsepp, 2014
Sea abiotic	70	Salinity surface layer June average Baltic Proper	unitless dimension	hourly	Baltic Proper	original data; Maljutenko and Raudsepp, 2014

Sea abiotic	71	Salinity bottom layer June average Baltic Proper	unitless dimension	hourly	Baltic Proper	original data; Maljutenko and Raudsepp, 2014
Sea abiotic	72	Salinity surface layer July average Gulf of Finland	unitless dimension	hourly	Gulf of Finland	original data; Maljutenko and Raudsepp, 2014
Sea abiotic	73	Salinity bottom layer July average Gulf of Finland	unitless dimension	hourly	Gulf of Finland	original data; Maljutenko and Raudsepp, 2014
Sea abiotic	74	average Gulf of Riga	dimension	hourly	Gulf of Riga	2014
Sea abiotic	75	average Gulf of Riga	dimension	hourly	Gulf of Riga	2014
Sea abiotic	76	Salinity surface layer July average Baltic Proper	unitless dimension	hourly	Baltic Proper	original data; Maljutenko and Raudsepp, 2014
Sea abiotic	77	Salinity bottom layer July average Baltic Proper	unitless dimension	hourly	Baltic Proper	original data; Maljutenko and Raudsepp, 2014
Sea abiotic	78	Salinity surface layer August average Gulf of Finland	unitless dimension	hourly	Gulf of Finland	original data; Maljutenko and Raudsepp, 2014
Sea abiotic	79	Salinity bottom layer August average Gulf of Finland	unitless dimension	hourly	Gulf of Finland	original data; Maljutenko and Raudsepp, 2014
Sea abiotic	80	Salinity surface layer August average Gulf of Riga	unitless dimension	hourly	Gulf of Riga	original data; Maljutenko and Raudsepp, 2014
Sea abiotic	81	Salinity bottom layer August average Gulf of Riga	unitless dimension	hourly	Gulf of Riga	original data; Maljutenko and Raudsepp, 2014
Sea abiotic	82	Salinity surface layer August average Baltic Proper	unitless dimension	hourly	Baltic Proper	original data; Maljutenko and Raudsepp, 2014
Sea abiotic	83	Salinity bottom layer August average Baltic Proper	unitless dimension	hourly	Baltic Proper	original data; Maljutenko and Raudsepp, 2014
Sea abiotic	84	September average Gulf of Finland	unitless dimension	hourly	Gulf of Finland	original data; Maljutenko and Raudsepp, 2014
Sea abiotic	85	September average Gulf of Finland	unitless dimension	hourly	Gulf of Finland	original data; Maljutenko and Raudsepp, 2014
Sea abiotic	86	September average Gulf of Riga	unitless dimension	hourly	Gulf of Riga	original data; Maljutenko and Raudsepp, 2014

Sea abiotic	87	September average Gulf of Riga	unitless dimension	hourly	Gulf of Riga	original data; Maljutenko and Raudsepp, 2014
Sea abiotic	88	September average Baltic Proper	unitless dimension	hourly	Baltic Proper	original data; Maljutenko and Raudsepp, 2014
Sea abiotic	89	September average Baltic Proper	unitless dimension	hourly	Baltic Proper	original data; Maljutenko and Raudsepp, 2014
Sea abiotic	90	Salinity surface layer October average Gulf of Finland	unitless dimension	hourly	Gulf of Finland	original data; Maljutenko and Raudsepp, 2014
Sea abiotic	91	Salinity bottom layer October average Gulf of Finland	unitless dimension	hourly	Gulf of Finland	original data; Maljutenko and Raudsepp, 2014
Sea abiotic	92	Salinity surface layer October average Gulf of Riga	unitless dimension	hourly	Gulf of Riga	original data; Maljutenko and Raudsepp, 2014
Sea abiotic	93	Salinity bottom layer October average Gulf of Riga	unitless dimension	hourly	Gulf of Riga	original data; Maljutenko and Raudsepp, 2014
Sea abiotic	94	Salinity surface layer October average Baltic Proper	unitless dimension	hourly	Baltic Proper	original data; Maljutenko and Raudsepp, 2014
Sea abiotic	95	Salinity bottom layer October average Baltic Proper	unitless dimension	hourly	Baltic Proper	original data; Maljutenko and Raudsepp, 2014
Sea abiotic	96	November average Gulf of Finland	unitless dimension	hourly	Gulf of Finland	original data; Maljutenko and Raudsepp, 2014
Sea abiotic	97	November average Gulf of Finland	unitless dimension	hourly	Gulf of Finland	original data; Maljutenko and Raudsepp, 2014
Sea abiotic	98	November average Gulf of Riga	unitless dimension	hourly	Gulf of Riga	original data; Maljutenko and Raudsepp, 2014
Sea abiotic	99	November average Gulf of Riga	unitless dimension	hourly	Gulf of Riga	original data; Maljutenko and Raudsepp, 2014
Sea abiotic	100	November average Baltic Proper	unitless dimension	hourly	Baltic Proper	original data; Maljutenko and Raudsepp, 2014
Sea abiotic	101	November average Baltic Proper	unitless dimension	hourly	Baltic Proper	original data; Maljutenko and Raudsepp, 2014

Sea abiotic	102	December average Gulf of Finland	unitless dimension	hourly	Gulf of Finland	original data; Maljutenko and Raudsepp, 2014
Sea abiotic	103	December average Gulf of Finland	unitless dimension	hourly	Gulf of Finland	original data; Maljutenko and Raudsepp, 2014
Sea abiotic	104	December average Gulf of Riga	unitless dimension	hourly	Gulf of Riga	original data; Maljutenko and Raudsepp, 2014
Sea abiotic	105	December average Gulf of Riga	unitless dimension	hourly	Gulf of Riga	original data; Maljutenko and Raudsepp, 2014
Sea abiotic	106	December average Baltic Proper	unitless dimension	hourly	Baltic Proper	original data; Maljutenko and Raudsepp, 2014
Sea abiotic	107	December average Baltic Proper	unitless dimension	hourly	Baltic Proper	original data; Maljutenko and Raudsepp, 2014
Sea abiotic	108	Salinity surface layer winter average Gulf of Finland	unitless dimension	hourly	Gulf of Finland	original data; Maljutenko and Raudsepp, 2014
Sea abiotic	109	Salinity bottom layer winter average Gulf of Finland	unitless dimension	hourly	Gulf of Finland	original data; Maljutenko and Raudsepp, 2014
Sea abiotic	110	Salinity surface layer winter average Gulf of Riga	unitless dimension	hourly	Gulf of Riga	original data; Maljutenko and Raudsepp, 2014
Sea abiotic	111	Salinity bottom layer winter average Gulf of Riga	unitless dimension	hourly	Gulf of Riga	original data; Maljutenko and Raudsepp, 2014
Sea abiotic	112	Salinity surface layer winter average Baltic Proper	unitless dimension	hourly	Baltic Proper	original data; Maljutenko and Raudsepp, 2014
Sea abiotic	113	Salinity bottom layer winter average Baltic Proper	unitless dimension	hourly	Baltic Proper	original data; Maljutenko and Raudsepp, 2014
Sea abiotic	114	Salinity surface layer spring average Gulf of Finland	unitless dimension	hourly	Gulf of Finland	original data; Maljutenko and Raudsepp, 2014
Sea abiotic	115	Salinity bottom layer spring average Gulf of Finland	unitless dimension	hourly	Gulf of Finland	original data; Maljutenko and Raudsepp, 2014
Sea abiotic	116	Salinity surface layer spring average Gulf of Riga	unitless dimension	hourly	Gulf of Riga	original data; Maljutenko and Raudsepp, 2014

Sea abiotic	117	Salinity bottom layer spring average Gulf of Riga	unitless dimension	hourly	Gulf of Riga	original data; Maljutenko and Raudsepp, 2014
Sea abiotic	118	Salinity surface layer spring average Baltic Proper	unitless dimension	hourly	Baltic Proper	original data; Maljutenko and Raudsepp, 2014
Sea abiotic	119	Salinity bottom layer spring average Baltic Proper	unitless dimension	hourly	Baltic Proper	original data; Maljutenko and Raudsepp, 2014
Sea abiotic	120	Salinity surface layer summer average Gulf of Finland	unitless dimension	hourly	Gulf of Finland	original data; Maljutenko and Raudsepp, 2014
Sea abiotic	121	Salinity bottom layer summer average Gulf of Finland	unitless dimension	hourly	Gulf of Finland	original data; Maljutenko and Raudsepp, 2014
Sea abiotic	122	Salinity surface layer summer average Gulf of Riga	unitless dimension	hourly	Gulf of Riga	original data; Maljutenko and Raudsepp, 2014
Sea abiotic	123	Salinity bottom layer summer average Gulf of Riga	unitless dimension	hourly	Gulf of Riga	original data; Maljutenko and Raudsepp, 2014
Sea abiotic	124	Salinity surface layer summer average Baltic Proper	unitless dimension	hourly	Baltic Proper	original data; Maljutenko and Raudsepp, 2014
Sea abiotic	125	Salinity bottom layer summer average Baltic Proper	unitless dimension	hourly	Baltic Proper	original data; Maljutenko and Raudsepp, 2014
Sea abiotic	126	Salinity surface layer autumn average Gulf of Finland	unitless dimension	hourly	Gulf of Finland	original data; Maljutenko and Raudsepp, 2014
Sea abiotic	127	Salinity bottom layer autumn average Gulf of Finland	unitless dimension	hourly	Gulf of Finland	original data; Maljutenko and Raudsepp, 2014
Sea abiotic	128	Salinity surface layer autumn average Gulf of Riga	unitless dimension	hourly	Gulf of Riga	original data; Maljutenko and Raudsepp, 2014
Sea abiotic	129	Salinity bottom layer autumn average Gulf of Riga	unitless dimension	hourly	Gulf of Riga	original data; Maljutenko and Raudsepp, 2014
Sea abiotic	130	Salinity surface layer autumn average Baltic Proper	unitless dimension	hourly	Baltic Proper	original data; Maljutenko and Raudsepp, 2014
Sea abiotic	131	Salinity bottom layer autumn average Baltic Proper	unitless dimension	hourly	Baltic Proper	original data; Maljutenko and Raudsepp, 2014

Sea abiotic	132	Salinity surface layer yearly average Gulf of Finland	unitless dimension	hourly	Gulf of Finland	original data; Maljutenko and Raudsepp, 2014
Sea abiotic	133	Salinity bottom layer yearly average Gulf of Finland	unitless dimension	hourly	Gulf of Finland	original data; Maljutenko and Raudsepp, 2014
Sea abiotic	134	Salinity surface layer yearly average Gulf of Riga	unitless dimension	hourly	Gulf of Riga	original data; Maljutenko and Raudsepp, 2014
Sea abiotic	135	Salinity bottom layer yearly average Gulf of Riga	unitless dimension	hourly	Gulf of Riga	original data; Maljutenko and Raudsepp, 2014
Sea abiotic	136	Salinity surface layer yearly average Baltic Proper	unitless dimension	hourly	Baltic Proper	original data; Maljutenko and Raudsepp, 2014
Sea abiotic	137	Salinity bottom layer yearly average Baltic Proper	unitless dimension	hourly	Baltic Proper	original data; Maljutenko and Raudsepp, 2014
Sea abiotic	138	Water temperature surface layer January average Gulf of Finland	°C	hourly	Gulf of Finland	original data; Maljutenko and Raudsepp, 2014
Sea abiotic	139	Water temperature bottom layer January average Gulf of Finland	°C	hourly	Gulf of Finland	original data; Maljutenko and Raudsepp, 2014
Sea abiotic	140	Water temperature surface layer January average Gulf of Riga	°C	hourly	Gulf of Riga	original data; Maljutenko and Raudsepp, 2014
Sea abiotic	141	Water temperature bottom layer January average Gulf of Riga	°C	hourly	Gulf of Riga	original data; Maljutenko and Raudsepp, 2014
Sea abiotic	142	Water temperature surface layer January average Baltic Proper	°C	hourly	Baltic Proper	original data; Maljutenko and Raudsepp, 2014
Sea abiotic	143	Water temperature bottom layer January average Baltic Proper	°C	hourly	Baltic Proper	original data; Maljutenko and Raudsepp, 2014

Sea abiotic	144	Water temperature surface layer February average Gulf of Finland	°C	hourly	Gulf of Finland	original data; Maljutenko and Raudsepp, 2014
Sea abiotic	145	Water temperature bottom layer February average Gulf of Finland	°C	hourly	Gulf of Finland	original data; Maljutenko and Raudsepp, 2014
Sea abiotic	146	Water temperature surface layer February average Gulf of Riga	°C	hourly	Gulf of Riga	original data; Maljutenko and Raudsepp, 2014
Sea abiotic	147	Water temperature bottom layer February average Gulf of Riga	°C	hourly	Gulf of Riga	original data; Maljutenko and Raudsepp, 2014
Sea abiotic	148	Water temperature surface layer February average Baltic Proper	°C	hourly	Baltic Proper	original data; Maljutenko and Raudsepp, 2014
Sea abiotic	149	Water temperature bottom layer February average Baltic Proper	°C	hourly	Baltic Proper	original data; Maljutenko and Raudsepp, 2014
Sea abiotic	150	Water temperature surface layer March average Gulf of Finland	°C	hourly	Gulf of Finland	original data; Maljutenko and Raudsepp, 2014
Sea abiotic	151	Water temperature bottom layer March average Gulf of Finland	°C	hourly	Gulf of Finland	original data; Maljutenko and Raudsepp, 2014
Sea abiotic	152	Water temperature surface layer March average Gulf of Riga	°C	hourly	Gulf of Riga	original data; Maljutenko and Raudsepp, 2014
Sea abiotic	153	Water temperature bottom layer March average Gulf of Riga	°C	hourly	Gulf of Riga	original data; Maljutenko and Raudsepp, 2014

Sea abiotic	154	Water temperature surface layer March average Baltic Proper	°C	hourly	Baltic Proper	original data; Maljutenko and Raudsepp, 2014
Sea abiotic	155	Water temperature bottom layer March average Baltic Proper	°C	hourly	Baltic Proper	original data; Maljutenko and Raudsepp, 2014
Sea abiotic	156	Water temperature surface layer April average Gulf of Finland	°C	hourly	Gulf of Finland	original data; Maljutenko and Raudsepp, 2014
Sea abiotic	157	Water temperature bottom layer April average Gulf of Finland	°C	hourly	Gulf of Finland	original data; Maljutenko and Raudsepp, 2014
Sea abiotic	158	layer April average Gulf of Riga	°C	hourly	Gulf of Riga	original data; Maljutenko and Raudsepp, 2014
Sea abiotic	159	layer April average Gulf of Riga	°C	hourly	Gulf of Riga	original data; Maljutenko and Raudsepp, 2014
Sea abiotic	160	Water temperature surface layer April average Baltic Proper	°C	hourly	Baltic Proper	original data; Maljutenko and Raudsepp, 2014
Sea abiotic	161	Water temperature bottom layer April average Baltic Proper	°C	hourly	Baltic Proper	original data; Maljutenko and Raudsepp, 2014
Sea abiotic	162	Water temperature surface layer May average Gulf of Finland	°C	hourly	Gulf of Finland	original data; Maljutenko and Raudsepp, 2014
Sea abiotic	163	Water temperature bottom layer May average Gulf of Finland	°C	hourly	Gulf of Finland	original data; Maljutenko and Raudsepp, 2014
Sea abiotic	164	layer May average Gulf of Riga	°C	hourly	Gulf of Riga	original data; Maljutenko and Raudsepp, 2014

Sea abiotic	165	layer May average Gulf of Riga	°C	hourly	Gulf of Riga	original data; Maljutenko and Raudsepp, 2014
Sea abiotic	166	Water temperature surface layer May average Baltic Proper	°C	hourly	Baltic Proper	original data; Maljutenko and Raudsepp, 2014
Sea abiotic	167	Water temperature bottom layer May average Baltic Proper	°C	hourly	Baltic Proper	original data; Maljutenko and Raudsepp, 2014
Sea abiotic	168	Water temperature surface layer June average Gulf of Finland	°C	hourly	Gulf of Finland	original data; Maljutenko and Raudsepp, 2014
Sea abiotic	169	Water temperature bottom layer June average Gulf of Finland	°C	hourly	Gulf of Finland	original data; Maljutenko and Raudsepp, 2014
Sea abiotic	170	layer June average Gulf of Riga	°C	hourly	Gulf of Riga	original data; Maljutenko and Raudsepp, 2014
Sea abiotic	171	layer June average Gulf of Riga	°C	hourly	Gulf of Riga	original data; Maljutenko and Raudsepp, 2014
Sea abiotic	172	Water temperature surface layer June average Baltic Proper	°C	hourly	Baltic Proper	original data; Maljutenko and Raudsepp, 2014
Sea abiotic	173	Water temperature bottom layer June average Baltic Proper	°C	hourly	Baltic Proper	original data; Maljutenko and Raudsepp, 2014
Sea abiotic	174	Water temperature surface layer July average Gulf of Finland	°C	hourly	Gulf of Finland	original data; Maljutenko and Raudsepp, 2014
Sea abiotic	175	Water temperature bottom layer July average Gulf of Finland	°C	hourly	Gulf of Finland	original data; Maljutenko and Raudsepp, 2014

Sea abiotic	176	layer July average Gulf of Riga	°C	hourly	Gulf of Riga	original data; Maljutenko and Raudsepp, 2014
Sea abiotic	177	layer July average Gulf of Riga	°C	hourly	Gulf of Riga	original data; Maljutenko and Raudsepp, 2014
Sea abiotic	178	layer July average Baltic Proper	°C	hourly	Baltic Proper	original data; Maljutenko and Raudsepp, 2014
Sea abiotic	179	layer July average Baltic Proper	°C	hourly	Baltic Proper	original data; Maljutenko and Raudsepp, 2014
Sea abiotic	180	Water temperature surface layer August average Gulf of Finland	°C	hourly	Gulf of Finland	original data; Maljutenko and Raudsepp, 2014
Sea abiotic	181	Water temperature bottom layer August average Gulf of Finland	°C	hourly	Gulf of Finland	original data; Maljutenko and Raudsepp, 2014
Sea abiotic	182	Water temperature surface layer August average Gulf of Riga	°C	hourly	Gulf of Riga	original data; Maljutenko and Raudsepp, 2014
Sea abiotic	183	Water temperature bottom layer August average Gulf of Riga	°C	hourly	Gulf of Riga	original data; Maljutenko and Raudsepp, 2014
Sea abiotic	184	Water temperature surface layer August average Baltic Proper	°C	hourly	Baltic Proper	original data; Maljutenko and Raudsepp, 2014
Sea abiotic	185	Water temperature bottom layer August average Baltic Proper	°C	hourly	Baltic Proper	original data; Maljutenko and Raudsepp, 2014
Sea abiotic	186	Water temperature surface layer September average Gulf of Finland	°C	hourly	Gulf of Finland	original data; Maljutenko and Raudsepp, 2014

Sea abiotic	187	Water temperature bottom layer September average Gulf of Finland	°C	hourly	Gulf of Finland	original data; Maljutenko and Raudsepp, 2014
Sea abiotic	188	Water temperature surface layer September average Gulf of Riga	°C	hourly	Gulf of Riga	original data; Maljutenko and Raudsepp, 2014
Sea abiotic	189	Water temperature bottom layer September average Gulf of Riga	°C	hourly	Gulf of Riga	original data; Maljutenko and Raudsepp, 2014
Sea abiotic	190	Water temperature surface layer September average Baltic Proper	°C	hourly	Baltic Proper	original data; Maljutenko and Raudsepp, 2014
Sea abiotic	191	Water temperature bottom layer September average Baltic Proper	°C	hourly	Baltic Proper	original data; Maljutenko and Raudsepp, 2014
Sea abiotic	192	Water temperature surface layer October average Gulf of Finland	°C	hourly	Gulf of Finland	original data; Maljutenko and Raudsepp, 2014
Sea abiotic	193	Water temperature bottom layer October average Gulf of Finland	°C	hourly	Gulf of Finland	original data; Maljutenko and Raudsepp, 2014
Sea abiotic	194	Water temperature surface layer October average Gulf of Riga	°C	hourly	Gulf of Riga	original data; Maljutenko and Raudsepp, 2014
Sea abiotic	195	Water temperature bottom layer October average Gulf of Riga	°C	hourly	Gulf of Riga	original data; Maljutenko and Raudsepp, 2014
Sea abiotic	196	Water temperature surface layer October average Baltic Proper	°C	hourly	Baltic Proper	original data; Maljutenko and Raudsepp, 2014

Sea abiotic	197	Water temperature bottom layer October average Baltic Proper	°C	hourly	Baltic Proper	original data; Maljutenko and Raudsepp, 2014
Sea abiotic	198	Water temperature surface layer November average Gulf of Finland	°C	hourly	Gulf of Finland	original data; Maljutenko and Raudsepp, 2014
Sea abiotic	199	Water temperature bottom layer November average Gulf of Finland	°C	hourly	Gulf of Finland	original data; Maljutenko and Raudsepp, 2014
Sea abiotic	200	Water temperature surface layer November average Gulf of Riga	°C	hourly	Gulf of Riga	original data; Maljutenko and Raudsepp, 2014
Sea abiotic	201	Water temperature bottom layer November average Gulf of Riga	°C	hourly	Gulf of Riga	original data; Maljutenko and Raudsepp, 2014
Sea abiotic	202	Water temperature surface layer November average Baltic Proper	°C	hourly	Baltic Proper	original data; Maljutenko and Raudsepp, 2014
Sea abiotic	203	Water temperature bottom layer November average Baltic Proper	°C	hourly	Baltic Proper	original data; Maljutenko and Raudsepp, 2014
Sea abiotic	204	Water temperature surface layer December average Gulf of Finland	°C	hourly	Gulf of Finland	original data; Maljutenko and Raudsepp, 2014
Sea abiotic	205	Water temperature bottom layer December average Gulf of Finland	°C	hourly	Gulf of Finland	original data; Maljutenko and Raudsepp, 2014
Sea abiotic	206	Water temperature surface layer December average Gulf of Riga	°C	hourly	Gulf of Riga	original data; Maljutenko and Raudsepp, 2014

Sea abiotic	207	Water temperature bottom layer December average Gulf of Riga	°C	hourly	Gulf of Riga	original data; Maljutenko and Raudsepp, 2014
Sea abiotic	208	Water temperature surface layer December average Baltic Proper	°C	hourly	Baltic Proper	original data; Maljutenko and Raudsepp, 2014
Sea abiotic	209	Water temperature bottom layer December average Baltic Proper	°C	hourly	Baltic Proper	original data; Maljutenko and Raudsepp, 2014
Sea abiotic	210	Water temperature surface layer winter average Gulf of Finland	°C	hourly	Gulf of Finland	original data; Maljutenko and Raudsepp, 2014
Sea abiotic	211	Water temperature bottom layer winter average Gulf of Finland	°C	hourly	Gulf of Finland	original data; Maljutenko and Raudsepp, 2014
Sea abiotic	212	Water temperature surface layer winter average Gulf of Riga	°C	hourly	Gulf of Riga	original data; Maljutenko and Raudsepp, 2014
Sea abiotic	213	Water temperature bottom layer winter average Gulf of Riga	°C	hourly	Gulf of Riga	original data; Maljutenko and Raudsepp, 2014
Sea abiotic	214	Water temperature surface layer winter average Baltic Proper	°C	hourly	Baltic Proper	original data; Maljutenko and Raudsepp, 2014
Sea abiotic	215	Water temperature bottom layer winter average Baltic Proper	°C	hourly	Baltic Proper	original data; Maljutenko and Raudsepp, 2014
Sea abiotic	216	Water temperature surface layer spring average Gulf of Finland	°C	hourly	Gulf of Finland	original data; Maljutenko and Raudsepp, 2014

Sea abiotic	217	Water temperature bottom layer spring average Gulf of Finland	°C	hourly	Gulf of Finland	original data; Maljutenko and Raudsepp, 2014
Sea abiotic	218	Water temperature surface layer spring average Gulf of Riga	°C	hourly	Gulf of Riga	original data; Maljutenko and Raudsepp, 2014
Sea abiotic	219	Water temperature bottom layer spring average Gulf of Riga	°C	hourly	Gulf of Riga	original data; Maljutenko and Raudsepp, 2014
Sea abiotic	220	Water temperature surface layer spring average Baltic Proper	°C	hourly	Baltic Proper	original data; Maljutenko and Raudsepp, 2014
Sea abiotic	221	Water temperature bottom layer spring average Baltic Proper	°C	hourly	Baltic Proper	original data; Maljutenko and Raudsepp, 2014
Sea abiotic	222	Water temperature surface layer summer average Gulf of Finland	°C	hourly	Gulf of Finland	original data; Maljutenko and Raudsepp, 2014
Sea abiotic	223	Water temperature bottom layer summer average Gulf of Finland	°C	hourly	Gulf of Finland	original data; Maljutenko and Raudsepp, 2014
Sea abiotic	224	Water temperature surface layer summer average Gulf of Riga	°C	hourly	Gulf of Riga	original data; Maljutenko and Raudsepp, 2014
Sea abiotic	225	Water temperature bottom layer summer average Gulf of Riga	°C	hourly	Gulf of Riga	original data; Maljutenko and Raudsepp, 2014
Sea abiotic	226	Water temperature surface layer summer average Baltic Proper	°C	hourly	Baltic Proper	original data; Maljutenko and Raudsepp, 2014

Sea abiotic	227	Water temperature bottom layer summer average Baltic Proper	°C	hourly	Baltic Proper	original data; Maljutenko and Raudsepp, 2014
Sea abiotic	228	Water temperature surface layer autumn average Gulf of Finland	°C	hourly	Gulf of Finland	original data; Maljutenko and Raudsepp, 2014
Sea abiotic	229	Water temperature bottom layer autumn average Gulf of Finland	°C	hourly	Gulf of Finland	original data; Maljutenko and Raudsepp, 2014
Sea abiotic	230	Water temperature surface layer autumn average Gulf of Riga	°C	hourly	Gulf of Riga	original data; Maljutenko and Raudsepp, 2014
Sea abiotic	231	Water temperature bottom layer autumn average Gulf of Riga	°C	hourly	Gulf of Riga	original data; Maljutenko and Raudsepp, 2014
Sea abiotic	232	Water temperature surface layer autumn average Baltic Proper	°C	hourly	Baltic Proper	original data; Maljutenko and Raudsepp, 2014
Sea abiotic	233	Water temperature bottom layer autumn average Baltic Proper	°C	hourly	Baltic Proper	original data; Maljutenko and Raudsepp, 2014
Sea abiotic	234	Water temperature surface layer yearly average Gulf of Finland	°C	hourly	Gulf of Finland	original data; Maljutenko and Raudsepp, 2014
Sea abiotic	235	Water temperature bottom layer yearly average Gulf of Finland	°C	hourly	Gulf of Finland	original data; Maljutenko and Raudsepp, 2014
Sea abiotic	236	Water temperature surface layer yearly average Gulf of Riga	°C	hourly	Gulf of Riga	original data; Maljutenko and Raudsepp, 2014

Sea abiotic	237	Water temperature bottom layer yearly average Gulf of Riga	°C	hourly	Gulf of Riga	original data; Maljutenko and Raudsepp, 2014
Sea abiotic	238	Water temperature surface layer yearly average Baltic Proper	°C	hourly	Baltic Proper	original data; Maljutenko and Raudsepp, 2014
Sea abiotic	239	Water temperature bottom layer yearly average Baltic Proper	°C	hourly	Baltic Proper	original data; Maljutenko and Raudsepp, 2014
Sea abiotic	240	January average Gulf of Finland	m per s	hourly	Gulf of Finland	original data; Maljutenko and Raudsepp, 2014
Sea abiotic	241	January average Gulf of Finland	m per s	hourly	Gulf of Finland	original data; Maljutenko and Raudsepp, 2014
Sea abiotic	242	Current velocity surface layer January average Gulf of Riga	m per s	hourly	Gulf of Riga	original data; Maljutenko and Raudsepp, 2014
Sea abiotic	243	Current velocity bottom layer January average Gulf of Riga	m per s	hourly	Gulf of Riga	original data; Maljutenko and Raudsepp, 2014
Sea abiotic	244	Current velocity surface layer January average Baltic Proper	m per s	hourly	Baltic Proper	original data; Maljutenko and Raudsepp, 2014
Sea abiotic	245	Current velocity bottom layer January average Baltic Proper	m per s	hourly	Baltic Proper	original data; Maljutenko and Raudsepp, 2014
Sea abiotic	246	Current velocity surface layer February average Gulf of Finland	m per s	hourly	Gulf of Finland	original data; Maljutenko and Raudsepp, 2014
Sea abiotic	247	Current velocity bottom layer February average Gulf of Finland	m per s	hourly	Gulf of Finland	original data; Maljutenko and Raudsepp, 2014
Sea abiotic	248	Current velocity surface layer February average Gulf of Riga	m per s	hourly	Gulf of Riga	original data; Maljutenko and Raudsepp, 2014
Sea abiotic	249	Current velocity bottom layer February average Gulf of Riga	m per s	hourly	Gulf of Riga	original data; Maljutenko and Raudsepp, 2014

Sea abiotic	250	February average Baltic Proper	m per s	hourly	Baltic Proper	original data; Maljutenko and Raudsepp, 2014
Sea abiotic	251	February average Baltic Proper	m per s	hourly	Baltic Proper	original data; Maljutenko and Raudsepp, 2014
Sea abiotic	252	March average Gulf of Finland	m per s	hourly	Gulf of Finland	original data; Maljutenko and Raudsepp, 2014
Sea abiotic	253	March average Gulf of Finland	m per s	hourly	Gulf of Finland	original data; Maljutenko and Raudsepp, 2014
Sea abiotic	254	Current velocity surface layer March average Gulf of Riga	m per s	hourly	Gulf of Riga	original data; Maljutenko and Raudsepp, 2014
Sea abiotic	255	Current velocity bottom layer March average Gulf of Riga	m per s	hourly	Gulf of Riga	original data; Maljutenko and Raudsepp, 2014
Sea abiotic	256	Current velocity surface layer March average Baltic Proper	m per s	hourly	Baltic Proper	original data; Maljutenko and Raudsepp, 2014
Sea abiotic	257	Current velocity bottom layer March average Baltic Proper	m per s	hourly	Baltic Proper	original data; Maljutenko and Raudsepp, 2014
Sea abiotic	258	Current velocity surface layer April average Gulf of Finland	m per s	hourly	Gulf of Finland	original data; Maljutenko and Raudsepp, 2014
Sea abiotic	259	Current velocity bottom layer April average Gulf of Finland	m per s	hourly	Gulf of Finland	original data; Maljutenko and Raudsepp, 2014
Sea abiotic	260	Current velocity surface layer April average Gulf of Riga	m per s	hourly	Gulf of Riga	original data; Maljutenko and Raudsepp, 2014
Sea abiotic	261	Current velocity bottom layer April average Gulf of Riga	m per s	hourly	Gulf of Riga	original data; Maljutenko and Raudsepp, 2014
Sea abiotic	262	Current velocity surface layer April average Baltic Proper	m per s	hourly	Baltic Proper	original data; Maljutenko and Raudsepp, 2014
Sea abiotic	263	Current velocity bottom layer April average Baltic Proper	m per s	hourly	Baltic Proper	original data; Maljutenko and Raudsepp, 2014
Sea abiotic	264	Current velocity surface layer May average Gulf of Finland	m per s	hourly	Gulf of Finland	original data; Maljutenko and Raudsepp, 2014

Sea abiotic	265	Current velocity bottom layer May average Gulf of Finland	m per s	hourly	Gulf of Finland	original data; Maljutenko and Raudsepp, 2014
Sea abiotic	266	Current velocity surface layer May average Gulf of Riga	m per s	hourly	Gulf of Riga	original data; Maljutenko and Raudsepp, 2014
Sea abiotic	267	Current velocity bottom layer May average Gulf of Riga	m per s	hourly	Gulf of Riga	original data; Maljutenko and Raudsepp, 2014
Sea abiotic	268	Current velocity surface layer May average Baltic Proper	m per s	hourly	Baltic Proper	original data; Maljutenko and Raudsepp, 2014
Sea abiotic	269	Current velocity bottom layer May average Baltic Proper	m per s	hourly	Baltic Proper	original data; Maljutenko and Raudsepp, 2014
Sea abiotic	270	Current velocity surface layer June average Gulf of Finland	m per s	hourly	Gulf of Finland	original data; Maljutenko and Raudsepp, 2014
Sea abiotic	271	Current velocity bottom layer June average Gulf of Finland	m per s	hourly	Gulf of Finland	original data; Maljutenko and Raudsepp, 2014
Sea abiotic	272	Current velocity surface layer June average Gulf of Riga	m per s	hourly	Gulf of Riga	original data; Maljutenko and Raudsepp, 2014
Sea abiotic	273	Current velocity bottom layer June average Gulf of Riga	m per s	hourly	Gulf of Riga	original data; Maljutenko and Raudsepp, 2014
Sea abiotic	274	Current velocity surface layer June average Baltic Proper	m per s	hourly	Baltic Proper	original data; Maljutenko and Raudsepp, 2014
Sea abiotic	275	Current velocity bottom layer June average Baltic Proper	m per s	hourly	Baltic Proper	original data; Maljutenko and Raudsepp, 2014
Sea abiotic	276	Current velocity surface layer July average Gulf of Finland	m per s	hourly	Gulf of Finland	original data; Maljutenko and Raudsepp, 2014
Sea abiotic	277	Current velocity bottom layer July average Gulf of Finland	m per s	hourly	Gulf of Finland	original data; Maljutenko and Raudsepp, 2014
Sea abiotic	278	Current velocity surface layer July average Gulf of Riga	m per s	hourly	Gulf of Riga	original data; Maljutenko and Raudsepp, 2014
Sea abiotic	279	Current velocity bottom layer July average Gulf of Riga	m per s	hourly	Gulf of Riga	original data; Maljutenko and Raudsepp, 2014

Sea abiotic	280	Current velocity surface layer July average Baltic Proper	m per s	hourly	Baltic Proper	original data; Maljutenko and Raudsepp, 2014
Sea abiotic	281	Current velocity bottom layer July average Baltic Proper	m per s	hourly	Baltic Proper	original data; Maljutenko and Raudsepp, 2014
Sea abiotic	282	August average Gulf of Finland	m per s	hourly	Gulf of Finland	original data; Maljutenko and Raudsepp, 2014
Sea abiotic	283	August average Gulf of Finland	m per s	hourly	Gulf of Finland	original data; Maljutenko and Raudsepp, 2014
Sea abiotic	284	Current velocity surface layer August average Gulf of Riga	m per s	hourly	Gulf of Riga	original data; Maljutenko and Raudsepp, 2014
Sea abiotic	285	Current velocity bottom layer August average Gulf of Riga	m per s	hourly	Gulf of Riga	original data; Maljutenko and Raudsepp, 2014
Sea abiotic	286	Current velocity surface layer August average Baltic Proper	m per s	hourly	Baltic Proper	original data; Maljutenko and Raudsepp, 2014
Sea abiotic	287	Current velocity bottom layer August average Baltic Proper	m per s	hourly	Baltic Proper	original data; Maljutenko and Raudsepp, 2014
Sea abiotic	288	Current velocity surface layer September average Gulf of Finland	m per s	hourly	Gulf of Finland	original data; Maljutenko and Raudsepp, 2014
Sea abiotic	289	Current velocity bottom layer September average Gulf of Finland	m per s	hourly	Gulf of Finland	original data; Maljutenko and Raudsepp, 2014
Sea abiotic	290	September average Gulf of Riga	m per s	hourly	Gulf of Riga	original data; Maljutenko and Raudsepp, 2014
Sea abiotic	291	September average Gulf of Riga	m per s	hourly	Gulf of Riga	original data; Maljutenko and Raudsepp, 2014
Sea abiotic	292	Current velocity surface layer September average Baltic Proper	m per s	hourly	Baltic Proper	original data; Maljutenko and Raudsepp, 2014

Sea abiotic	293	Current velocity bottom layer September average Baltic Proper	m per s	hourly	Baltic Proper	original data; Maljutenko and Raudsepp, 2014
Sea abiotic	294	Current velocity surface layer October average Gulf of Finland	m per s	hourly	Gulf of Finland	original data; Maljutenko and Raudsepp, 2014
Sea abiotic	295	Current velocity bottom layer October average Gulf of Finland	m per s	hourly	Gulf of Finland	original data; Maljutenko and Raudsepp, 2014
Sea abiotic	296	Current velocity surface layer October average Gulf of Riga	m per s	hourly	Gulf of Riga	original data; Maljutenko and Raudsepp, 2014
Sea abiotic	297	Current velocity bottom layer October average Gulf of Riga	m per s	hourly	Gulf of Riga	original data; Maljutenko and Raudsepp, 2014
Sea abiotic	298	Current velocity surface layer October average Baltic Proper	m per s	hourly	Baltic Proper	original data; Maljutenko and Raudsepp, 2014
Sea abiotic	299	Current velocity bottom layer October average Baltic Proper	m per s	hourly	Baltic Proper	original data; Maljutenko and Raudsepp, 2014
Sea abiotic	300	Current velocity surface layer November average Gulf of Finland	m per s	hourly	Gulf of Finland	original data; Maljutenko and Raudsepp, 2014
Sea abiotic	301	Current velocity bottom layer November average Gulf of Finland	m per s	hourly	Gulf of Finland	original data; Maljutenko and Raudsepp, 2014
Sea abiotic	302	November average Gulf of Riga	m per s	hourly	Gulf of Riga	original data; Maljutenko and Raudsepp, 2014
Sea abiotic	303	November average Gulf of Riga	m per s	hourly	Gulf of Riga	original data; Maljutenko and Raudsepp, 2014
Sea abiotic	304	Current velocity surface layer November average Baltic Proper	m per s	hourly	Baltic Proper	original data; Maljutenko and Raudsepp, 2014

Sea abiotic	305	Current velocity bottom layer November average Baltic Proper	m per s	hourly	Baltic Proper	original data; Maljutenko and Raudsepp, 2014
Sea abiotic	306	Current velocity surface layer December average Gulf of Finland	m per s	hourly	Gulf of Finland	original data; Maljutenko and Raudsepp, 2014
Sea abiotic	307	Current velocity bottom layer December average Gulf of Finland	m per s	hourly	Gulf of Finland	original data; Maljutenko and Raudsepp, 2014
Sea abiotic	308	December average Gulf of Riga	m per s	hourly	Gulf of Riga	original data; Maljutenko and Raudsepp, 2014
Sea abiotic	309	December average Gulf of Riga	m per s	hourly	Gulf of Riga	original data; Maljutenko and Raudsepp, 2014
Sea abiotic	310	Current velocity surface layer December average Baltic Proper	m per s	hourly	Baltic Proper	original data; Maljutenko and Raudsepp, 2014
Sea abiotic	311	Current velocity bottom layer December average Baltic Proper	m per s	hourly	Baltic Proper	original data; Maljutenko and Raudsepp, 2014
Sea abiotic	312	winter average Gulf of Finland	m per s	hourly	Gulf of Finland	original data; Maljutenko and Raudsepp, 2014
Sea abiotic	313	winter average Gulf of Finland	m per s	hourly	Gulf of Finland	original data; Maljutenko and Raudsepp, 2014
Sea abiotic	314	Current velocity surface layer winter average Gulf of Riga	m per s	hourly	Gulf of Riga	original data; Maljutenko and Raudsepp, 2014
Sea abiotic	315	Current velocity bottom layer winter average Gulf of Riga	m per s	hourly	Gulf of Riga	original data; Maljutenko and Raudsepp, 2014
Sea abiotic	316	Current velocity surface layer winter average Baltic Proper	m per s	hourly	Baltic Proper	original data; Maljutenko and Raudsepp, 2014
Sea abiotic	317	Current velocity bottom layer winter average Baltic Proper	m per s	hourly	Baltic Proper	original data; Maljutenko and Raudsepp, 2014

Sea abiotic	318	Current velocity surface layer spring average Gulf of Finland	m per s	hourly	Gulf of Finland	original data; Maljutenko and Raudsepp, 2014
Sea abiotic	319	Current velocity bottom layer spring average Gulf of Finland	m per s	hourly	Gulf of Finland	original data; Maljutenko and Raudsepp, 2014
Sea abiotic	320	Current velocity surface layer spring average Gulf of Riga	m per s	hourly	Gulf of Riga	original data; Maljutenko and Raudsepp, 2014
Sea abiotic	321	Current velocity bottom layer spring average Gulf of Riga	m per s	hourly	Gulf of Riga	original data; Maljutenko and Raudsepp, 2014
Sea abiotic	322	Current velocity surface layer spring average Baltic Proper	m per s	hourly	Baltic Proper	original data; Maljutenko and Raudsepp, 2014
Sea abiotic	323	Current velocity bottom layer spring average Baltic Proper	m per s	hourly	Baltic Proper	original data; Maljutenko and Raudsepp, 2014
Sea abiotic	324	summer average Gulf of Finland	m per s	hourly	Gulf of Finland	original data; Maljutenko and Raudsepp, 2014
Sea abiotic	325	summer average Gulf of Finland	m per s	hourly	Gulf of Finland	original data; Maljutenko and Raudsepp, 2014
Sea abiotic	326	Current velocity surface layer summer average Gulf of Riga	m per s	hourly	Gulf of Riga	original data; Maljutenko and Raudsepp, 2014
Sea abiotic	327	Current velocity bottom layer summer average Gulf of Riga	m per s	hourly	Gulf of Riga	original data; Maljutenko and Raudsepp, 2014
Sea abiotic	328	Current velocity surface layer summer average Baltic Proper	m per s	hourly	Baltic Proper	original data; Maljutenko and Raudsepp, 2014
Sea abiotic	329	Current velocity bottom layer summer average Baltic Proper	m per s	hourly	Baltic Proper	original data; Maljutenko and Raudsepp, 2014
Sea abiotic	330	autumn average Gulf of Finland	m per s	hourly	Gulf of Finland	original data; Maljutenko and Raudsepp, 2014
Sea abiotic	331	autumn average Gulf of Finland	m per s	hourly	Gulf of Finland	original data; Maljutenko and Raudsepp, 2014
Sea abiotic	332	Current velocity surface layer autumn average Gulf of Riga	m per s	hourly	Gulf of Riga	original data; Maljutenko and Raudsepp, 2014

Sea abiotic	333	Current velocity bottom layer autumn average Gulf of Riga	m per s	hourly	Gulf of Riga	original data; Maljutenko and Raudsepp, 2014
Sea abiotic	334	Current velocity surface layer autumn average Baltic Proper	m per s	hourly	Baltic Proper	original data; Maljutenko and Raudsepp, 2014
Sea abiotic	335	Current velocity bottom layer autumn average Baltic Proper	m per s	hourly	Baltic Proper	original data; Maljutenko and Raudsepp, 2014
Sea abiotic	336	Current velocity surface layer yearly average Gulf of Finland	m per s	hourly	Gulf of Finland	original data; Maljutenko and Raudsepp, 2014
Sea abiotic	337	Current velocity bottom layer yearly average Gulf of Finland	m per s	hourly	Gulf of Finland	original data; Maljutenko and Raudsepp, 2014
Sea abiotic	338	Current velocity surface layer yearly average Gulf of Riga	m per s	hourly	Gulf of Riga	original data; Maljutenko and Raudsepp, 2014
Sea abiotic	339	Current velocity bottom layer yearly average Gulf of Riga	m per s	hourly	Gulf of Riga	original data; Maljutenko and Raudsepp, 2014
Sea abiotic	340	Current velocity surface layer yearly average Baltic Proper	m per s	hourly	Baltic Proper	original data; Maljutenko and Raudsepp, 2014
Sea abiotic	341	Current velocity bottom layer yearly average Baltic Proper	m per s	hourly	Baltic Proper	original data; Maljutenko and Raudsepp, 2014
Sea abiotic	342	Wave height yearly average Harilaid	m	hourly	station	Suursaar et al. 2015
Sea abiotic	343	Wave height yearly average Sundgrund	m	hourly	station	Suursaar et al. 2015
Sea abiotic	344	Wave height yearly average Letipea	m	hourly	station	Suursaar et al. 2015
Sea abiotic	345	Wave height yearly average Kihnu	m	hourly	station	Suursaar et al. 2015
Sea biotic	1	Herring larvae abundance yearly average	Ind. per 10 min haul	weekly	Pärnu Bay	Arula et al. 2014
Sea biotic	2	Herring larvae first annual appearance	week number	weekly	Pärnu Bay	Arula et al. 2014

Sea biotic	3	Herring larvae maximum abundance annual timing	week number	weekly	Pärnu Bay	Arula et al. 2014
Sea biotic	4	Difference in timing between appearance and maximum abundance of herring larvae yearly average	week number	weekly	Pärnu Bay	Arula et al. 2014
Sea biotic	5	Abundance of herring 1-year-old individuals yearly average	Ind. per 10 min haul	weekly	Gulf of Riga	Arula et al. 2014
Sea biotic	6	Herring spawning stock biomass yearly average	tonnes	yearly	Gulf of Riga	Ojaveer at al. 2004, ICES 2015
Sea biotic	7	Goby larvae abundance yearly average	ind. per m^3	weekly	Pärnu Bay	Laur et al. 2014
Sea biotic	8	Goby larvae first annual appearance	week number	weekly	Pärnu Bay	Laur et al. 2014
Sea biotic	9	Copepod nauplii abundance yearly average	ind. per m^3	weekly	Pärnu Bay	Arula et al. 2014
Sea biotic	10	Eurytemora nauplii abundance maximum annual timing	week number	weekly	Pärnu Bay	Arula et al. 2014
Sea biotic	11	Eurytemora affinis adults abundance yearly average	ind. per m^3	weekly	Pärnu Bay	Arula et al. 2014
Sea biotic	12	Eurytemora affinis adults abundance maximum annual timing	week number	weekly	Pärnu Bay	original data
Sea biotic	13	Acartia I-V abundance yearly average	ind. per m^3	weekly	Pärnu Bay	Laur et al. 2014
Sea biotic	14	Acartia I-V abundance maximum annual timing	week number	weekly	Pärnu Bay	Arula et al. 2014
Sea biotic	15	Acartia adult abundance yearly average	ind. per m^3	weekly	Pärnu Bay	Arula et al. 2014

Sea biotic	16	Acartia adult abundance maximum annual timing	week number	weekly	Pärnu Bay	Arula et al. 2014
Sea biotic	17	Bosmina abundance yearly average	ind. per m^3	weekly	Pärnu Bay	Laur et al. 2014
Sea biotic	18	Bosmina abundance maximum annual timing	week number	weekly	Pärnu Bay	Laur et al. 2014

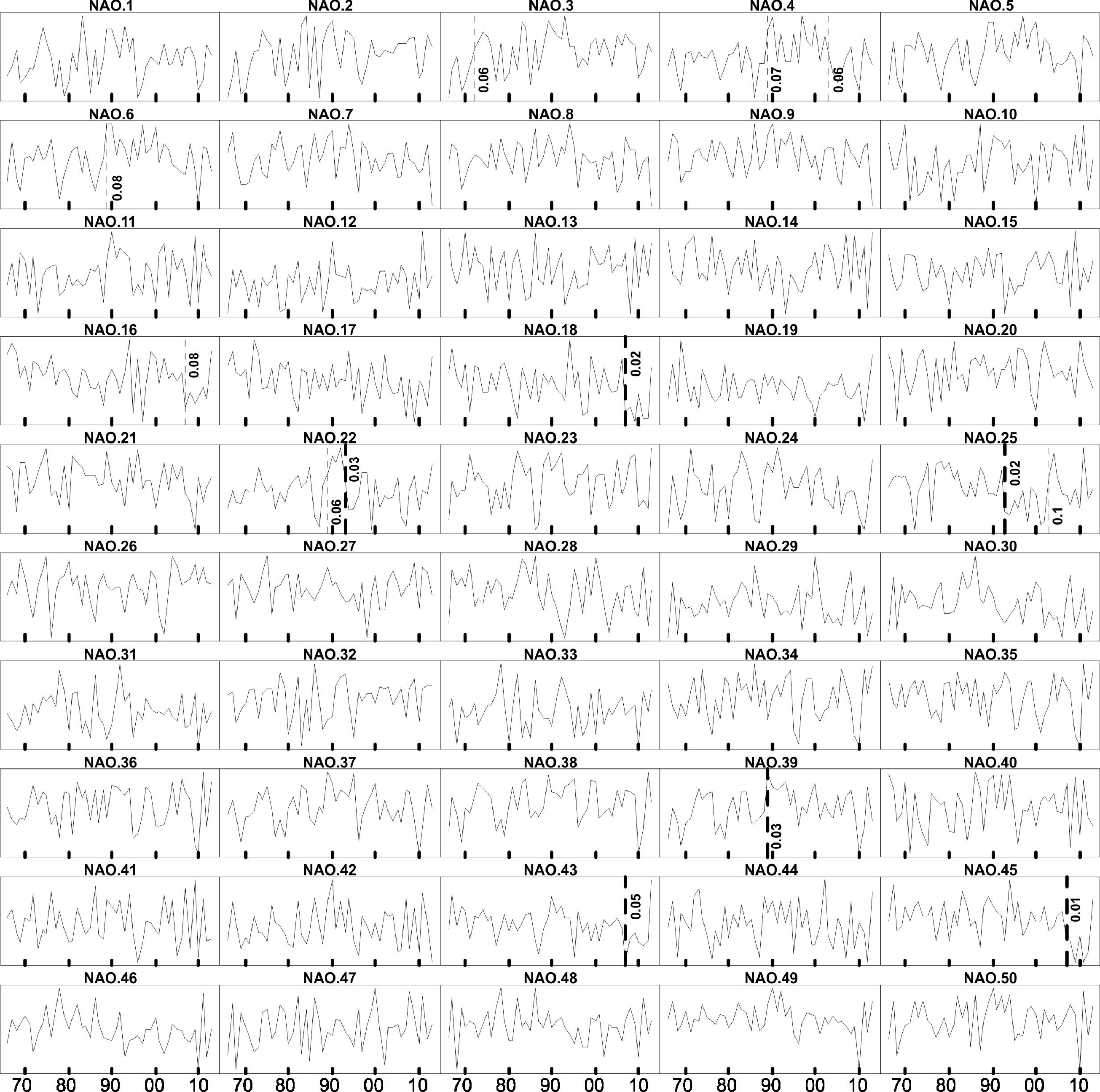
NAO.1
NAO.2
NAO.3
0.06
NAO.4
0.07
0.06
NAO.5
NAO.6
0.08
NAO.7
NAO.8
NAO.9
NAO.10
NAO.11
NAO.12
NAO.13
NAO.14
NAO.15
NAO.16
0.08
NAO.17
NAO.18
0.02
NAO.19
NAO.20
NAO.21
NAO.22
0.06
0.03
NAO.23
NAO.24
NAO.25
0.02
0.1
NAO.26
NAO.27
NAO.28
NAO.29
NAO.30
NAO.31
NAO.32
NAO.33
NAO.34
NAO.35
NAO.36
NAO.37
NAO.38
NAO.39
0.03
NAO.40
NAO.41
NAO.42
NAO.43
0.05
NAO.44
NAO.45
0.01
NAO.46
NAO.47
NAO.48
NAO.49
NAO.50
70 80 90 00 10
70 80 90 00 10
70 80 90 00 10
70 80 90 00 10
70 80 90 00 10

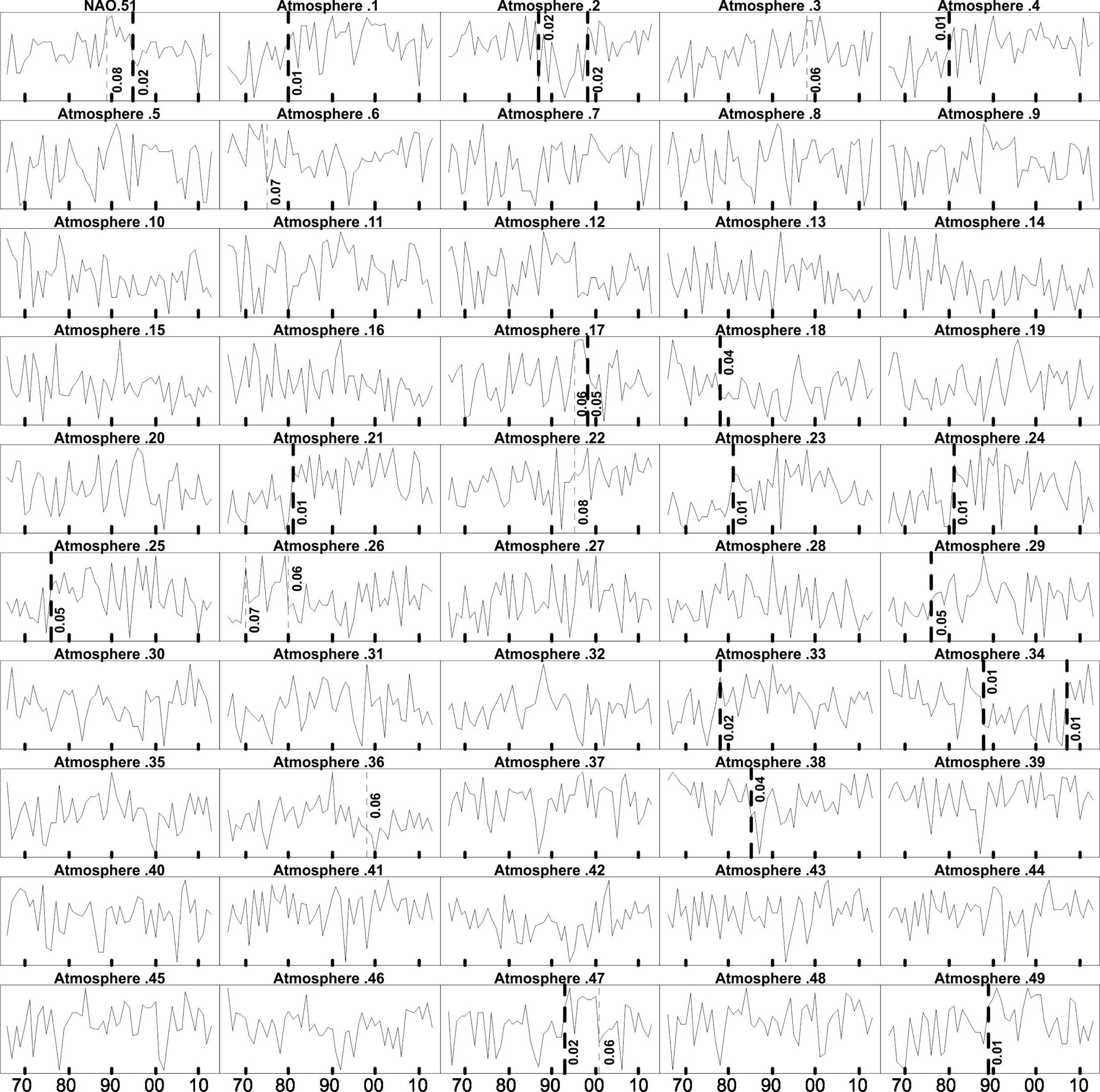
NAO.51
0.08
0.02
Atmosphere .1
0.01
Atmosphere .2
0.02
0.02
Atmosphere .3
0.06
Atmosphere .4
0.01
Atmosphere .5
Atmosphere .6
0.07
Atmosphere .7
Atmosphere .8
Atmosphere .9
Atmosphere .10
Atmosphere .11
Atmosphere .12
Atmosphere .13
Atmosphere .14
Atmosphere .15
Atmosphere .16
Atmosphere .17
0.06
0.05
Atmosphere .18
0.04
Atmosphere .19
Atmosphere .20
Atmosphere .21
0.01
Atmosphere .22
0.08
Atmosphere .23
0.01
Atmosphere .24
0.01
Atmosphere .25
0.05
Atmosphere .26
0.07
0.06
Atmosphere .27
Atmosphere .28
Atmosphere .29
0.05
Atmosphere .30
Atmosphere .31
Atmosphere .32
Atmosphere .33
0.02
Atmosphere .34
0.01
0.01
Atmosphere .35
Atmosphere .36
0.06
Atmosphere .37
Atmosphere .38
0.04
Atmosphere .39
Atmosphere .40
Atmosphere .41
Atmosphere .42
Atmosphere .43
Atmosphere .44
Atmosphere .45
Atmosphere .46
Atmosphere .47
0.02
0.06
Atmosphere .48
Atmosphere .49
0.01
70 80 90 00 10
70 80 90 00 10
70 80 90 00 10
70 80 90 00 10
70 80 90 00 10

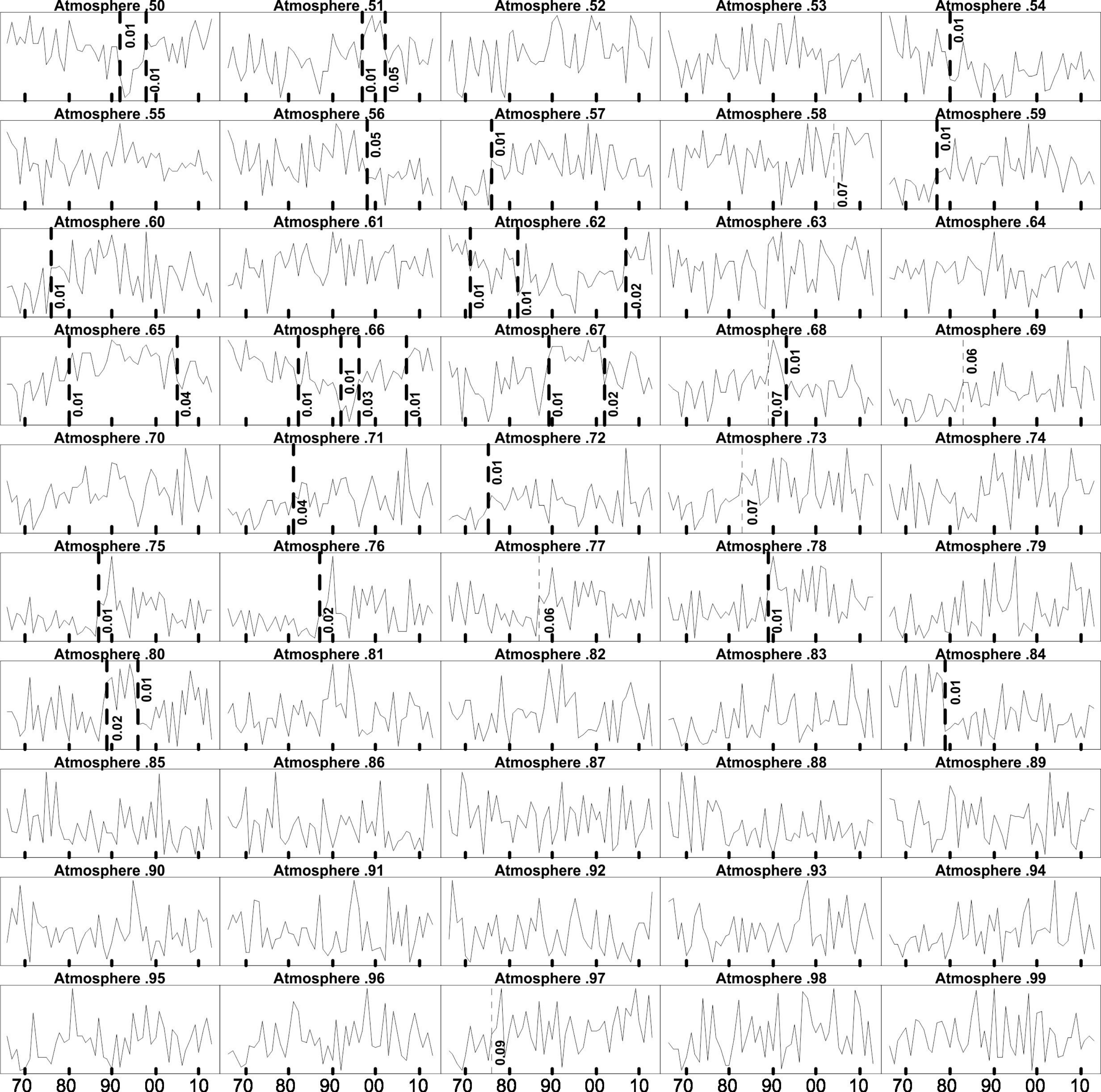
Atmosphere .50
0.01
0.01
Atmosphere .51
0.01
0.05
Atmosphere .52
Atmosphere .53
Atmosphere .54
0.01
Atmosphere .55
Atmosphere .56
0.05
Atmosphere .57
0.01
Atmosphere .58
0.07
Atmosphere .59
0.01
Atmosphere .60
0.01
Atmosphere .61
Atmosphere .62
0.01
0.01
0.02
Atmosphere .63
Atmosphere .64
Atmosphere .65
0.01
0.04
Atmosphere .66
0.01
0.01
0.03
0.01
Atmosphere .67
0.01
0.02
Atmosphere .68
0.07
0.01
Atmosphere .69
0.06
Atmosphere .70
Atmosphere .71
0.04
Atmosphere .72
0.01
Atmosphere .73
0.07
Atmosphere .74
Atmosphere .75
0.01
Atmosphere .76
0.02
Atmosphere .77
0.06
Atmosphere .78
0.01
Atmosphere .79
Atmosphere .80
0.02
0.01
Atmosphere .81
Atmosphere .82
Atmosphere .83
Atmosphere .84
0.01
Atmosphere .85
Atmosphere .86
Atmosphere .87
Atmosphere .88
Atmosphere .89
Atmosphere .90
Atmosphere .91
Atmosphere .92
Atmosphere .93
Atmosphere .94
Atmosphere .95
Atmosphere .96
Atmosphere .97
0.09
Atmosphere .98
Atmosphere .99
70 80 90 00 10
70 80 90 00 10
70 80 90 00 10
70 80 90 00 10
70 80 90 00 10

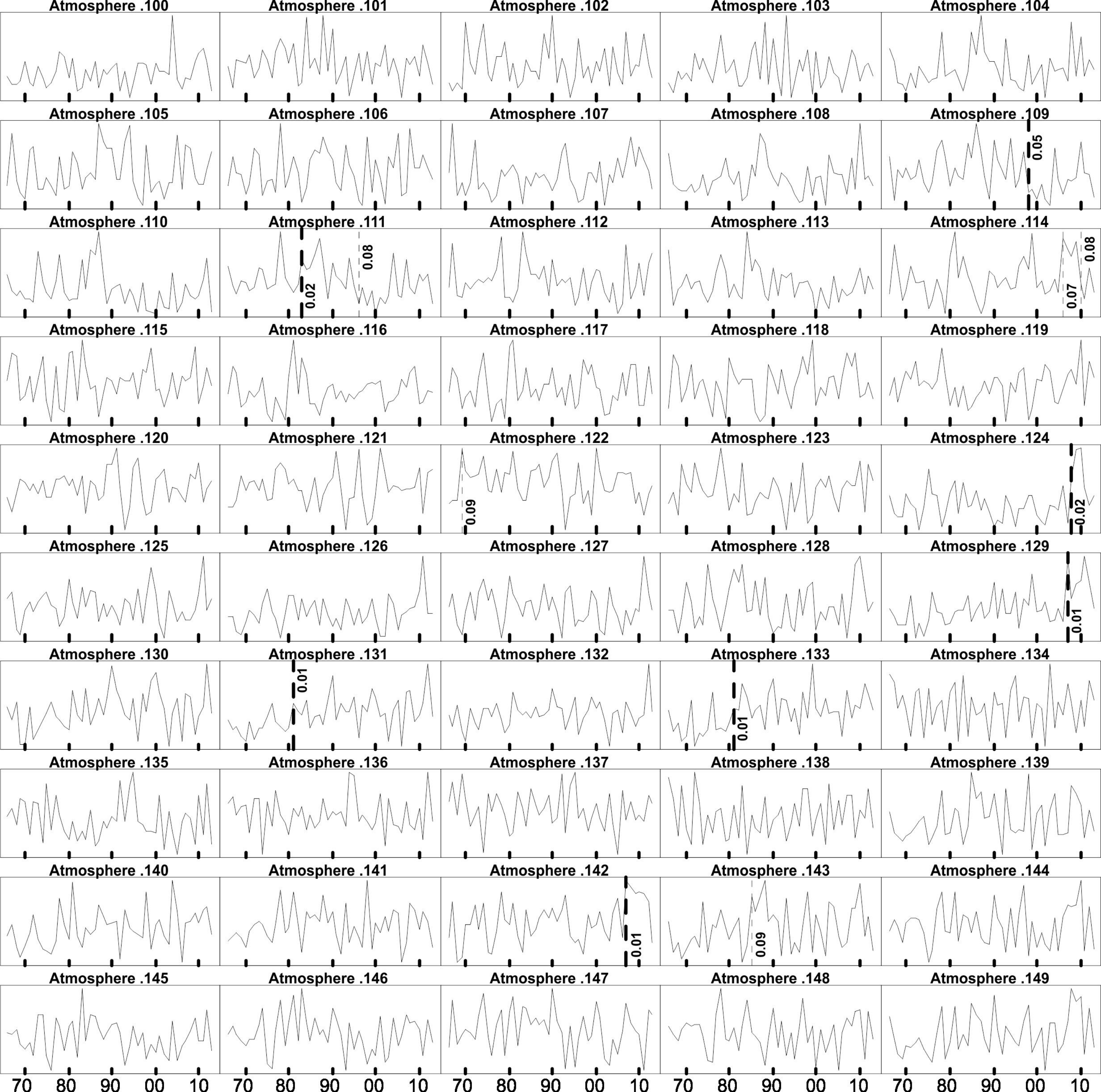
Atmosphere .100
Atmosphere .101
Atmosphere .102
Atmosphere .103
Atmosphere .104
Atmosphere .105
Atmosphere .106
Atmosphere .107
Atmosphere .108
Atmosphere .109
0.05
Atmosphere .110
Atmosphere .111
0.02
0.08
Atmosphere .112
Atmosphere .113
Atmosphere .114
0.07
0.08
Atmosphere .115
Atmosphere .116
Atmosphere .117
Atmosphere .118
Atmosphere .119
Atmosphere .120
Atmosphere .121
Atmosphere .122
0.09
Atmosphere .123
Atmosphere .124
0.02
Atmosphere .125
Atmosphere .126
Atmosphere .127
Atmosphere .128
Atmosphere .129
0.01
Atmosphere .130
Atmosphere .131
0.01
Atmosphere .132
Atmosphere .133
0.01
Atmosphere .134
Atmosphere .135
Atmosphere .136
Atmosphere .137
Atmosphere .138
Atmosphere .139
Atmosphere .140
Atmosphere .141
Atmosphere .142
0.01
Atmosphere .143
0.09
Atmosphere .144
Atmosphere .145
Atmosphere .146
Atmosphere .147
Atmosphere .148
Atmosphere .149
70 80 90 00 10

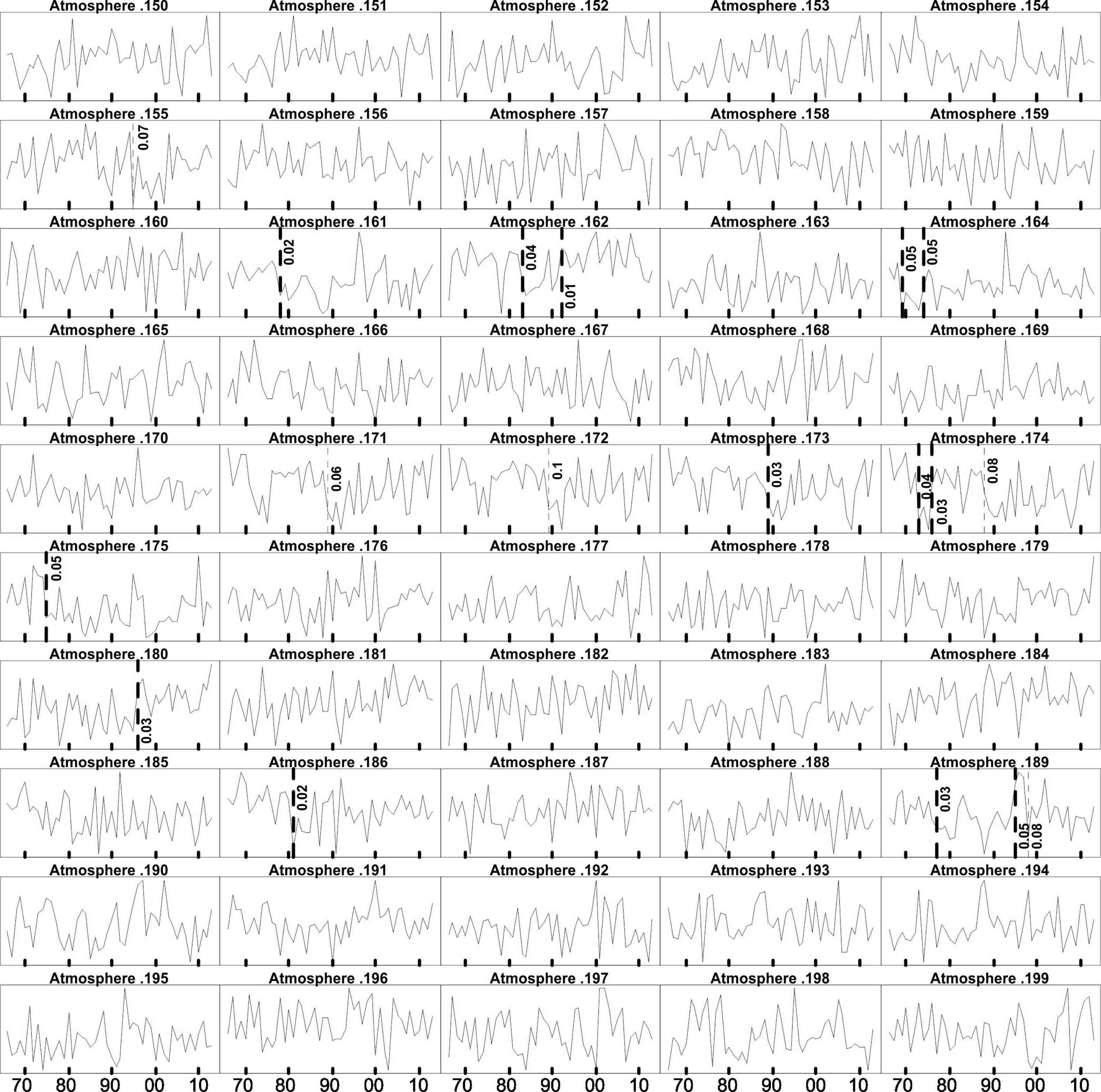
Atmosphere .150
Atmosphere .151
Atmosphere .152
Atmosphere .153
Atmosphere .154
Atmosphere .155
0.07
Atmosphere .156
Atmosphere .157
Atmosphere .158
Atmosphere .159
Atmosphere .160
Atmosphere .161
0.02
Atmosphere .162
0.04
0.01
Atmosphere .163
Atmosphere .164
0.05
0.05
Atmosphere .165
Atmosphere .166
Atmosphere .167
Atmosphere .168
Atmosphere .169
Atmosphere .170
Atmosphere .171
0.06
Atmosphere .172
0.1
Atmosphere .173
0.03
Atmosphere .174
0.04
0.03
0.08
Atmosphere .175
0.05
Atmosphere .176
Atmosphere .177
Atmosphere .178
Atmosphere .179
Atmosphere .180
0.03
Atmosphere .181
Atmosphere .182
Atmosphere .183
Atmosphere .184
Atmosphere .185
Atmosphere .186
0.02
Atmosphere .187
Atmosphere .188
Atmosphere .189
0.03
0.05
0.08
Atmosphere .190
Atmosphere .191
Atmosphere .192
Atmosphere .193
Atmosphere .194
Atmosphere .195
Atmosphere .196
Atmosphere .197
Atmosphere .198
Atmosphere .199
70 80 90 00 10
70 80 90 00 10
70 80 90 00 10
70 80 90 00 10
70 80 90 00 10

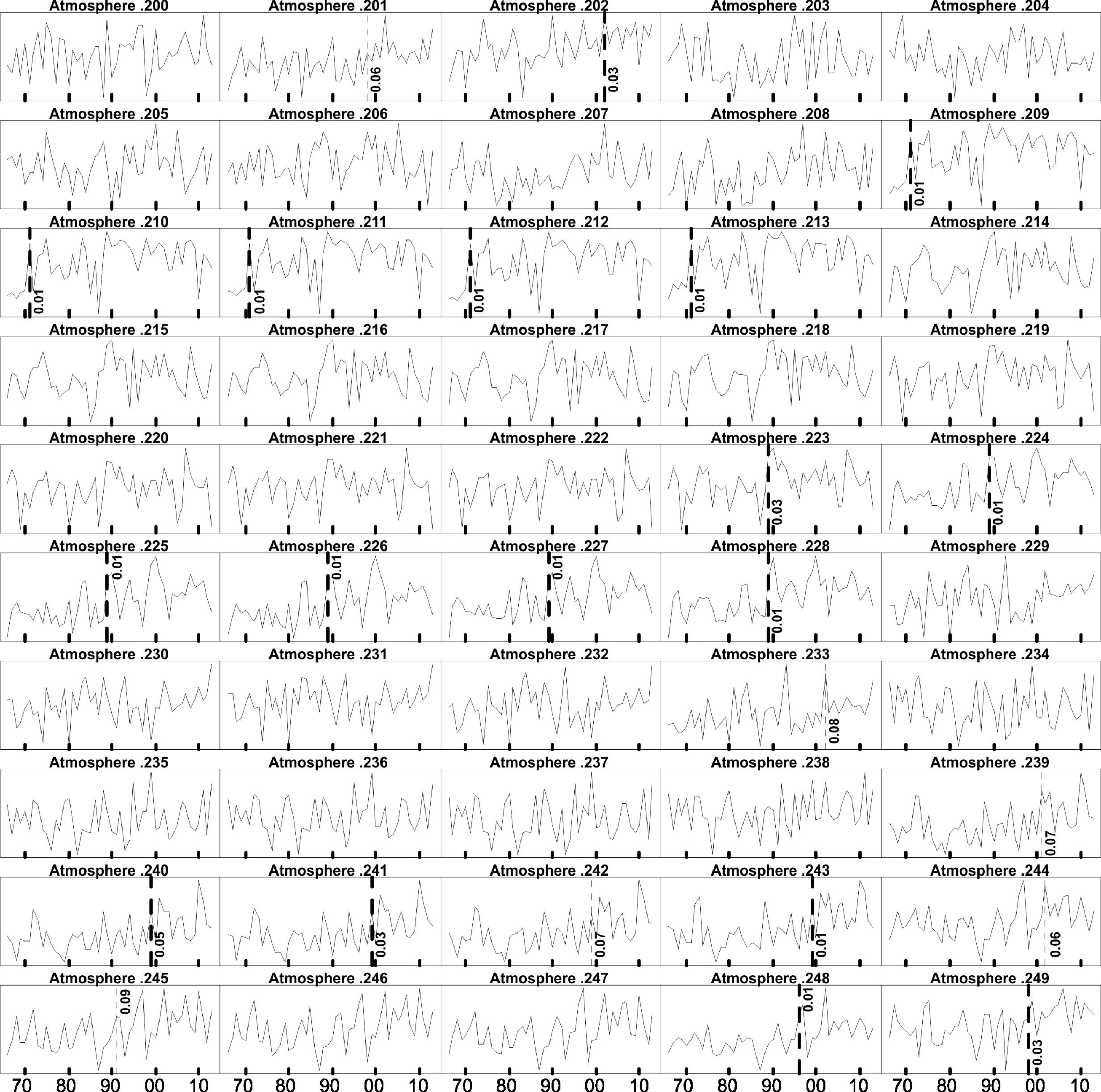
Atmosphere .200
Atmosphere .201
0.06
Atmosphere .202
0.03
Atmosphere .203
Atmosphere .204
Atmosphere .205
Atmosphere .206
Atmosphere .207
Atmosphere .208
Atmosphere .209
0.01
Atmosphere .210
0.01
Atmosphere .211
0.01
Atmosphere .212
0.01
Atmosphere .213
0.01
Atmosphere .214
Atmosphere .215
Atmosphere .216
Atmosphere .217
Atmosphere .218
Atmosphere .219
Atmosphere .220
Atmosphere .221
Atmosphere .222
Atmosphere .223
0.03
Atmosphere .224
0.01
Atmosphere .225
0.01
Atmosphere .226
0.01
Atmosphere .227
0.01
Atmosphere .228
0.01
Atmosphere .229
Atmosphere .230
Atmosphere .231
Atmosphere .232
Atmosphere .233
0.08
Atmosphere .234
Atmosphere .235
Atmosphere .236
Atmosphere .237
Atmosphere .238
Atmosphere .239
0.07
Atmosphere .240
0.05
Atmosphere .241
0.03
Atmosphere .242
0.07
Atmosphere .243
0.01
Atmosphere .244
0.06
Atmosphere .245
0.09
Atmosphere .246
Atmosphere .247
Atmosphere .248
0.01
Atmosphere .249
0.03
70 80 90 00 10
70 80 90 00 10
70 80 90 00 10
70 80 90 00 10
70 80 90 00 10

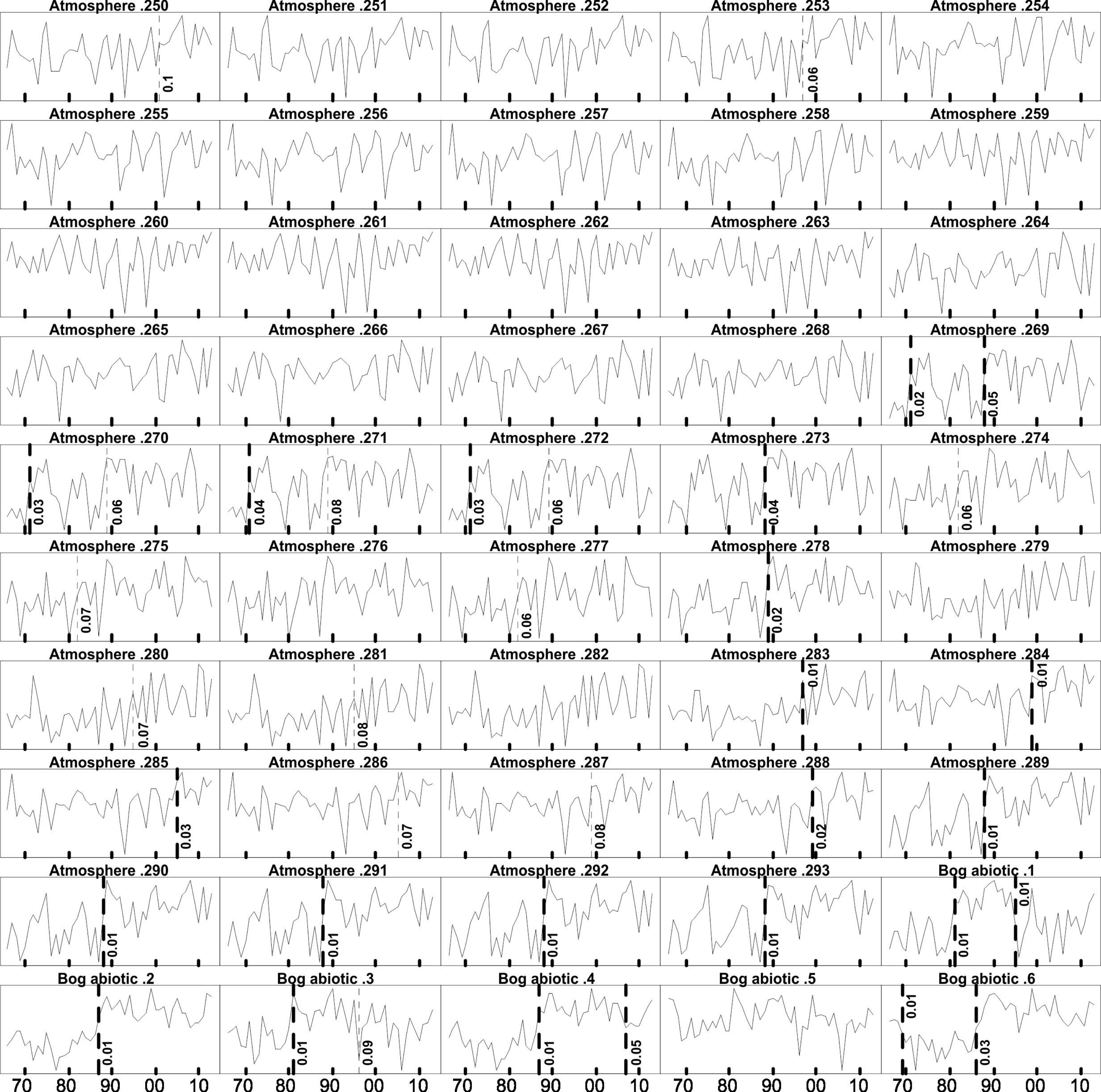

Atmosphere .250
0.1
Atmosphere .251
Atmosphere .252
Atmosphere .253
0.06
Atmosphere .254
Atmosphere .255
Atmosphere .256
Atmosphere .257
Atmosphere .258
Atmosphere .259
Atmosphere .260
Atmosphere .261
Atmosphere .262
Atmosphere .263
Atmosphere .264
Atmosphere .265
Atmosphere .266
Atmosphere .267
Atmosphere .268
Atmosphere .269
0.02
0.05
Atmosphere .270
0.03
0.06
Atmosphere .271
0.04
0.08
Atmosphere .272
0.03
0.06
Atmosphere .273
0.04
Atmosphere .274
0.06
Atmosphere .275
0.07
Atmosphere .276
Atmosphere .277
0.06
Atmosphere .278
0.02
Atmosphere .279
Atmosphere .280
0.07
Atmosphere .281
0.08
Atmosphere .282
Atmosphere .283
0.01
Atmosphere .284
0.01
Atmosphere .285
0.03
Atmosphere .286
0.07
Atmosphere .287
0.08
Atmosphere .288
0.02
Atmosphere .289
0.01
Atmosphere .290
0.01
Atmosphere .291
0.01
Atmosphere .292
0.01
Atmosphere .293
0.01
Bog abiotic .1
0.01
0.01
Bog abiotic .2
0.01
Bog abiotic .3
0.01
0.09
Bog abiotic .4
0.01
0.05
Bog abiotic .5
Bog abiotic .6
0.01
0.03
70 80 90 00 10

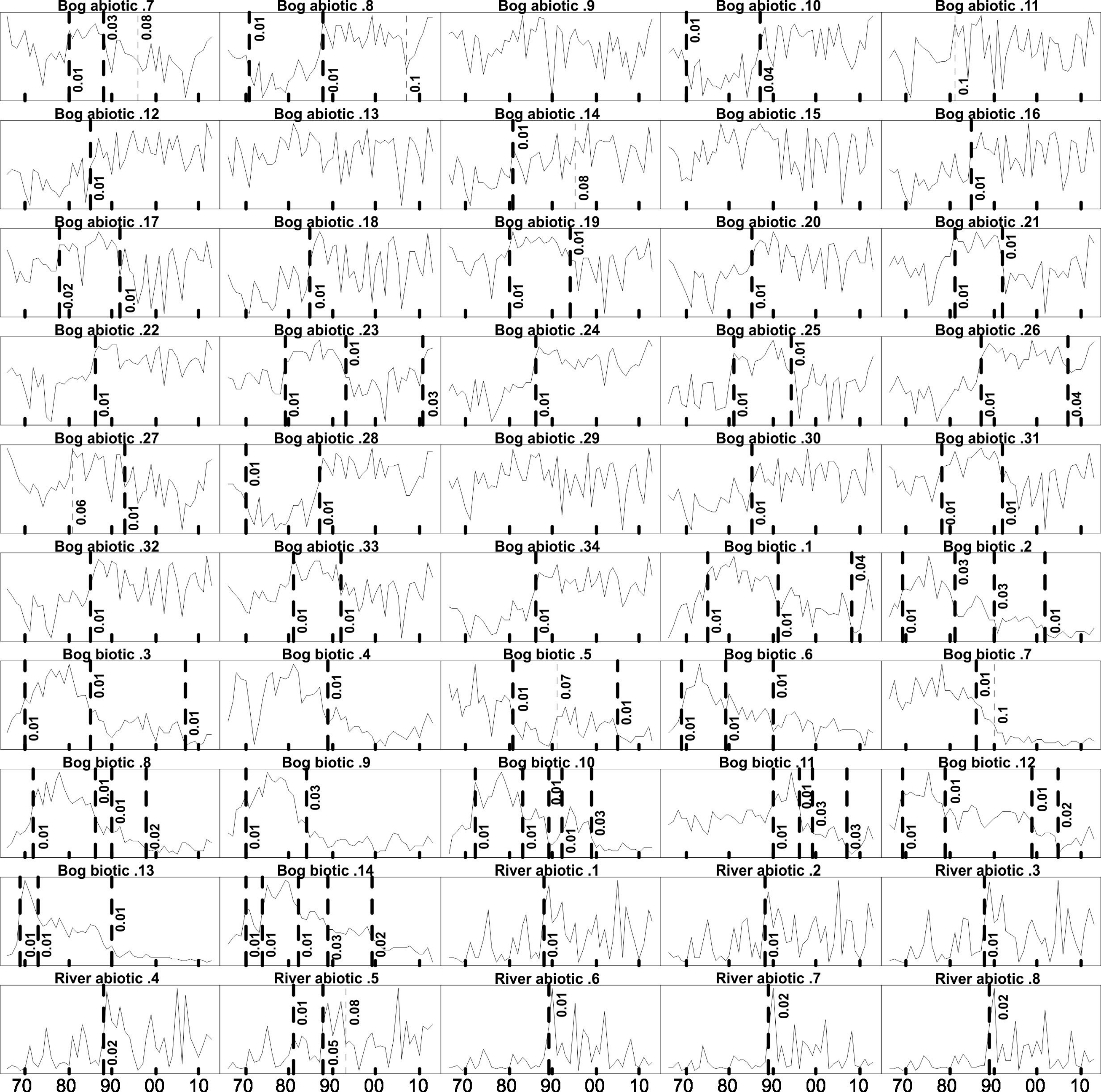

Bog abiotic .7
Bog abiotic .8
Bog abiotic .9
Bog abiotic .10
Bog abiotic .11
Bog abiotic .12
Bog abiotic .13
Bog abiotic .14
Bog abiotic .15
Bog abiotic .16
Bog abiotic .17
Bog abiotic .18
Bog abiotic .19
Bog abiotic .20
Bog abiotic .21
Bog abiotic .22
Bog abiotic .23
Bog abiotic .24
Bog abiotic .25
Bog abiotic .26
Bog abiotic .27
Bog abiotic .28
Bog abiotic .29
Bog abiotic .30
Bog abiotic .31
Bog abiotic .32
Bog abiotic .33
Bog abiotic .34
Bog biotic .1
Bog biotic .2
Bog biotic .3
Bog biotic .4
Bog biotic .5
Bog biotic .6
Bog biotic .7
Bog biotic .8
Bog biotic .9
Bog biotic .10
Bog biotic .11
Bog biotic .12
Bog biotic .13
Bog biotic .14
River abiotic .1
River abiotic .2
River abiotic .3
River abiotic .4
River abiotic .5
River abiotic .6
River abiotic .7
River abiotic .8
70 80 90 00 10

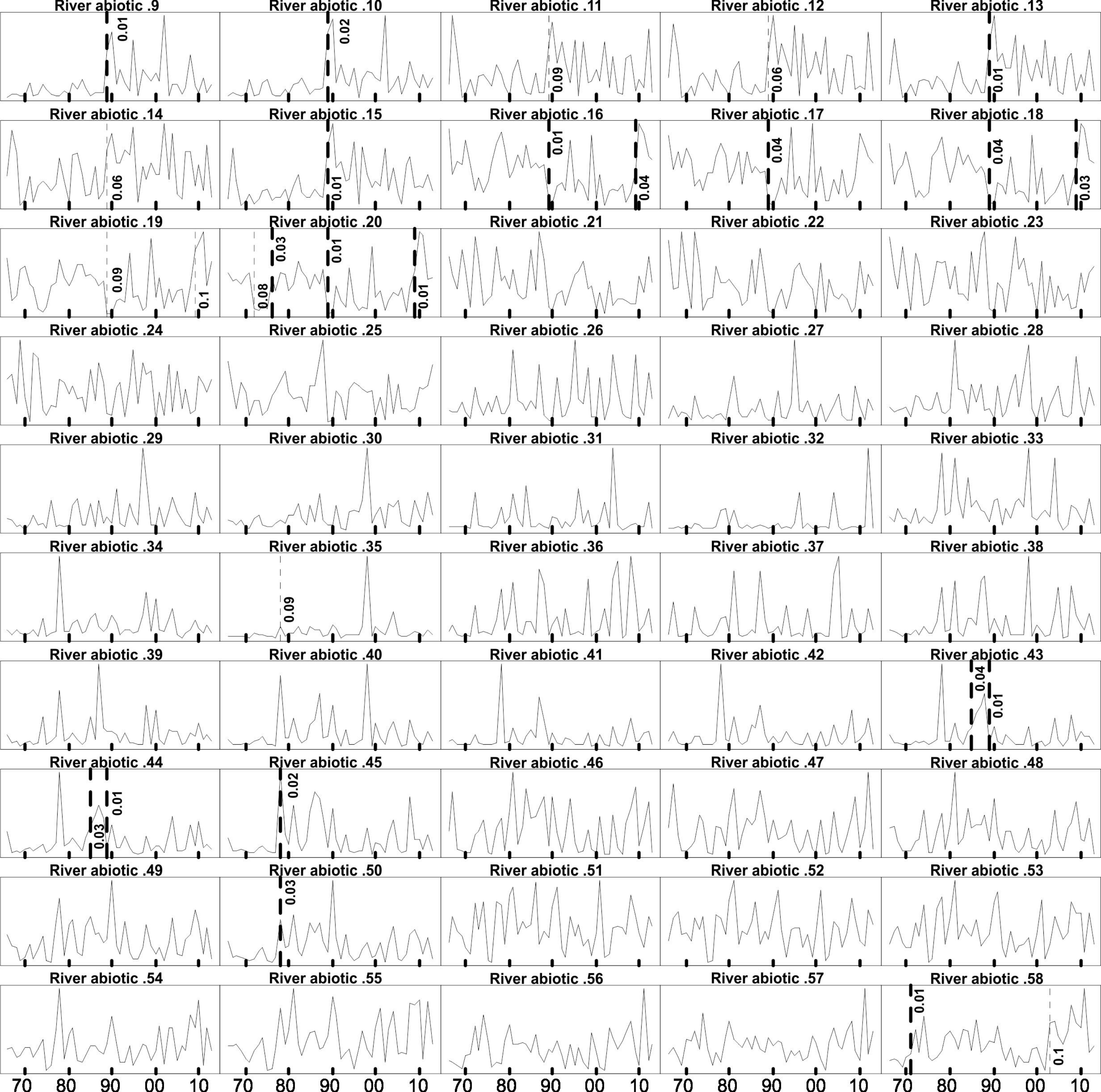
River abiotic .9
0.01
River abiotic .10
0.02
River abiotic .11
0.09
River abiotic .12
0.06
River abiotic .13
0.01
River abiotic .14
0.06
River abiotic .15
0.01
River abiotic .16
0.01
0.04
River abiotic .17
0.04
River abiotic .18
0.04
0.03
River abiotic .19
0.09
0.1
River abiotic .20
0.03
0.01
0.08
0.01
River abiotic .21
River abiotic .22
River abiotic .23
River abiotic .24
River abiotic .25
River abiotic .26
River abiotic .27
River abiotic .28
River abiotic .29
River abiotic .30
River abiotic .31
River abiotic .32
River abiotic .33
River abiotic .34
River abiotic .35
0.09
River abiotic .36
River abiotic .37
River abiotic .38
River abiotic .39
River abiotic .40
River abiotic .41
River abiotic .42
River abiotic .43
0.04
0.01
River abiotic .44
0.01
0.03
River abiotic .45
0.02
River abiotic .46
River abiotic .47
River abiotic .48
River abiotic .49
River abiotic .50
0.03
River abiotic .51
River abiotic .52
River abiotic .53
River abiotic .54
River abiotic .55
River abiotic .56
River abiotic .57
River abiotic .58
0.01
0.1
70 80 90 00 10
70 80 90 00 10
70 80 90 00 10
70 80 90 00 10
70 80 90 00 10

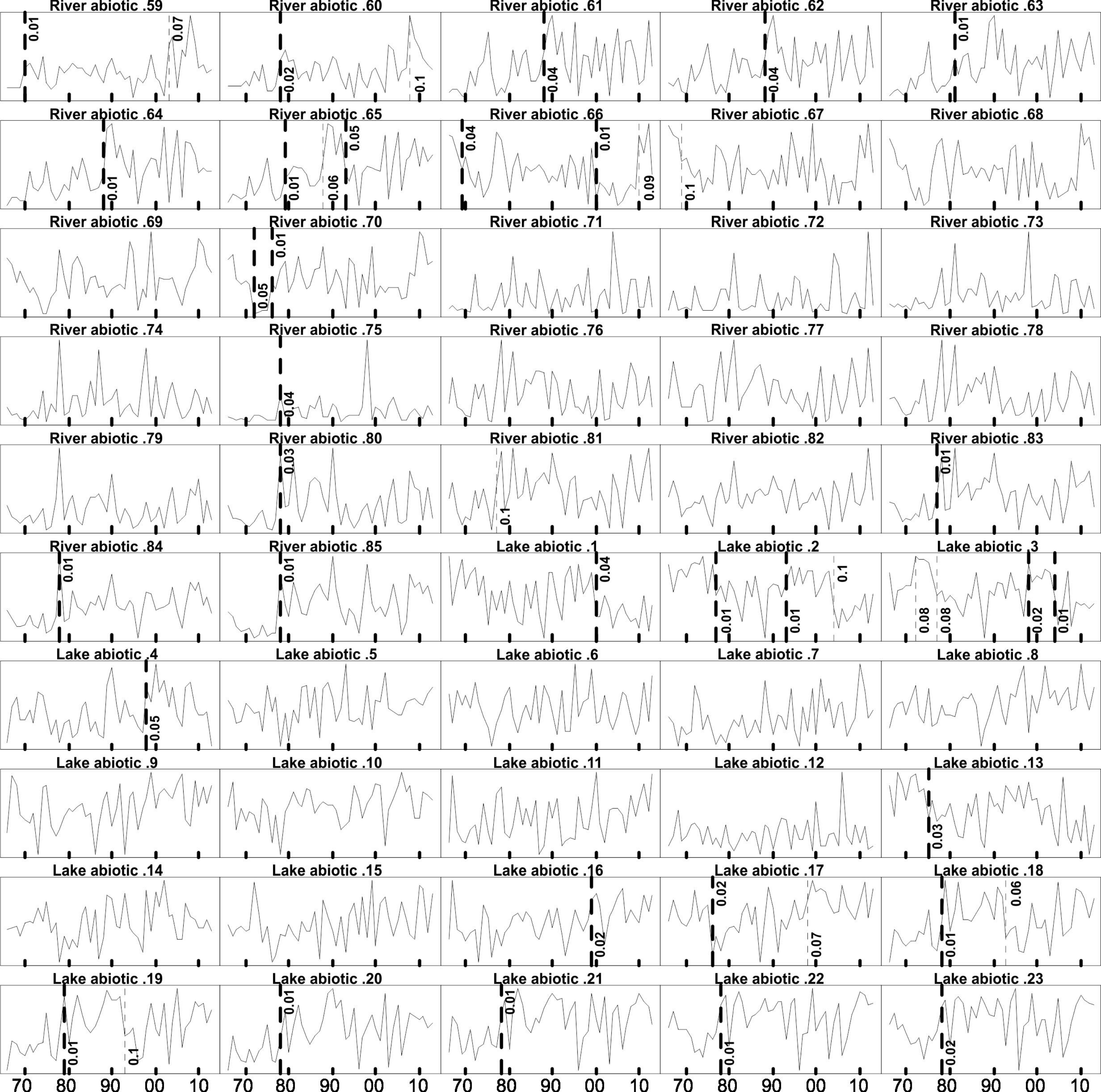
River abiotic .59
0.01
0.07
River abiotic .60
0.02
0.1
River abiotic .61
0.04
River abiotic .62
0.04
River abiotic .63
0.01
River abiotic .64
0.01
River abiotic .65
0.01
0.06
0.05
River abiotic .66
0.04
0.01
0.09
River abiotic .67
0.1
River abiotic .68
River abiotic .69
River abiotic .70
0.05
0.01
River abiotic .71
River abiotic .72
River abiotic .73
River abiotic .74
River abiotic .75
0.04
River abiotic .76
River abiotic .77
River abiotic .78
River abiotic .79
River abiotic .80
0.03
River abiotic .81
0.1
River abiotic .82
River abiotic .83
0.01
River abiotic .84
0.01
River abiotic .85
0.01
Lake abiotic .1
0.04
Lake abiotic .2
0.01
0.01
0.1
Lake abiotic .3
0.08
0.08
0.02
0.01
Lake abiotic .4
0.05
Lake abiotic .5
Lake abiotic .6
Lake abiotic .7
Lake abiotic .8
Lake abiotic .9
Lake abiotic .10
Lake abiotic .11
Lake abiotic .12
Lake abiotic .13
0.03
Lake abiotic .14
Lake abiotic .15
Lake abiotic .16
0.02
Lake abiotic .17
0.02
0.07
Lake abiotic .18
0.01
0.06
Lake abiotic .19
0.01
0.1
Lake abiotic .20
0.01
Lake abiotic .21
0.01
Lake abiotic .22
0.01
Lake abiotic .23
0.02
70 80 90 00 10

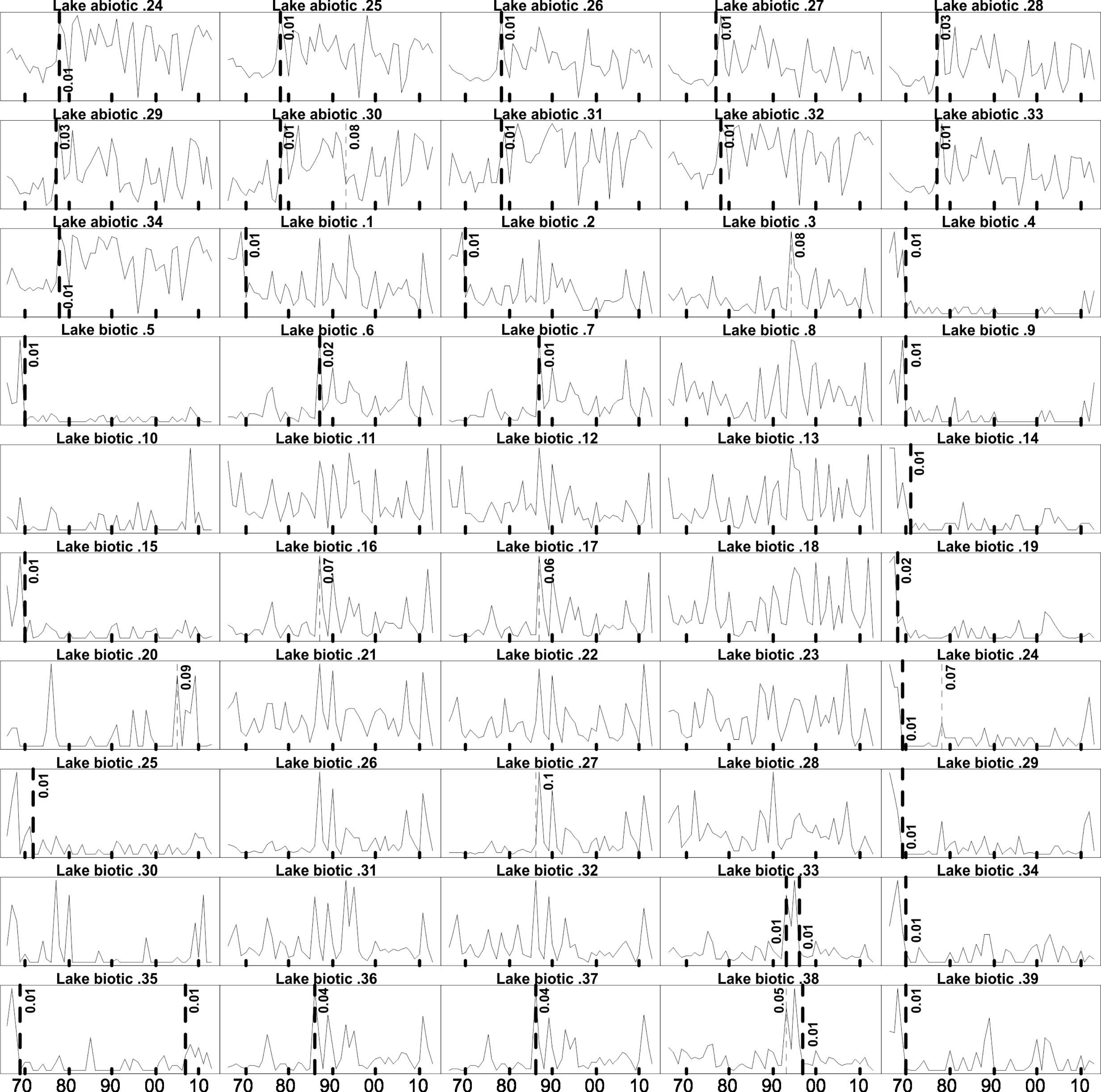

Lake abiotic .24
0.01
Lake abiotic .25
0.01
Lake abiotic .26
0.01
Lake abiotic .27
0.01
Lake abiotic .28
0.03
Lake abiotic .29
0.03
Lake abiotic .30
0.01
0.08
Lake abiotic .31
0.01
Lake abiotic .32
0.01
Lake abiotic .33
0.01
Lake abiotic .34
0.01
Lake biotic .1
0.01
Lake biotic .2
0.01
Lake biotic .3
0.08
Lake biotic .4
0.01
Lake biotic .5
0.01
Lake biotic .6
0.02
Lake biotic .7
0.01
Lake biotic .8
Lake biotic .9
0.01
Lake biotic .10
Lake biotic .11
Lake biotic .12
Lake biotic .13
Lake biotic .14
0.01
Lake biotic .15
0.01
Lake biotic .16
0.07
Lake biotic .17
0.06
Lake biotic .18
Lake biotic .19
0.02
Lake biotic .20
0.09
Lake biotic .21
Lake biotic .22
Lake biotic .23
Lake biotic .24
0.07
0.01
Lake biotic .25
0.01
Lake biotic .26
Lake biotic .27
0.1
Lake biotic .28
Lake biotic .29
0.01
Lake biotic .30
Lake biotic .31
Lake biotic .32
Lake biotic .33
0.01
0.01
Lake biotic .34
0.01
Lake biotic .35
0.01
0.01
Lake biotic .36
0.04
Lake biotic .37
0.04
Lake biotic .38
0.05
0.01
Lake biotic .39
0.01
70 80 90 00 10

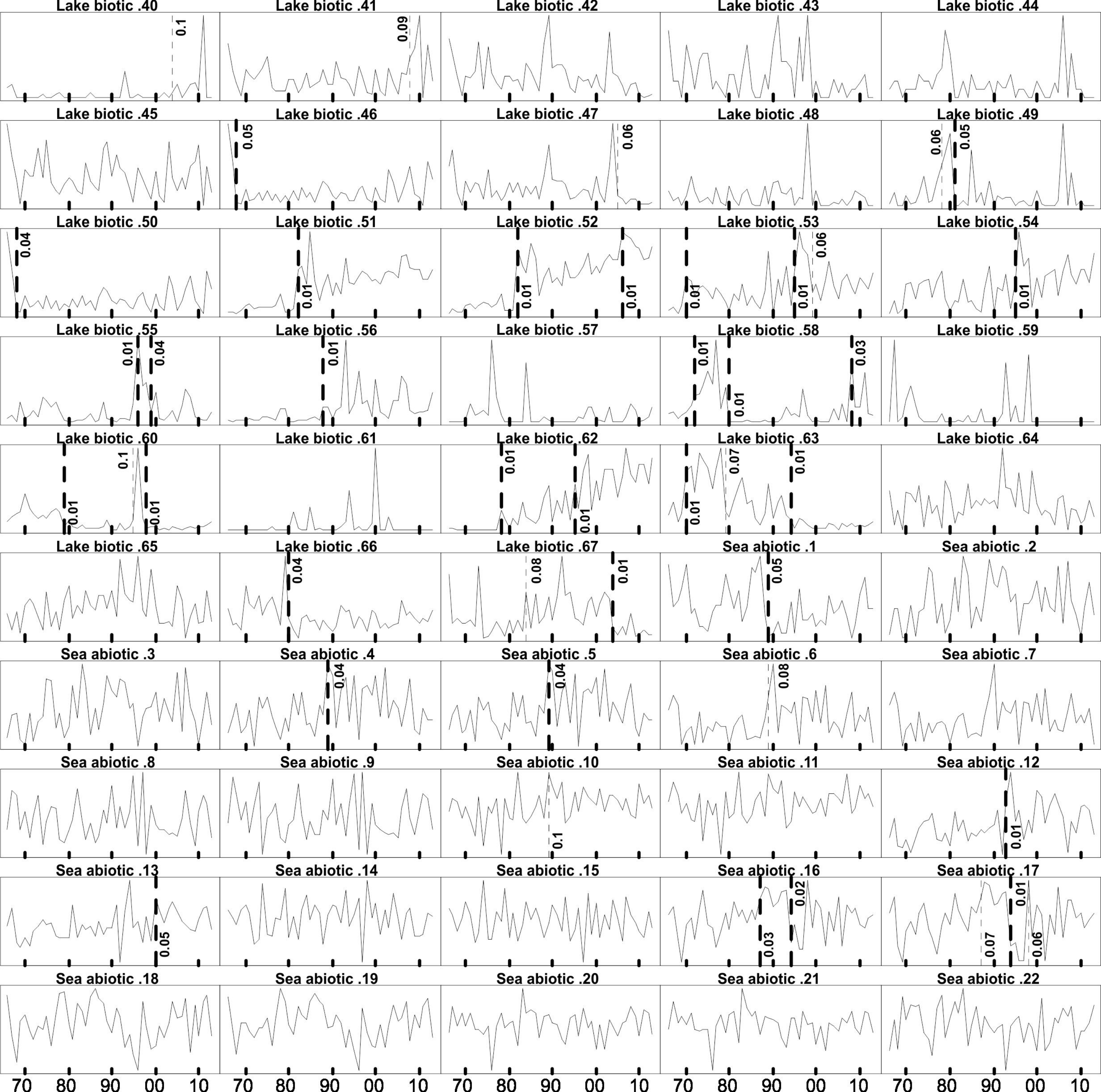
Lake biotic .40
0.1
Lake biotic .41
0.09
Lake biotic .42
Lake biotic .43
Lake biotic .44
Lake biotic .45
Lake biotic .46
0.05
Lake biotic .47
0.06
Lake biotic .48
Lake biotic .49
0.06
0.05
Lake biotic .50
0.04
Lake biotic .51
0.01
Lake biotic .52
0.01
0.01
Lake biotic .53
0.01
0.01
0.06
Lake biotic .54
0.01
Lake biotic .55
0.01
0.04
Lake biotic .56
0.01
Lake biotic .57
Lake biotic .58
0.01
0.01
0.03
Lake biotic .59
Lake biotic .60
0.01
0.1
0.01
Lake biotic .61
Lake biotic .62
0.01
0.01
Lake biotic .63
0.01
0.07
0.01
Lake biotic .64
Lake biotic .65
Lake biotic .66
0.04
Lake biotic .67
0.08
0.01
Sea abiotic .1
0.05
Sea abiotic .2
Sea abiotic .3
Sea abiotic .4
0.04
Sea abiotic .5
0.04
Sea abiotic .6
0.08
Sea abiotic .7
Sea abiotic .8
Sea abiotic .9
Sea abiotic .10
0.1
Sea abiotic .11
Sea abiotic .12
0.01
Sea abiotic .13
0.05
Sea abiotic .14
Sea abiotic .15
Sea abiotic .16
0.03
0.02
Sea abiotic .17
0.07
0.01
0.06
Sea abiotic .18
Sea abiotic .19
Sea abiotic .20
Sea abiotic .21
Sea abiotic .22
70 80 90 00 10

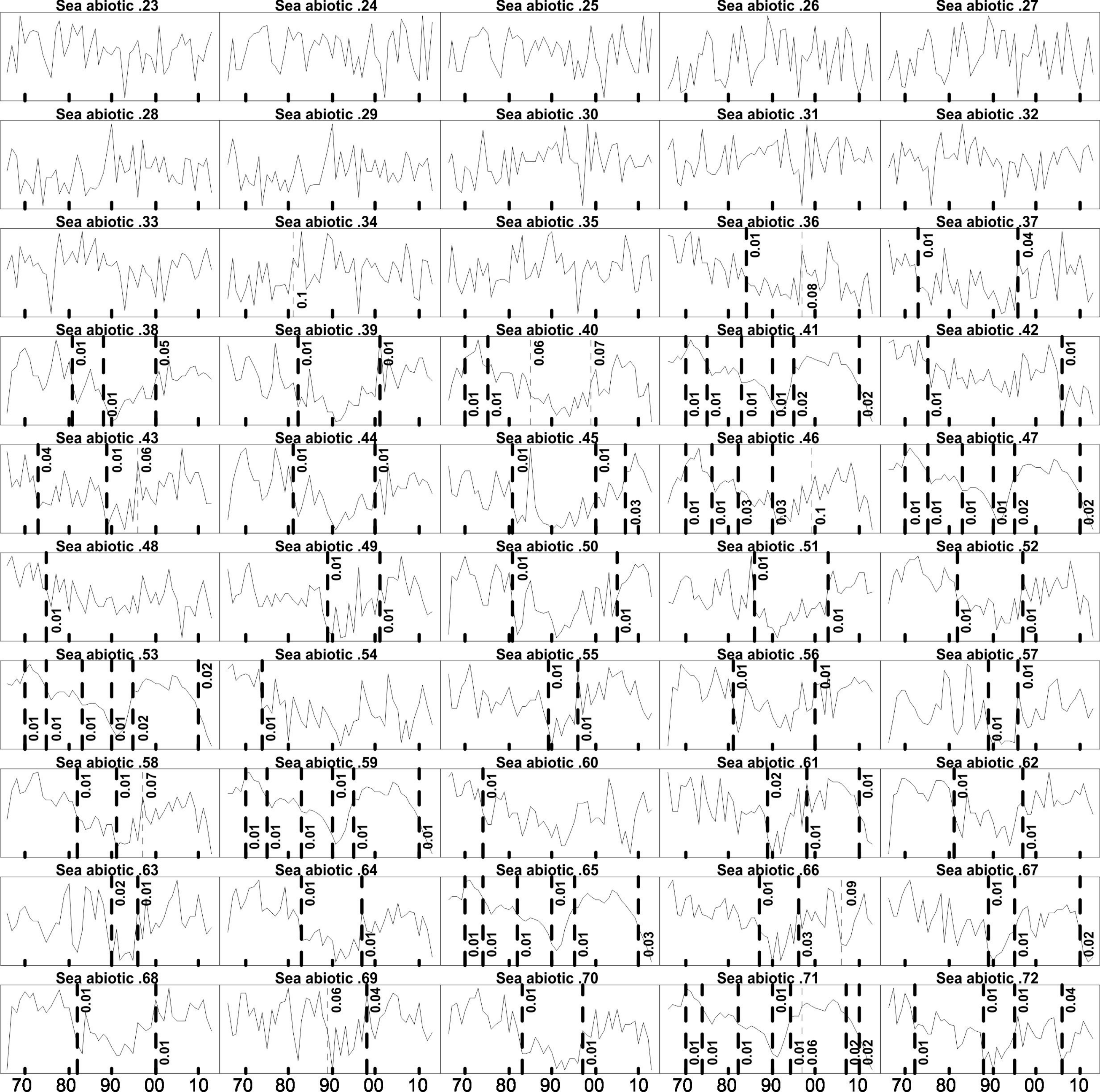
Sea abiotic .23
Sea abiotic .24
Sea abiotic .25
Sea abiotic .26
Sea abiotic .27
Sea abiotic .28
Sea abiotic .29
Sea abiotic .30
Sea abiotic .31
Sea abiotic .32
Sea abiotic .33
Sea abiotic .34
Sea abiotic .35
Sea abiotic .36
Sea abiotic .37
Sea abiotic .38
Sea abiotic .39
Sea abiotic .40
Sea abiotic .41
Sea abiotic .42
Sea abiotic .43
Sea abiotic .44
Sea abiotic .45
Sea abiotic .46
Sea abiotic .47
Sea abiotic .48
Sea abiotic .49
Sea abiotic .50
Sea abiotic .51
Sea abiotic .52
Sea abiotic .53
Sea abiotic .54
Sea abiotic .55
Sea abiotic .56
Sea abiotic .57
Sea abiotic .58
Sea abiotic .59
Sea abiotic .60
Sea abiotic .61
Sea abiotic .62
Sea abiotic .63
Sea abiotic .64
Sea abiotic .65
Sea abiotic .66
Sea abiotic .67
Sea abiotic .68
Sea abiotic .69
Sea abiotic .70
Sea abiotic .71
Sea abiotic .72
70
80
90
00
10

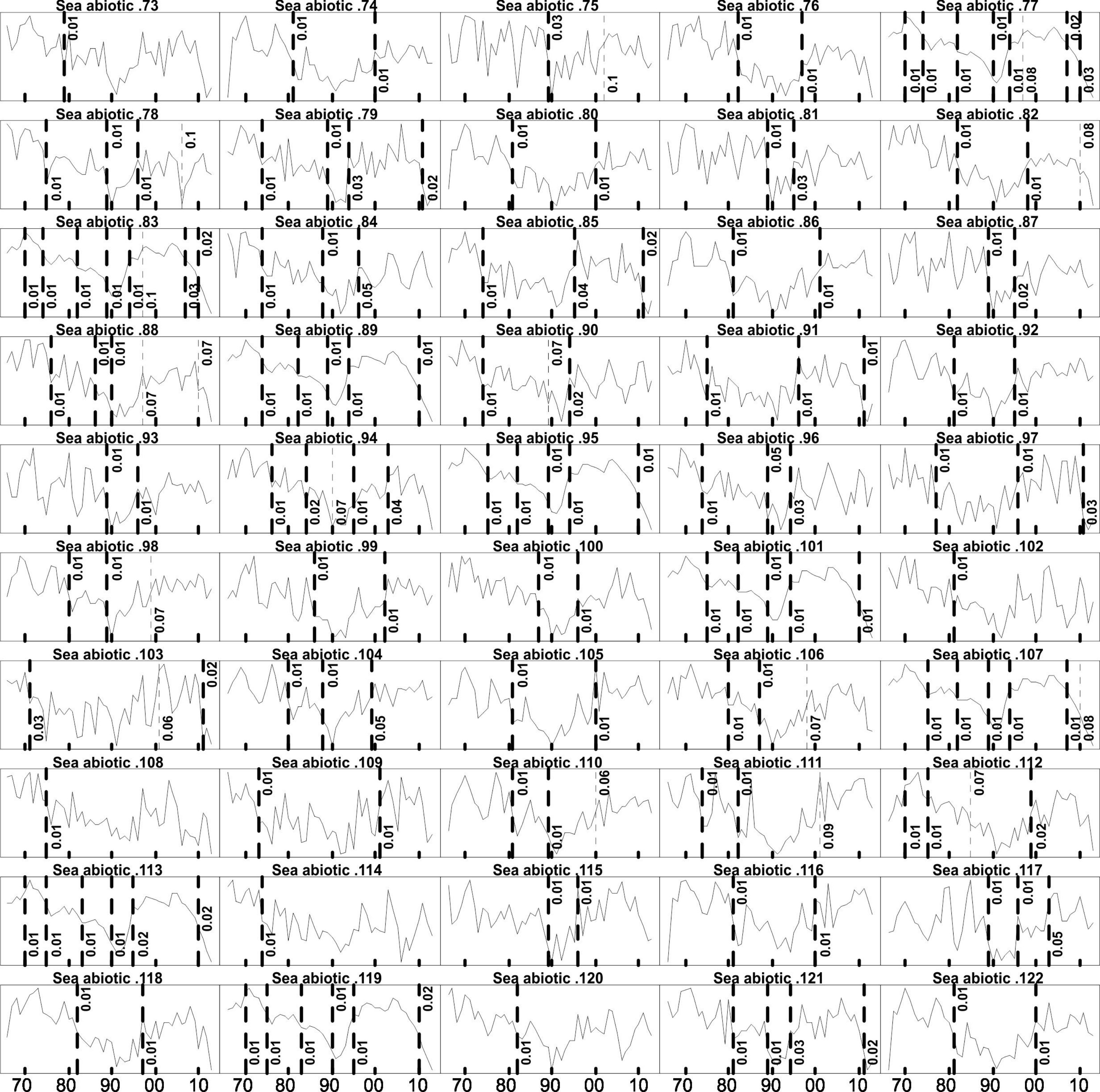

Sea abiotic .73
Sea abiotic .74
Sea abiotic .75
Sea abiotic .76
Sea abiotic .77
Sea abiotic .78
Sea abiotic .79
Sea abiotic .80
Sea abiotic .81
Sea abiotic .82
Sea abiotic .83
Sea abiotic .84
Sea abiotic .85
Sea abiotic .86
Sea abiotic .87
Sea abiotic .88
Sea abiotic .89
Sea abiotic .90
Sea abiotic .91
Sea abiotic .92
Sea abiotic .93
Sea abiotic .94
Sea abiotic .95
Sea abiotic .96
Sea abiotic .97
Sea abiotic .98
Sea abiotic .99
Sea abiotic .100
Sea abiotic .101
Sea abiotic .102
Sea abiotic .103
Sea abiotic .104
Sea abiotic .105
Sea abiotic .106
Sea abiotic .107
Sea abiotic .108
Sea abiotic .109
Sea abiotic .110
Sea abiotic .111
Sea abiotic .112
Sea abiotic .113
Sea abiotic .114
Sea abiotic .115
Sea abiotic .116
Sea abiotic .117
Sea abiotic .118
Sea abiotic .119
Sea abiotic .120
Sea abiotic .121
Sea abiotic .122
70 80 90 00 10

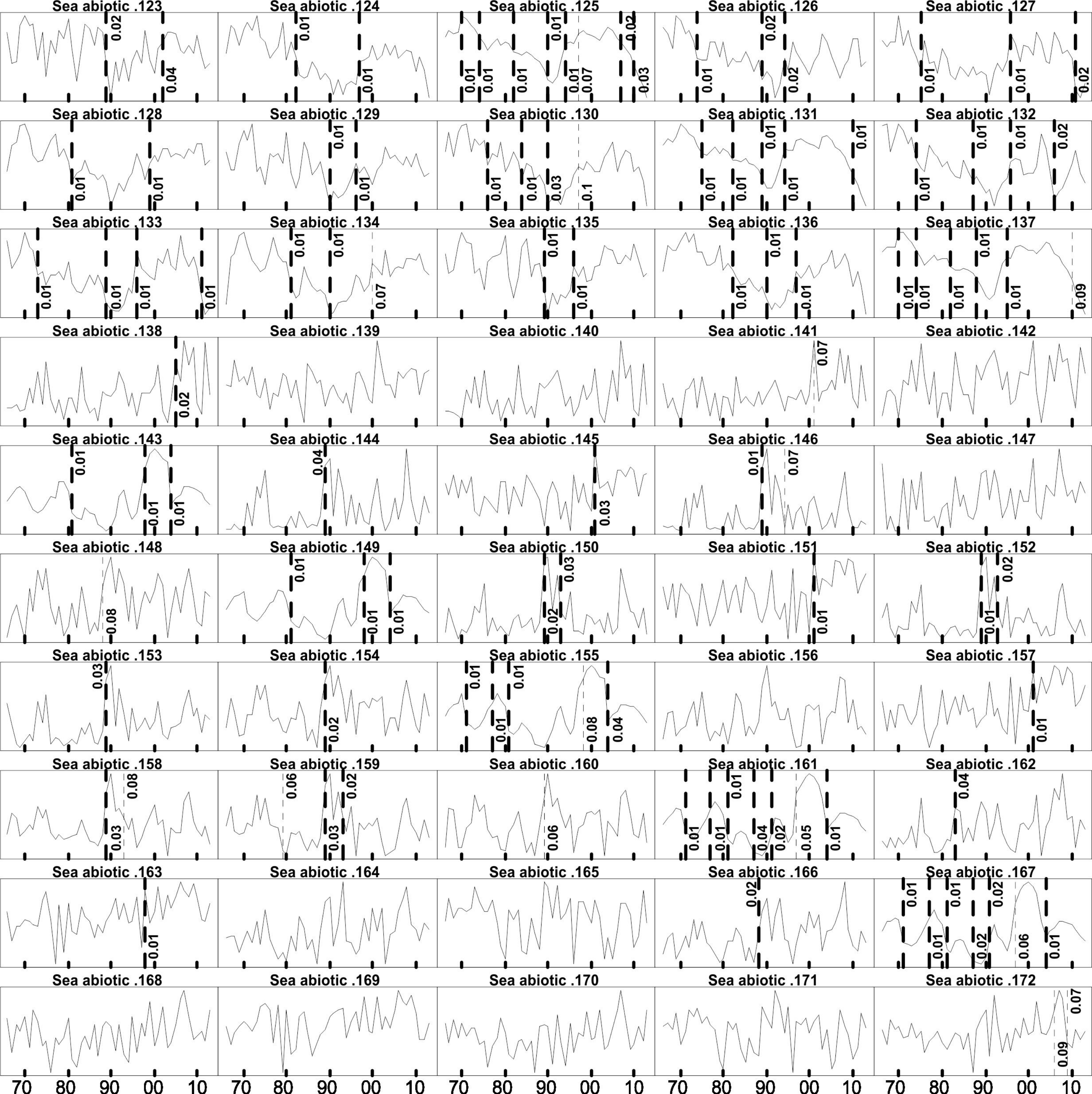
Sea abiotic .123
Sea abiotic .124
Sea abiotic .125
Sea abiotic .126
Sea abiotic .127
Sea abiotic .128
Sea abiotic .129
Sea abiotic .130
Sea abiotic .131
Sea abiotic .132
Sea abiotic .133
Sea abiotic .134
Sea abiotic .135
Sea abiotic .136
Sea abiotic .137
Sea abiotic .138
Sea abiotic .139
Sea abiotic .140
Sea abiotic .141
Sea abiotic .142
Sea abiotic .143
Sea abiotic .144
Sea abiotic .145
Sea abiotic .146
Sea abiotic .147
Sea abiotic .148
Sea abiotic .149
Sea abiotic .150
Sea abiotic .151
Sea abiotic .152
Sea abiotic .153
Sea abiotic .154
Sea abiotic .155
Sea abiotic .156
Sea abiotic .157
Sea abiotic .158
Sea abiotic .159
Sea abiotic .160
Sea abiotic .161
Sea abiotic .162
Sea abiotic .163
Sea abiotic .164
Sea abiotic .165
Sea abiotic .166
Sea abiotic .167
Sea abiotic .168
Sea abiotic .169
Sea abiotic .170
Sea abiotic .171
Sea abiotic .172
70 80 90 00 10

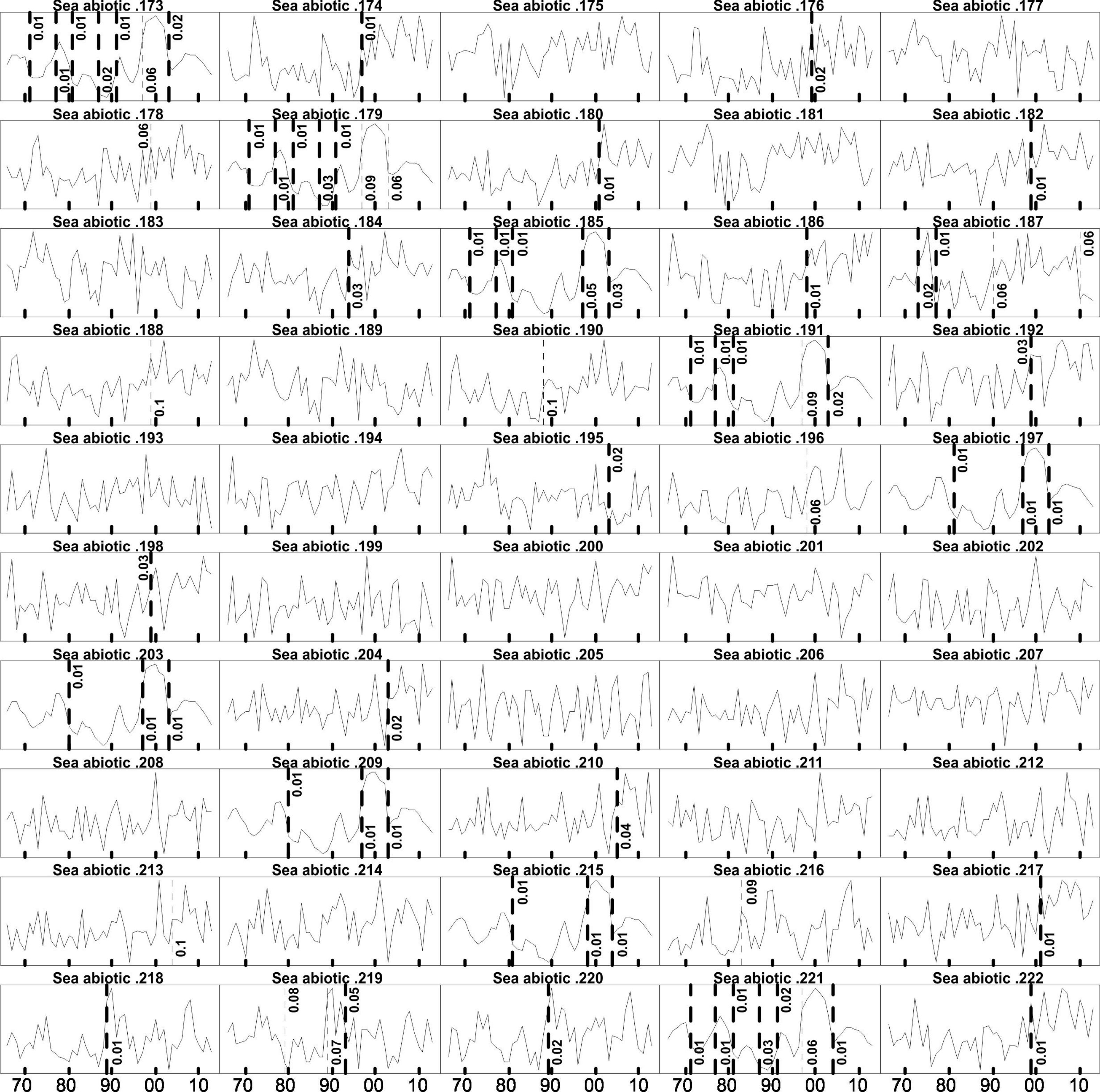
Sea abiotic .173
Sea abiotic .174
Sea abiotic .175
Sea abiotic .176
Sea abiotic .177
Sea abiotic .178
Sea abiotic .179
Sea abiotic .180
Sea abiotic .181
Sea abiotic .182
Sea abiotic .183
Sea abiotic .184
Sea abiotic .185
Sea abiotic .186
Sea abiotic .187
Sea abiotic .188
Sea abiotic .189
Sea abiotic .190
Sea abiotic .191
Sea abiotic .192
Sea abiotic .193
Sea abiotic .194
Sea abiotic .195
Sea abiotic .196
Sea abiotic .197
Sea abiotic .198
Sea abiotic .199
Sea abiotic .200
Sea abiotic .201
Sea abiotic .202
Sea abiotic .203
Sea abiotic .204
Sea abiotic .205
Sea abiotic .206
Sea abiotic .207
Sea abiotic .208
Sea abiotic .209
Sea abiotic .210
Sea abiotic .211
Sea abiotic .212
Sea abiotic .213
Sea abiotic .214
Sea abiotic .215
Sea abiotic .216
Sea abiotic .217
Sea abiotic .218
Sea abiotic .219
Sea abiotic .220
Sea abiotic .221
Sea abiotic .222
70 80 90 00 10

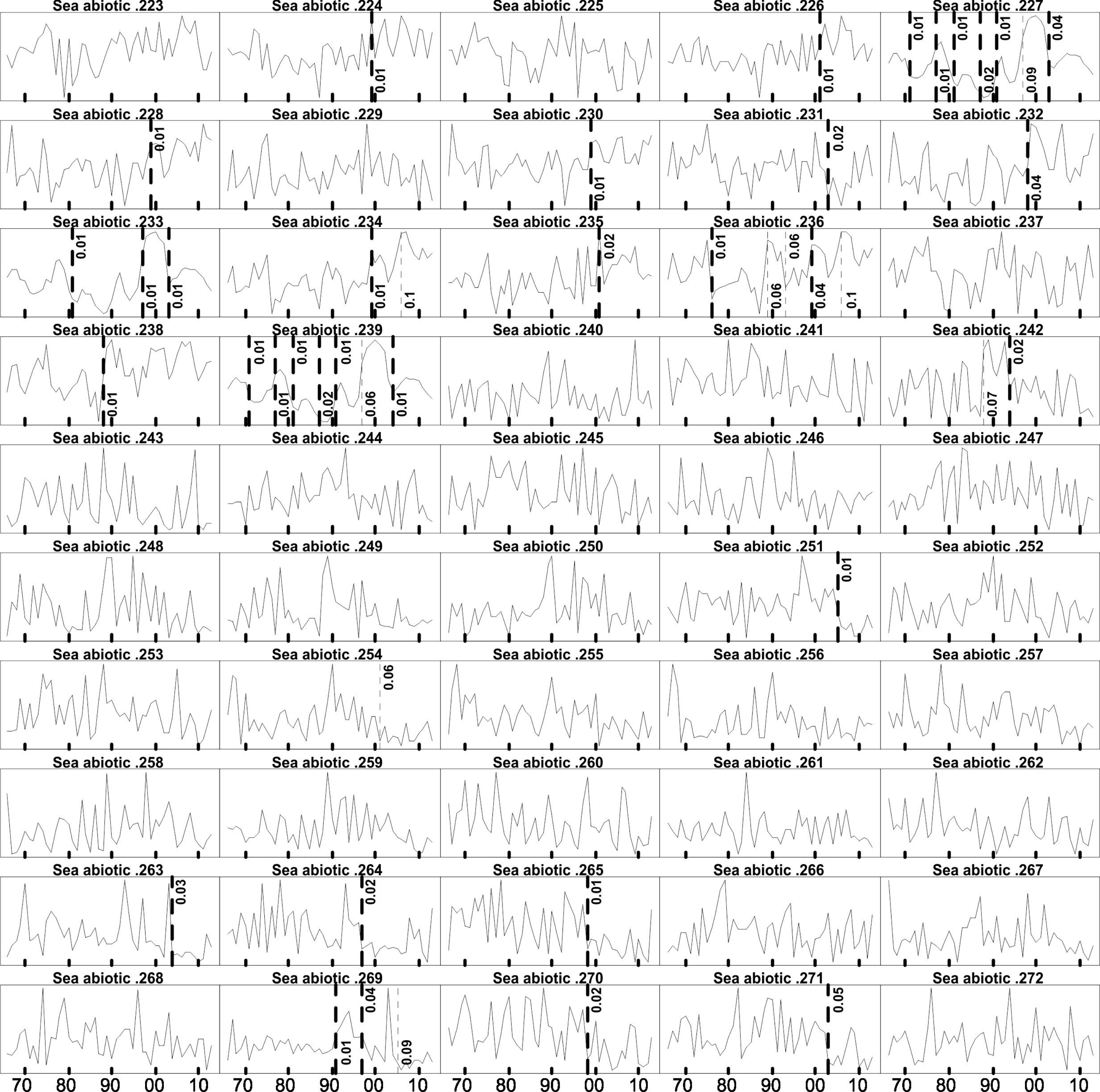

Sea abiotic .223
Sea abiotic .224
0.01
Sea abiotic .225
Sea abiotic .226
0.01
Sea abiotic .227
0.01
0.01
0.01
0.04
0.01
0.02
0.09
Sea abiotic .228
0.01
Sea abiotic .229
Sea abiotic .230
0.01
Sea abiotic .231
0.02
Sea abiotic .232
0.04
Sea abiotic .233
0.01
0.01
0.01
Sea abiotic .234
0.01
0.1
Sea abiotic .235
0.02
Sea abiotic .236
0.01
0.06
0.06
0.04
0.1
Sea abiotic .237
Sea abiotic .238
0.01
Sea abiotic .239
0.01
0.01
0.01
0.01
0.02
0.06
0.01
Sea abiotic .240
Sea abiotic .241
Sea abiotic .242
0.02
0.07
Sea abiotic .243
Sea abiotic .244
Sea abiotic .245
Sea abiotic .246
Sea abiotic .247
Sea abiotic .248
Sea abiotic .249
Sea abiotic .250
Sea abiotic .251
0.01
Sea abiotic .252
Sea abiotic .253
Sea abiotic .254
0.06
Sea abiotic .255
Sea abiotic .256
Sea abiotic .257
Sea abiotic .258
Sea abiotic .259
Sea abiotic .260
Sea abiotic .261
Sea abiotic .262
Sea abiotic .263
0.03
Sea abiotic .264
0.02
Sea abiotic .265
0.01
Sea abiotic .266
Sea abiotic .267
Sea abiotic .268
Sea abiotic .269
0.04
0.01
0.09
Sea abiotic .270
0.02
Sea abiotic .271
0.05
Sea abiotic .272
70 80 90 00 10
70 80 90 00 10
70 80 90 00 10
70 80 90 00 10
70 80 90 00 10

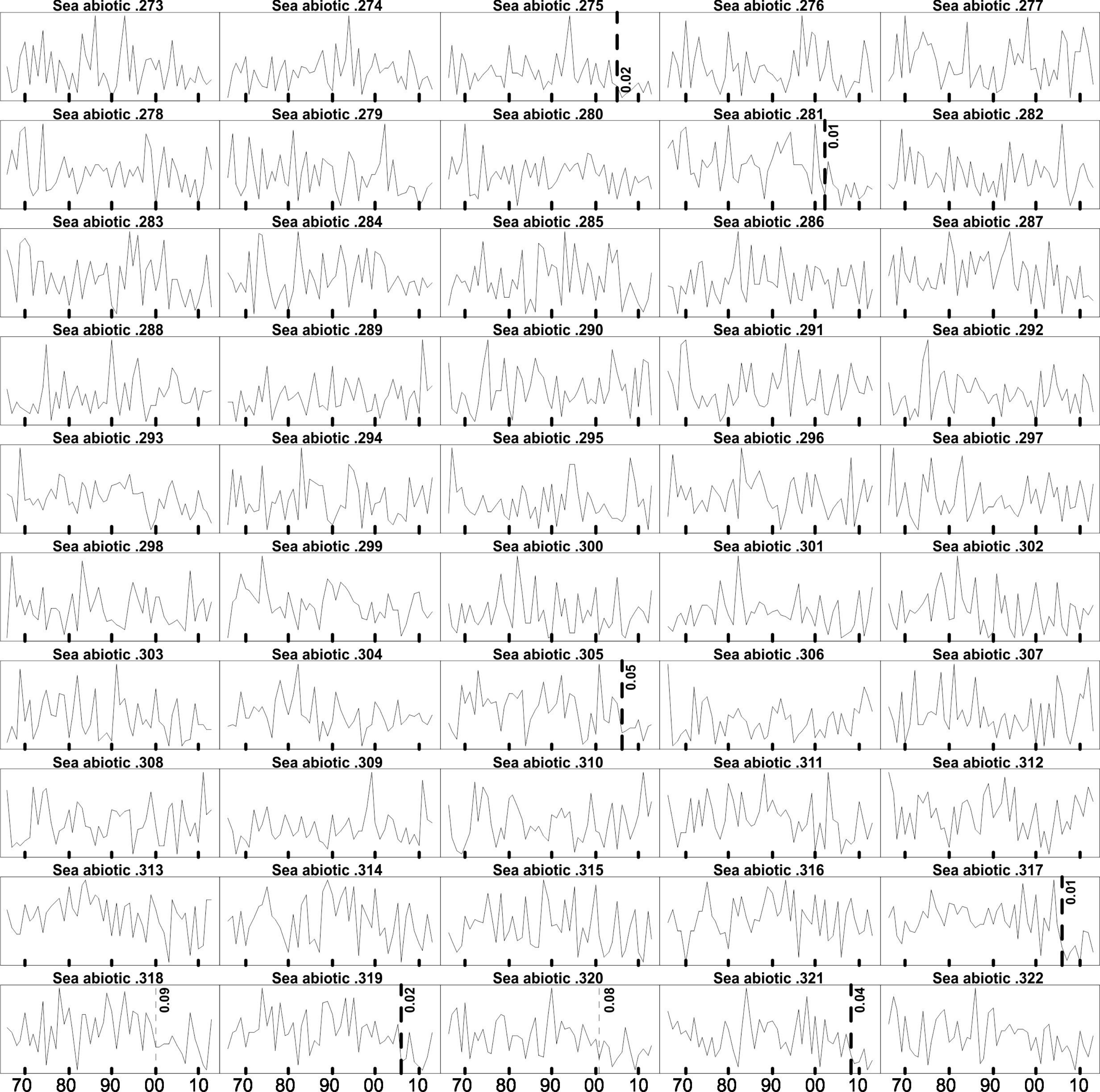

Sea abiotic .273
Sea abiotic .274
Sea abiotic .275
0.02
Sea abiotic .276
Sea abiotic .277
Sea abiotic .278
Sea abiotic .279
Sea abiotic .280
Sea abiotic .281
0.01
Sea abiotic .282
Sea abiotic .283
Sea abiotic .284
Sea abiotic .285
Sea abiotic .286
Sea abiotic .287
Sea abiotic .288
Sea abiotic .289
Sea abiotic .290
Sea abiotic .291
Sea abiotic .292
Sea abiotic .293
Sea abiotic .294
Sea abiotic .295
Sea abiotic .296
Sea abiotic .297
Sea abiotic .298
Sea abiotic .299
Sea abiotic .300
Sea abiotic .301
Sea abiotic .302
Sea abiotic .303
Sea abiotic .304
Sea abiotic .305
0.05
Sea abiotic .306
Sea abiotic .307
Sea abiotic .308
Sea abiotic .309
Sea abiotic .310
Sea abiotic .311
Sea abiotic .312
Sea abiotic .313
Sea abiotic .314
Sea abiotic .315
Sea abiotic .316
Sea abiotic .317
0.01
Sea abiotic .318
0.09
Sea abiotic .319
0.02
Sea abiotic .320
0.08
Sea abiotic .321
0.04
Sea abiotic .322
70 80 90 00 10

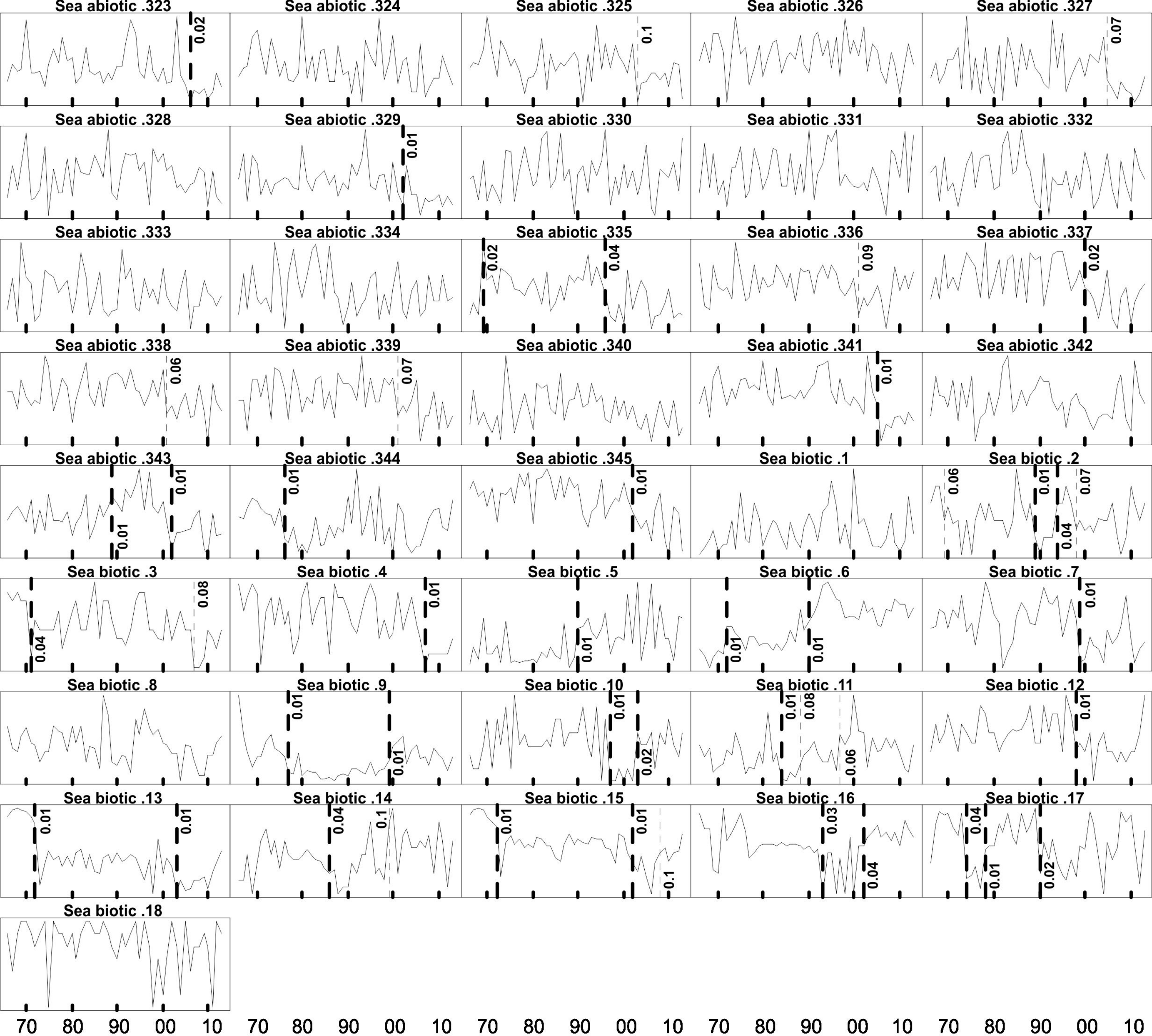
Sea abiotic .323
Sea abiotic .324
Sea abiotic .325
Sea abiotic .326
Sea abiotic .327
Sea abiotic .328
Sea abiotic .329
Sea abiotic .330
Sea abiotic .331
Sea abiotic .332
Sea abiotic .333
Sea abiotic .334
Sea abiotic .335
Sea abiotic .336
Sea abiotic .337
Sea abiotic .338
Sea abiotic .339
Sea abiotic .340
Sea abiotic .341
Sea abiotic .342
Sea abiotic .343
Sea abiotic .344
Sea abiotic .345
Sea biotic .1
Sea biotic .2
Sea biotic .3
Sea biotic .4
Sea biotic .5
Sea biotic .6
Sea biotic .7
Sea biotic .8
Sea biotic .9
Sea biotic .10
Sea biotic .11
Sea biotic .12
Sea biotic .13
Sea biotic .14
Sea biotic .15
Sea biotic .16
Sea biotic .17
Sea biotic .18
70 80 90 00 10
70 80 90 00 10
70 80 90 00 10
70 80 90 00 10
70 80 90 00 10

S1 Table References

Agasild H, Zingel P, Tõnno I, Haberman J, Nõges T. Contribution of different zooplankton groups in grazing on phytoplankton in shallow eutrophic Lake Võrtsjärv (Estonia). Shallow Lakes in a Changing World. Dordrecht: Springer; 2007.p. 167−177.

Arula T, Gröger J, Ojaveer H, Simm M. Shifts in the spring herring (*Clupea harengus membras*) larvae and related environment in the eastern Baltic Sea over the past 50 years. PLoS ONE. 2014; 9(3): e91304.

Estonian Weather Service, a [Internet]. Tallinn: Riigi Ilmateenistus; c2016 [cited 2018 Sept 18]. Available from: www.ilmateenistus.ee

Estonian Weather Service, b [Internet]. Tallinn: Ajaloolised vaatlusandmed; c2016 [cited 2018 Sept 18]. Available from: http://www.ilmateenistus.ee/siseveed/ajaloolised-vaatlusandmed/

Heinsalu A, Luup H, Alliksaar T, Nõges P, Nõges T. Water level changes in a large shallow lake as reflected by the plankton: periphyton-ratio of sedimentary diatoms. European Large Lakes Ecosystem changes and their ecological and socioeconomic impacts. Dordrecht: Springer; 2007. p. 23−30.

Hurrell JW. Decadal trends in the North Atlantic Oscillation and relationships to regional temperature and precipitation. Science. 1995; 269: 676−679.

Hurrell JW, Kushnir Y, Ottersen G, Visbeck M. An Overview of the North Atlantic Oscillation. In: Hurrell JW, Kushnir Y, Ottersen G, Visbeck M, editors. The North Atlantic Oscillation: Climate Significance and Environmental Impact. Washington: AGU Publications; 2013. p. 1−35.

ICES. Report of the Baltic Fisheries Assessment Working Group (WGBFAS); 2015 April 14−21; Copenhagen, Denmark: ICES HQ, ICES CM 2015/ACOM:10; 2015. p. 1−806.

Jones PD, Jónsson T, Wheeler D. Extension to the North Atlantic Oscillation using early instrumental pressure observations from Gibraltar and South-West Iceland. Int J Climatol. 1997; 17:1433−1450.

Järvet A. Hydrology of Lake Võrtsjärv. In: Haberman J, Pihu E, Raukas A, editors. Lake Võrtsjärv Tallinn: Estonian Encyclopaedia Publishers; 2004. p. 105−139.

Kangur K, Timm H, Timm T. Zoobenthos. In: Haberman J, Pihu E, Raukas A, editors. Lake Võrtsjärv, 2004. p. 265−280.

Kont A, Endjärv E, Jaagus J, Lode E, Orviku K, Ratas U, Rivis R, Suursaar Ü, Tõnisson H. Impact of climate change on Estonian coastal and inland wetlands – a summary with new results. Boreal Environ Res. 2007; 12: 653−671.

Kumari M, Kangur K, Haldna M. Variation of macrozoobenthos communities in the reed Phragmites australis belt of two large shallow lakes. Proc Estonian Acad Sci Biol Ecol. 2007; 56(2): 141−153.

Laur K, Ojaveer H, Simm M, Klais R. Multidecadal dynamics of larval gobies *Pomatoschistus* spp. in response to environmental variability in a shallow temperate bay. Estuar Coast Shelf Sci. 2014; 136: 112−118.

Maljutenko I, Raudsepp U. Validation of GETM model simulated long-term salinity fields in the pathway of saltwater transport in response to the Major Baltic Inflows in the Baltic Sea. 2014 IEEE/OES Baltic International Symposium (BALTIC), J. Carroll, Ed., Tallinn, IEEE/OES, 23–31. 2014

Männik A, Zirk M, Rõõm R, Luhamaa A. Climate parameters of Estonia and the Baltic Sea region derived from the high-resolution reanalysis database BaltAn65+. Theor Appl Climatol. 2015; 122(1-2): 19−34.

Nõges P, Nõges T. Weak trends in ice phenology of Estonian large lakes despite significant warming trends. Hydrobiologia. 2014; 731(1): 5−18.

Nõges P, Mischke U, Laugaste R, Solimini AG. Analysis of changes over 44 years in the phytoplankton of Lake Võrtsjärv (Estonia): the effect of nutrients, climate and the investigator on phytoplankton-based water quality indices. Hydrobiologia. 2010; 646(1): 33−48.

Nõges P, Järvet A. Climate driven changes in the spawning of roach (*Rutilus rutilus* (L.)) and bream(*Abramis brama* (L.)) in the Estonian part of the Narva River basin. Boreal Environ Res. 2005; 10(1): 45−55.

Ojaveer E, Raid T, Suursaar Ü. On the assessment and management of local herring stocks in the Baltic Sea. In: Payne A, O'Brien C, Rogers S, editors. Management of Shared Fish Stocks. Oxford:Blackwell Publishing; 2004. p. 240–250.

Paal J, Jürjendal I, Suija A, Kull A. Impact of drainage on vegetation of transitional mires in Estonia. Mires and Peat. 2016; 18: 1−19.

Russak V, Kallis A. Eesti kiirguskliima teatmik (Handbook of Estonian Solar Radiation Climate). In: Tooming H, editor. Tallinn: OÜ Stilett Trükikoda; 2003. Estonian.

Suursaar Ü, Jaagus J, Tõnisson H. How to quantify long-term changes in coastal sea storminess? Estuar Coast Shelf Sci. 2015; 156: 31−41.

Timm T, Kangur K, Timm H, Timm V. Zoobenthos. Lake Peipsi. Flora and Fauna. Tallinn: Sulemees Publishers; 2001. p. 82−99.

Zingel P, Haberman J. A comparison of zooplankton densities and biomass in Lakes Peipsi and Võrtsjärv (Estonia): rotifers and crustaceans versus ciliates. European Large Lakes Ecosystem changes and their ecological and socioeconomic impacts. Dordrecht: Springer; 2007. p. 153−159.
