# Supplementary material for: Linking atmospheric, terrestrial and aquatic environments: Regime shifts in the Estonian climate over the past 50 years
Source: PLoS One. 2018 Dec 27;13(12):e0209568. doi: 10.1371/journal.pone.0209568 (PMC6307728; doi:10.1371/journal.pone.0209568)
Supplement: S2 Table — (PDF) [file pone.0209568.s002.pdf]

S2 Table. A R script on how to depict a timing and relative strength of statistically significant RSs and the relatedness of time series within each system category (i.e. abiotic and biotic elements of atmospheric, terrestrial, bog, lake, river and marine systems) in terms of the timing of RSs.

```
#Start of the script

#define objects for storing results
#kategooriad is a character vector with category names
abi=rep(NA,length(kategooriad)) #number of clusters within a system category i.e. abiotic and biotic elements of atmospheric, terrestrial, bog, lake, river and marine systems
names(abi)=kategooriad
kohad=list() #location of CPs within system categories (i.e. blocks)
pvalues=list() #respective p-values
tulemused=list() #clustering results of categories
yksikutecp=list() #number of CPs in single time series
cpkattuvused=list() #what is the percentage of single time series having CP within +/- 2 years; size of the square on fig2
kooskola=rep(NA,length(kategooriad)) #average mod.Agresti concordance index (single time series vs block)
kooskola_ilmavalja=rep(NA,length(kategooriad)) #average mod.Agresti concordance index (single time series vs block) discarding single series without CPs;line width on fig2
#end of object definition

#clustering part, blocks are analyzed one-by-one
 for (j in 1:length(kategooriad)){
	#first the whole block
	print(j)
	klass=kategooriad[j]
	# abiandmed is the respective block dataset
	tulemus=e.divisive(as.matrix(abiandmed), R = 5999, min.size = minnihe) #block clustering result
	tulemused[[j]]=tulemus #storing the result
	kohad[[j]]=tulemus$estimates+1965 #cp locations; 1 stands for the year 1966
	pvalues[[j]]=tulemus$p.values #respective p-values
	abi[j]=tulemus$k.hat #number of clusters
	#then the individual time series within the block
	abivek=lapply(seq_len(ncol(abiandmed)), function(i)
	as.matrix(abiandmed[,i],ncol=1)) #as a list, each element is a column vector of a single  series
	klasterdus=lapply(abivek, e.divisive,min.size = minnihe,R=5999) #single time series clustering within a block
	oiged=which(unlist(lapply(klasterdus,function(i){length(i$estimates)>2}))) #single time series with detected CPs
	yksikud=lapply(1:length(klasterdus), function(i) klasterdus[[i]]$estimates+1965)
	#years of single time series CPs
	yksikutecp[[j]]=sapply(klasterdus,function(i){i$k.hat-1}) #number of  Cps
	#calculating the concordance indices
	CPd=NULL #discarding first and last
	leidub=NULL #proportion of occurence in sigle time series
```

```
	if(length(kohad[[j]]>2)){
		CPd=kohad[[j]][2:(length(kohad[[j]])-1)]
		leidub=rep(NA,length(CPd))
		for (s in 1:length(CPd)){
			leidub[s]=mean(unlist(lapply(yksikud,function(j){CPd[s]%in%c(j-2,j-
			1,j,j+1,j+2)})))
		}
	}
	cpkattuvused[[j]]=leidub
	kooskola[j]=mean(unlist(lapply(klasterdus,function(i)
	{adjustedRand(i$cluster,tulemus$cluster)[3]})))
	kooskola_ilmavalja[j]=mean(unlist(lapply(klasterdus[oiged],function(i)
	{adjustedRand(i$cluster,tulemus$cluster)[3]})))
}
```

#fig2

```
par(mar=c(5.1,7.1,4.1,2.1))
plot(x=2,ylim=c(-9,-1),xlim=c(1966,2013),type="n",yaxt="n",ylab="",xlab="Year")
abline(v=c(1970,1980,1990,2000,2010),lty=2,col="grey",lwd=0.3)
for (i in 1:length(jrk)){
lines(x=c(1966,2013),y=c(-i,-i),lwd=10*kooskola_ilmavalja[jrk[i]])
}
names(abi)=gsub("\\.", " ", names(abi))
axis(2,-1:-9,names(abi)[jrk],las=1)
for (i in 1:length(jrk)){
if(all(kohad[[jrk[i]]]%in%c(1966,2014))){next}
points(x=kohad[[jrk[i]]][2:(length(kohad[[jrk[i]]])-1)],y=rep(-i,length(kohad[[jrk[i]]])-
2),pch=".",cex=30*(0.2+cpkattuvused[[jrk[i]]]))
}
```
